# Nanocatalysed green synthesis of benzo[*g*]chromene derivatives: DFT, docking, molecular dynamics and biological evaluation

**DOI:** 10.1039/d6ra05223a

**Published:** 2026-08-18

**Authors:** Sathiaseelan Perumal, Eswar Rao Tatta, Kiran Kumar Nalla, Perumal Muthuraja, Venkatesan Muthukumar, Paramasivam Manisankar, Viswanathan Subramanian

**Affiliations:** a Department of Industrial Chemistry, Alagappa University Karaikudi Tamil Nadu 630 006 India manisankarp@alagappauniversity.ac.in viswanathans@alagappauniversity.ac.in; b Department of Chemistry, Bishop Heber College Tiruchirappalli Tamil Nadu 620 017 India; c School of Life Sciences, Department of Biotechnology, Pondicherry University Puducherry-605014 India; d School of Life Sciences, University of Hyderabad Hyderabad-500046 Telangana India; e Department of Chemistry, Indian Institute of Technology Guwahati Guwahati-781 039 India; f Department of Chemistry, School of Advanced Sciences, VIT Vellore Tamil Nadu 632014 India

## Abstract

A sustainable one-pot multicomponent protocol was developed for the synthesis of benzo[*g*]chromene derivatives using Ag_2_WO_4_ nanoparticles as a recyclable catalyst. This represents the first application of Ag_2_WO_4_ nanocatalysis to this scaffold, affording the products in 86–95% yields under mild aqueous ethanol conditions. The synthesised compounds were characterised using FT-IR, NMR, and mass spectrometry and evaluated for anticancer and antibacterial activities. Compound 4f exhibited the highest cytotoxicity against HeLa and MDA-MB-231 cells, with 48 h IC_50_ values of 36.8 and 36.9 µM, respectively. In contrast, 4a exhibited the strongest preliminary antibacterial activity against *Acinetobacter baumannii* (MIC = 70 µg mL^−1^). Complementary DFT, docking, molecular dynamics, and ADME analyses provided insights into their electronic properties, predicted binding behaviour, and pharmacokinetic limitations. Overall, this study establishes Ag_2_WO_4_ nanoparticles as an effective catalyst for sustainable benzo[*g*]chromene synthesis and identifies 4f and 4a as promising scaffolds for further biological optimisation.

## Abstract 2

A sustainable one-pot multicomponent protocol was developed for the synthesis of benzo[*g*]chromene derivatives using Ag_2_WO_4_ nanoparticles as a recyclable catalyst.

## Graphical abstract

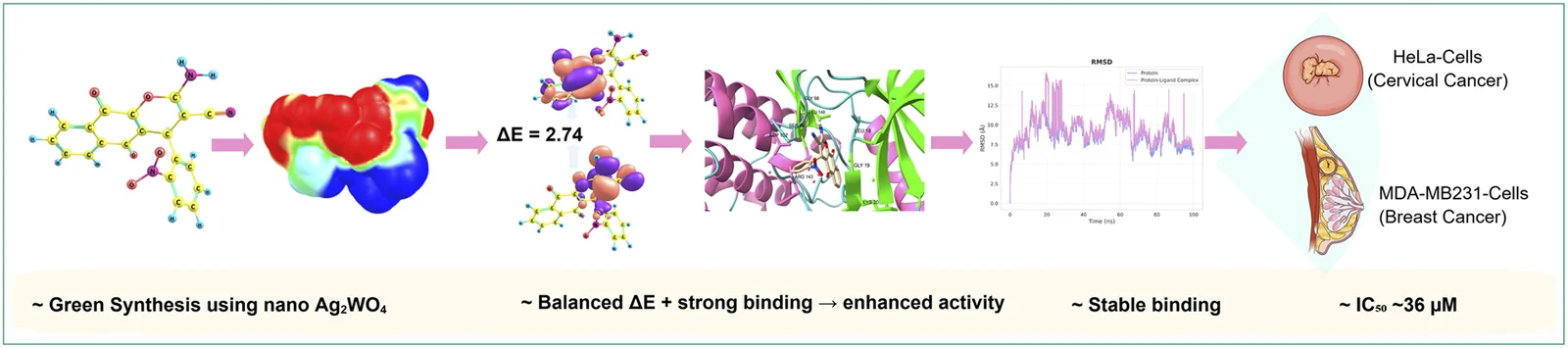

## 1 Introduction

Cancer is one of the leading causes of mortality worldwide, posing a major challenge to public health systems and socioeconomic development. In particular, breast and cervical cancers^1^ cause a significant global health burden, especially in developing countries.^2^ GLOBOCAN 2020 data indicate that mortality rates for female breast and cervical cancers are considerably higher in transitioning countries,^3^ many of which are classified as developing nations. Another important health problem in the medical field is the increasing resistance of *Acinetobacter baumannii* to conventional antimicrobial agents, which poses a significant clinical challenge, particularly in hospital settings.^4^ The World Health Organization has classified carbapenem-resistant *A. baumannii* as a priority pathogen of critical concern because of its limited treatment options and high mortality rates.^5^

In this context, fused oxygen-containing heterocycles have attracted attention in medicinal chemistry because of their extensive range of biological activities and chemical diversity.^6^ Among them, benzo[*g*]chromenes are an important subclass of oxygen-rich polycyclic compounds, which are known to exhibit diverse biological properties, such as antioxidant,^7^ anticancer,^8^ anti-inflammatory,^9^ and antimicrobial^10^ activities. The pharmacological significance of the benzo[*g*]chromene scaffold arises from its extended π-conjugated system,^11^ carbonyl functionality,^12^ diverse substitution patterns, which facilitate favourable interactions with biological targets. Additionally, the rigid aromatic framework and oxygen-containing heterocyclic ring provide a favourable balance of rigidity, polarity, and lipophilicity, which may further enhance biological activity.^13^

Therefore, the development of efficient and sustainable synthetic methods for benzo[*g*]chromene derivatives may support drug discovery efforts while promoting cost-effective and environmentally responsible research, particularly in developing countries. In sustainable synthesis, multicomponent one-pot reactions are primarily employed and are regarded as advantageous because of their capacity to generate a variety of structures, their simplicity of operation, and their atom economy.^14^ Various catalytic systems, including TEA,^15^ Et_3_NH[HSO_4_],^16^ CeO_2_/CuO @ N-GQDs @ NH_2_,^17^ piperidine,^18^ Amberlite IRA 400-Cl,^19^ Fe_3_O_4_@SiO_2_@NH_2_-TCT-Mesalamine-Cu(ii) MNPs,^20^ Cu(OTf)_2_,^21^l-proline,^22^ Dy@ HaI-SS-g-C_3_N_4_,^23^ Fe_3_O_4_/PEG,^24^ Magnetic @ UiO-66-NH-SB-Cu(ii),^25^ ZnO–Co_3_O_4_ NC,^26^ and ZNO–Co_3_O_4_,^27^ have been explored for this reaction.

Although various alternatives are available, many have been developed at the cost of potentially limiting factors, including extended reaction times, expensive catalysts, and the use of chlorinated organic solvents. Consequently, there has been growing interest in developing eco-friendly and time-efficient synthetic methodologies. Therefore, building on our earlier work, in which Ag_2_WO_4_ nanoparticles demonstrated effective catalytic performance in the synthesis of pyrano[2,3-*d*]pyrimidinones within a short reaction time,^28^ we explored their application in the synthesis of benzo[*g*]chromene derivatives. In this study, Ag_2_WO_4_ was used as a nanocatalyst in an ethanol–water medium to establish a simple, efficient, and environmentally benign synthetic protocol.

In the field of pharmaceutics, the time required to translate a laboratory discovery into a marketable product largely depends on the identification of promising chemical entities.^29^ With this in mind, we integrated the development of sustainable synthetic methods with modern *in silico* methods, such as ADME, DFT, molecular docking, and MD simulations (PCA, FEL, and MM/GBSA), to gain insights into molecular reactivity, electronic structure, and target interactions. These computational tools are increasingly employed in drug discovery to guide experimental studies, reduce development costs, and support sustainable research practices.^30^

## 2 Results and discussion

### 2.1 Chemistry

#### 2.1.1 Characterisation of the Ag_2_WO_4_ nanocatalyst

The Ag_2_WO_4_ nanocatalyst was prepared according to a previously reported procedure.^28^ The structural, morphological, and elemental characteristics were confirmed using X-ray diffraction (XRD), scanning electron microscopy (SEM), and energy-dispersive X-ray spectroscopy (EDX). As the same catalyst has been comprehensively characterised in our earlier work, a concise description of the characterisation results, together with the corresponding XRD, SEM, EDX, and elemental-mapping data, is provided in the SI (Sections S5.1–S5.2, Fig. S34 and S35). In the present study, XPS analysis was performed to examine the surface elemental composition and chemical states of the Ag_2_WO_4_ nanoparticles. The survey spectrum confirmed the presence of Ag, W, and O. In the high-resolution Ag 3d spectrum, peaks at 367.94 and approximately 373.9 eV were assigned to Ag 3d_5/2_ and Ag 3d_3/2_, respectively, consistent with the presence of silver in Ag_2_WO_4_. The W 4f spectrum appeared in the approximately 35–38 eV region, with a principal peak at 36.01 eV, supporting the presence of W^6+^ containing tungstate species. The O 1s spectrum exhibited a dominant component at 530.29 eV, attributable primarily to lattice oxygen in the Ag–O and W–O bonds, together with higher binding energy contributions associated with surface hydroxyl or adsorbed oxygen species. Overall, the XPS results confirmed the presence of Ag, W, and O on the surface of the prepared Ag_2_WO_4_ nanoparticles and their expected chemical environment. The corresponding XPS survey and high-resolution spectra are provided in the SI (Section S5.3, Fig. S36).

#### 2.1.2 Proposed mechanism and substrate scope

A one-pot multicomponent reaction involving 2-hydroxynaphthalene-1,4-dione, malononitrile, and substituted aromatic aldehydes, catalysed by Ag_2_WO_4_ nanocatalysts, was used to synthesise 4*H*-benzo[*g*]chromene derivatives (4a–4i) (Table 1). The reaction was optimised, and the results are summarised in Table 8. The desired products were obtained as precipitates in good to excellent yields (86–95%) within 40 min in an ethanol–water (1 : 1) medium at 70 °C. In contrast, the catalyst-free reaction required 6 h and afforded only 70% yield. The results demonstrate the operational simplicity and effectiveness of the developed protocol. Moreover, this study may pave the way for the development of novel and sustainable synthetic methodologies.

**Table 1:** Synthesis of 2-amino-4*H*-benzo[*g*]chromenes derivatives

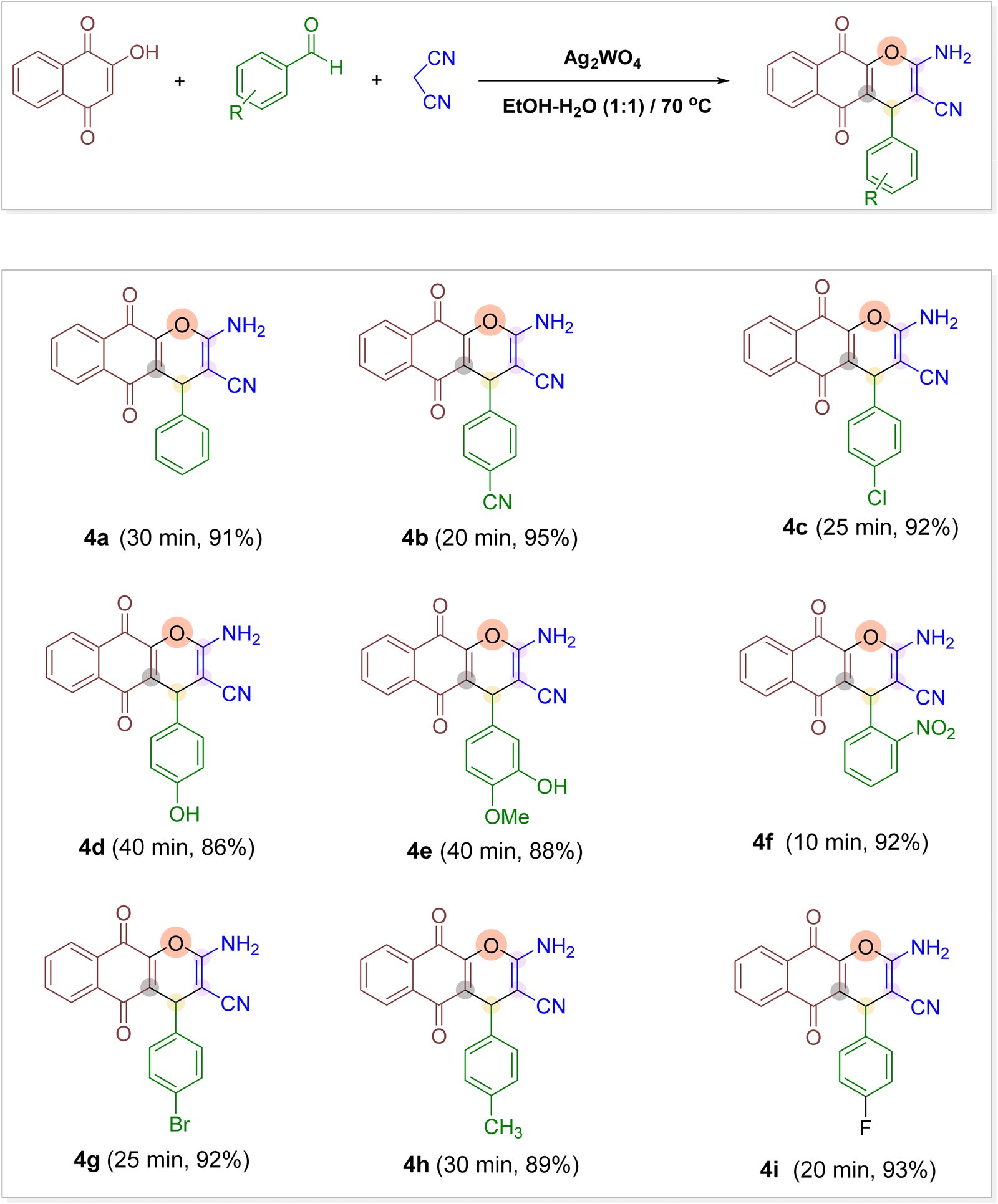

Based on literature reports describing the bifunctional catalytic activity of Ag_2_WO_4_,^31^ a plausible mechanism for the synthesis of 4*H*-benzo[*g*]chromene derivatives is proposed in Scheme 1. Malononitrile is activated to form a carbanion in the presence of the basic oxygen sites of the tungstate anion, which subsequently reacts with the aldehyde *via* Knoevenagel condensation facilitated by Lewis acidic Ag^+^ centres. This step generates an arylidene malononitrile intermediate, which undergoes Michael addition with nucleophilic 2-hydroxynaphthalene-1,4-dione. Based on the cited literature, the Ag_2_WO_4_ system may provide complementary Lewis acidic and basic surface sites. The resulting adduct undergoes intramolecular cyclisation and tautomerization to afford a 4*H*-benzo[*g*]chromene structure. The heterogeneous nature of Ag_2_WO_4_, along with its surface acidity and basicity, is likely to enhance the reaction rate. However, because the proposed intermediate and individual surface activation steps were not directly examined, this pathway should be regarded as a literature-supported mechanistic proposal, rather than a conclusively established mechanism.

**Scheme 1:**
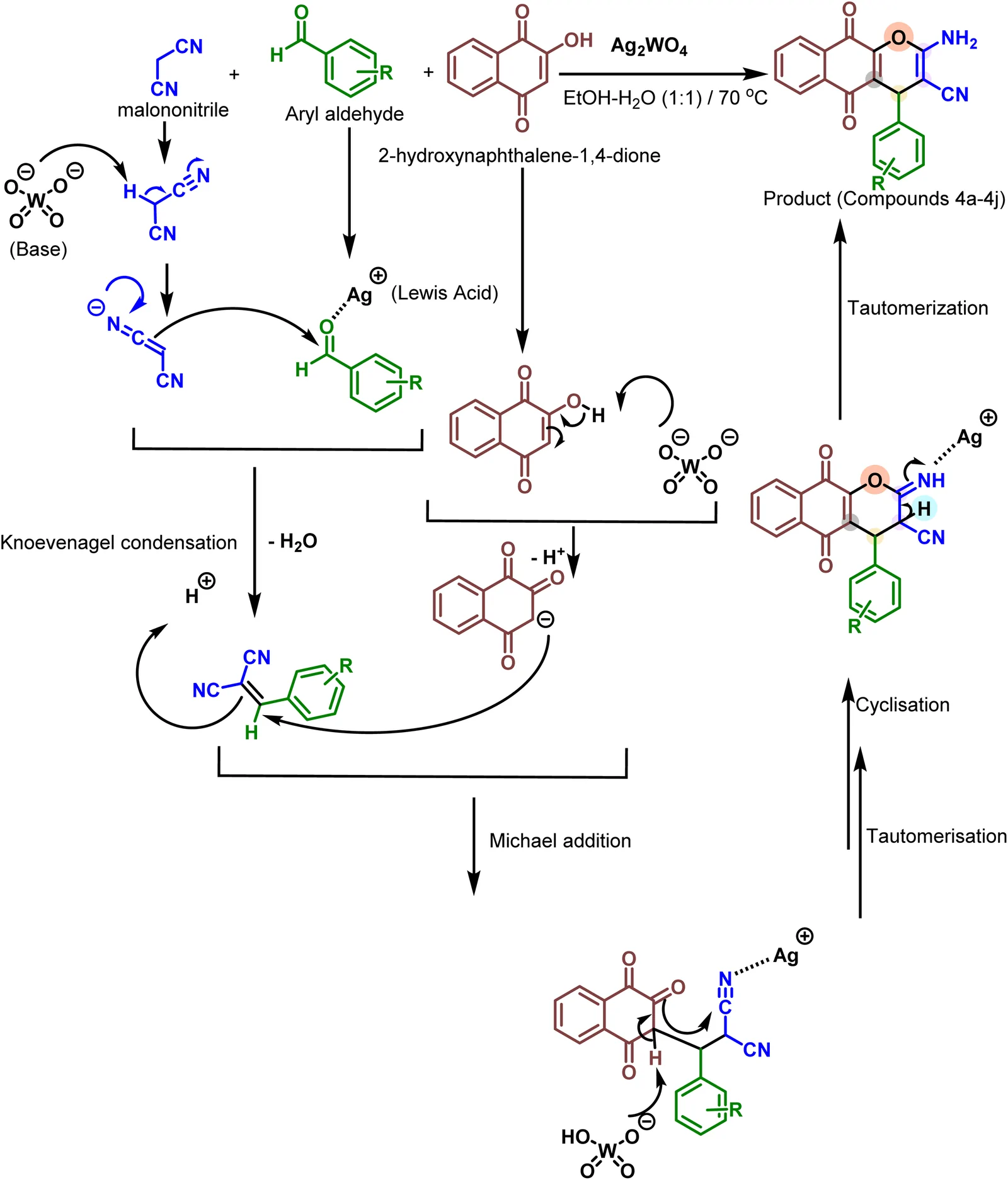
Proposed mechanism for the Ag_2_WO_4_-catalyzed synthesis of 4*H*-benzo[*g*]chromenes.

The influence of the substituents on the aromatic aldehyde was evident from the observed yields. Electron-withdrawing groups (EWGs) such as –NO_2_ (4f), –Cl (4c), –CN (4b), and –F (4i) generally afforded higher yields (up to 95%), which can be attributed to the increased electrophilicity of the aldehyde carbonyl, thereby facilitating the initial nucleophilic attack on it. In contrast, electron-donating groups (EDGs), such as –OH (4d) and –OCH_3_ (4e), led to reduced yields, likely because of the reduced electrophilic character of the aldehyde. However, these substrates still provided satisfactory yields, indicating the robustness of the catalytic system employed.

The recyclability of the Ag_2_WO_4_ nanocatalyst was evaluated over five consecutive cycles. The yield of the isolated product gradually decreased from 95% in the first run to 84% in the fifth run (Fig. S37), corresponding to approximately 88% of the initial product yield. This decline may have arisen from catalyst loss during recovery, surface fouling, agglomeration, or changes in the active surface.

To further assess the practical significance of the present protocol, the catalytic performance of the Ag_2_WO_4_ nanoparticles was compared with that of representative reported methods for the same benzo[*g*]chromene-forming transformation (Table S2). The comparison considered catalyst loading, solvent, temperature, reaction time, isolated yield, recyclability, and availability of TON and TOF data. Although some reported methods provide comparable yields or operate under milder conditions, the present protocol offers a favourable combination of low catalyst loading (2.5 mol%), an EtOH/H_2_O (1 : 1) medium, a reaction time of approximately 20 min, high isolated yields, and catalyst recovery and reuse over five cycles. Thus, the significance of this method lies in its combined operational and catalytic performance rather than solely in the first use of Ag_2_WO_4_ for this transformation.

#### 2.1.3 Quantitative green chemistry assessment

To quantitatively assess the environmental performance of the developed protocol, green chemistry metrics were calculated for the synthesis of model compound 4b under optimised conditions. The assessment included atom economy, carbon efficiency, reaction mass efficiency, process mass intensity (PMI), and *E*-factor. The reaction-stage and complete-process metrics were calculated separately, and the influence of including or excluding water was considered. The calculated values are presented in Table 2, and the detailed equations, material inventory, and calculation boundaries are provided in the SI (Section S5.4). The high atom economy, carbon efficiency, and reaction mass efficiency indicate the efficient incorporation of the reactants into the isolated product. However, the complete-process PMI and *E*-factor were increased by DMSO and ethanol employed during isolation and catalyst washing. Therefore, the present protocol possesses several favourable green chemistry features, although optimising solvent use would further improve its overall material efficiency.

**Table 2:** Green chemistry metrics calculated for the Ag_2_WO_4_-catalysed synthesis of model compound 4b

Metric	Value
Atom economy	95.15%
Carbon efficiency	95.0%
Reaction mass efficiency	90.38%
Reaction-stage PMI, including water	12.73
Reaction-stage *E*-factor, including water	11.73
Complete-process PMI, including water	24.56
Complete-process *E*-factor, including water	23.56
Complete-process PMI, excluding water	18.08
Complete-process *E*-factor, excluding water	17.08
Apparent TON	38
Apparent TOF	114 h^−1^

In summary, the nano Ag_2_WO_4_ catalyzed protocol offers an efficient and green synthetic methodology for the synthesis of benzo[*g*]chromene derivatives with good performance over a range of substituents. The structures of 4a–4i were confirmed by FT-IR, ^1^H and ^13^C NMR, and HRMS analyses, which were in agreement with the proposed molecular structures of the compounds. The spectra of the synthesised compounds (4a–4i) are provided in the SI (Fig. S1–S27). The synthesised compounds were then subjected to antibacterial screening and *in vitro* cytotoxic evaluation to assess their biological potential.

### 2.2 Antibacterial activity

The antibacterial activity of the synthesised benzo[*g*]chromene derivatives (4a–4i) was evaluated against *Acinetobacter baumannii* (ATCC 19606), and the results are presented in Table 3. The synthesised derivatives exhibited antibacterial activity against *A. baumannii*, with MIC values ranging from 70 to 100 µg mL^−1^. Among the series, compound 4a exhibited the highest activity, with the lowest MIC value of 70 µg mL^−1^, followed by compound 4i with an MIC value of 80 µg mL^−1^. Compounds 4b, 4c, 4d, 4f, 4g, and 4h exhibited MIC values of approximately 90 µg mL^−1^, indicating comparable activity across the series. As a reference antibiotic was not evaluated concurrently under identical assay conditions, these findings should be regarded as preliminary evidence of antibacterial activity, and no comparison with standard antibiotics was made (Fig. 1).

**Table 3:** Minimum inhibitory concentrations (MIC, µg mL^−1^) of synthesised benzo[*g*]chromene derivatives (4a–4i) against *Acinetobacter baumannii* (ATCC 19606)

Compound code	4a	4b	4c	4d	4e	4f	4g	4h	4i
MIC (µg mL^−1^)	70	90	90	90	100	90	90	90	80

**Fig. 1:**
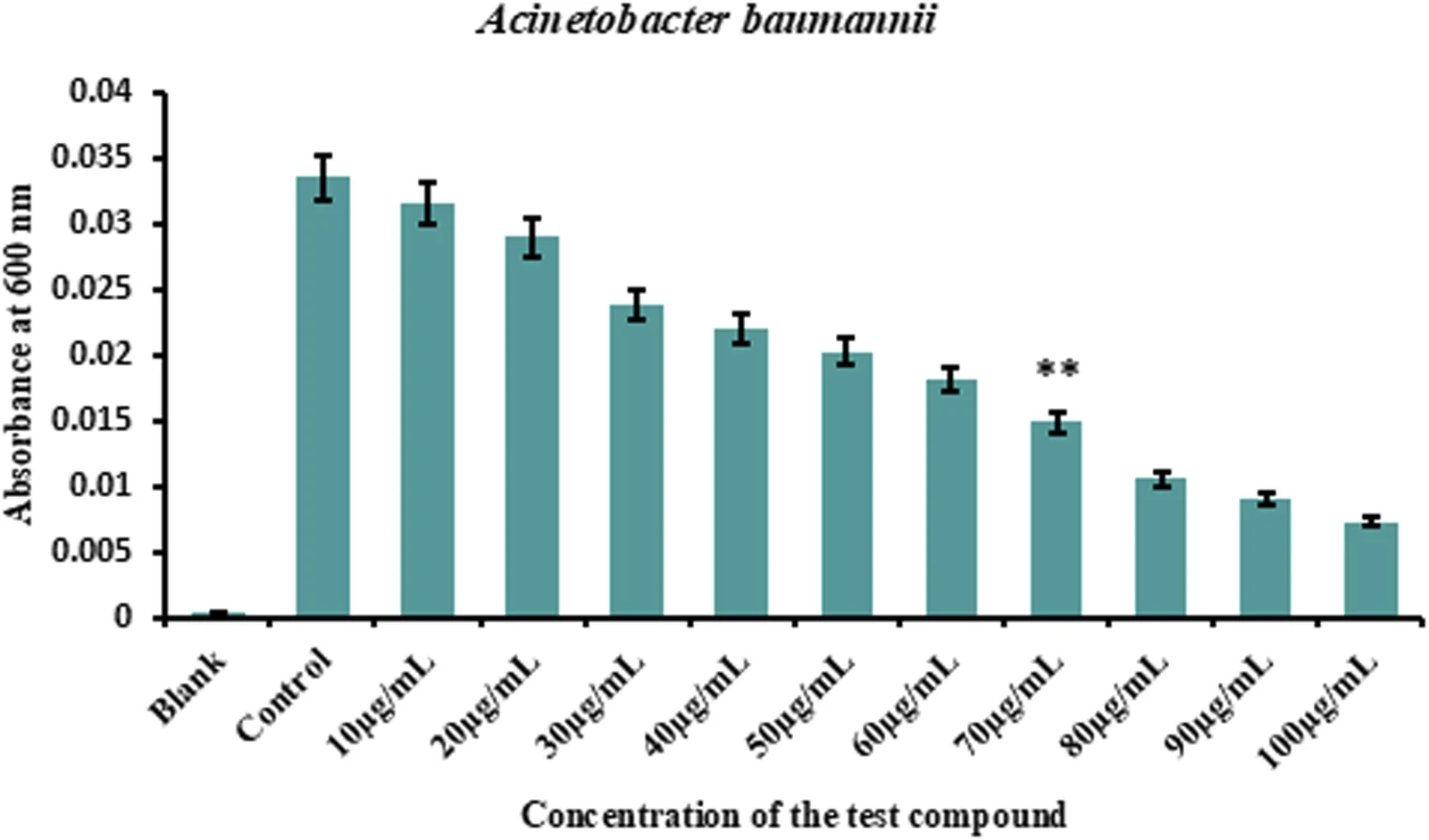
Effect of compound 4a on the growth of *A. baumannii* ATCC 19606 at different concentrations, showing concentration-dependent reduction in OD_600_ absorbance. Significant inhibition was observed at 70 µg mL^−1^.

### 2.3 *In vitro* cytotoxicity evaluation by MTT assay

The cytotoxic activity of the synthesised benzo[*g*]chromene derivatives (4a–4i) was evaluated against HeLa and MDA-MB231 cell lines using the MTT assay, while HEK293 cells were included as a normal cell control to assess selectivity. Untreated cells were used as the negative control, and doxorubicin was used as the positive control. All experiments were performed in three independent biological experiments, each conducted in triplicate, and the results were expressed as IC_50_ values (mean ± SD). The results presented in Table 4 indicate that compound 4f (Fig. 2) exhibited the highest cytotoxic activity among the synthesised derivatives, with IC_50_ values of 36.8 ± 1.9 SD µM and 36.9 ± 1.8 SD µM against HeLa and MDA-MB231 cells, respectively, after 48 h of treatment. Compounds 4e and 4c (Fig. S31 and S32) also demonstrated moderate cytotoxic activity, with IC_50_ values of 41.3 ± 1.2 SD and 44.2 ± 1.2 SD µM against HeLa cells, respectively, while both compounds showed an IC_50_ value of 42.3 ± 1.6 SD µM against MDA-MB231 cells after 48 h. The remaining derivatives (4a, 4b, 4d, 4g, 4h, and 4i) exhibited IC_50_ values greater than 70 µM, indicating comparatively lower cytotoxic activity.

**Table 4:** *In vitro* cytotoxicity (IC_50_, µM) of the synthesised benzo[*g*]chromene derivatives (4a–4i) was evaluated against HeLa, MDA-MB231, and HEK293 cell lines at 24 and 48 h using the MTT assay^a^

S. no.	Compound code	IC_50_ values @ 24 h in µM			IC_50_ values @ 48 h in µM		
		MDA-MB231	HeLa	Hek293	MDA-MB231	HeLa	Hek293
1	4a	78.6 ± 1.2	77.9 ± 1.6	99.4 ± 1.1	76.4 ± 1.2	73.4 ± 1.1	97.3 ± 1.1
2	4b	84.5 ± 1.3	79.7 ± 1.8	98.4 ± 1.4	78.3 ± 1.3	77.3 ± 1.0	97.6 ± 1.0
3	4c	44.3 ± 1.2	46.7 ± 0.8	97.9 ± 1.7	42.3 ± 1.6	44.2 ± 1.2	92.9 ± 1.2
4	4d	79.5 ± 1.5	80.3 ± 1.7	98.3 ± 1.6	76.3 ± 1.8	74.5 ± 1.9	94.9 ± 1.7
5	4e	48.9 ± 1.8	46.3 ± 1.7	97.4 ± 1.7	42.3 ± 1.7	41.3 ± 1.2	92.9 ± 1.8
6	4f	40.4 ± 1.2	39.8 ± 1.2	96.5 ± 1.6	36.9 ± 1.8	36.8 ± 1.9	93.8 ± 1.8
7	4g	86.7 ± 1.7	83.4 ± 1.8	94.3 ± 1.6	84.3 ± 1.5	81.9 ± 1.9	91.3 ± 1.7
8	4h	88.9 ± 1.1	87.8 ± 1.7	96.3 ± 1.8	87.3 ± 1.2	82.7 ± 1.7	94.2 ± 1.4
9	4i	86.5 ± 1.8	87.4 ± 2.1	97.3 ± 1.2	82.9 ± 1.7	84.3 ± 1.6	94.8 ± 1.2
10	Doxorubicin	26.4 ± 1.9	27.6 ± 1.8	96.3 ± 1.2	22.5 ± 1.1	25.2 ± 1.8	88.9 ± 2.1

a ± = standard deviation values.

**Fig. 2:**
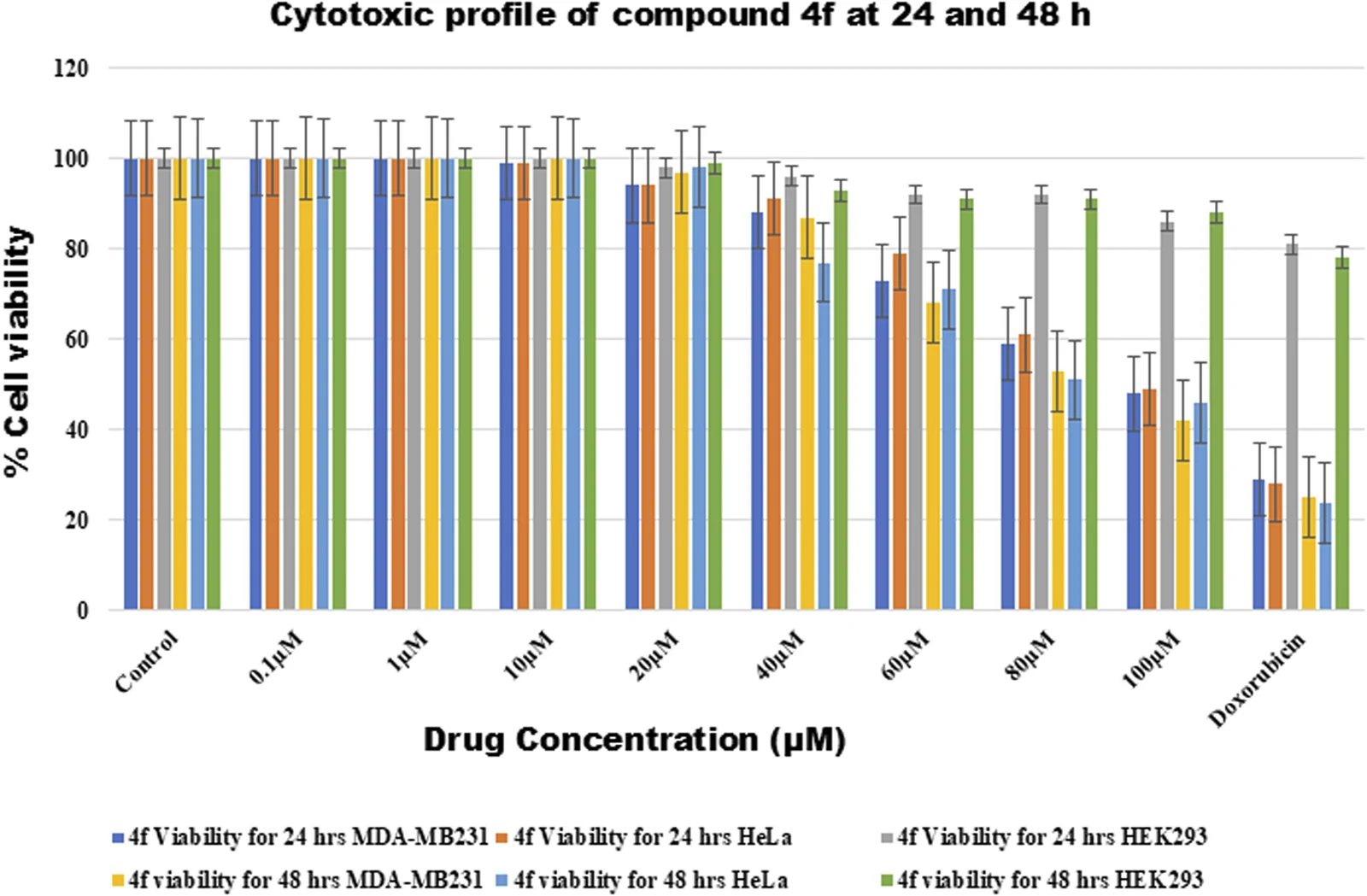
Cell viability graph of 4f compound for MDA-MB231, HeLa and Hek293 cells for 24 and 48 h.

As expected, the reference drug doxorubicin displayed greater antiproliferative activity, with IC_50_ values of 25.2 ± 1.8 SD µM against HeLa cells and 22.5 ± 1.1 SD µM against MDA-MB231 cells after 48 h. Although the synthesised compounds showed moderate cytotoxic potency relative to doxorubicin, they exhibited substantially higher IC_50_ values against HEK293 cells (91–99 µM), suggesting lower toxicity toward normal cells and selectivity for cancer cells. These findings indicate that 4f is the most promising lead in the present series and may serve as a useful scaffold for further structural optimisation to improve its anticancer potency. To gain further insight into the observed biological activity, density functional theory (DFT) studies were performed, and the results are discussed in the following section.

### 2.4 Density functional theory (DFT) calculations

Density functional theory (DFT) calculations were conducted to investigate the electronic characteristics and reactivities of the synthesised benzo[*g*]chromene derivatives (4a–4i) to evaluate the charge distribution, reactive sites, and donor–acceptor interactions, frontier molecular orbitals (FMO), molecular electrostatic potential (MEP), and natural bond orbitals (NBO) analyses were performed.

#### 2.4.1 Frontier molecular orbital (HOMO–LUMO) analysis

The electronic properties of the synthesised benzo[*g*]chromene derivatives (4a–4i) were investigated using DFT calculations, and the corresponding quantum chemical descriptors are summarised in Table 5. The HOMO–LUMO energy gaps (Δ*E*) ranged from 2.40 to 2.81 eV, indicating moderate substituent-dependent variation in the electronic characteristics of the benzo[*g*]chromene scaffold. The order of energy gap was 4e < 4d < 4f < 4h < 4a = 4g < 4c = 4i < doxorubicin < 4b < tetracycline. Compound 4e exhibits the lowest Δ*E* (2.40 eV), whereas 4b shows the highest value among the synthesised derivatives (2.81 eV). Compound 4f, which exhibited the highest cytotoxic activity after 48 h of exposure, possessed an intermediate energy gap of 2.74 eV (Fig. 3). Therefore, the cytotoxicity ranking did not directly follow the HOMO–LUMO energy gap order.

**Table 5:** Calculated quantum chemical parameters of the synthesised compounds (4a–4i), tetracycline, and doxorubicin^a^

Code	*E*_LUMO_ (eV)	*E*_HOMO_ (eV)	Δ*E*	(*I*)	(*A*)	(*η*)	(*z*)	(*χ*)	(*µ*)	(*ω*)	(*D*)
4a	−3.54	−6.31	2.77	6.31	3.54	1.39	0.36	4.93	−4.93	8.76	4.95
4b	−3.80	−6.61	2.81	6.61	3.80	1.41	0.36	5.21	−5.21	9.64	8.06
4c	−3.63	−6.41	2.78	6.41	3.63	1.39	0.36	5.02	−5.02	9.06	5.62
4d	−3.51	−6.13	2.62	6.13	3.51	1.31	0.38	4.82	−4.82	8.87	4.49
4e	−3.44	−5.84	2.40	5.84	3.44	1.20	0.42	4.64	−4.64	8.97	4.52
4f	−3.53	−6.27	2.74	6.27	3.53	1.37	0.36	4.90	−4.90	8.76	2.78
4g	−3.64	−6.41	2.77	6.41	3.64	1.39	0.36	5.03	−5.03	9.12	5.69
4h	−3.51	−6.26	2.75	6.26	3.51	1.38	0.36	4.89	−4.89	8.68	4.88
4i	−3.61	−6.39	2.78	6.39	3.61	1.39	0.36	5.00	−5.00	8.99	5.48
Tetracycline	−2.26	−6.25	3.99	6.25	2.26	2.00	0.25	4.26	−4.26	4.54	4.65
Doxorubicin	−3.27	−6.06	2.79	6.06	3.27	1.40	0.36	4.67	−4.67	7.80	7.91

a *E*_LUMO_ = Energy of LUMO, *E*_HOMO_ = Energy of HOMO, Δ*E* = |*E*_HOMO_ − *E*_LUMO_|, *I* = Ionisation Potential, *A* = Electron affinity, *η* = Chemical Hardness, *z* = Chemical Softness, *χ* = Electronegativity, *µ* = Chemical Potential, *ω* = Electrophilicity, *D* = Dipole moment.

**Fig. 3:**
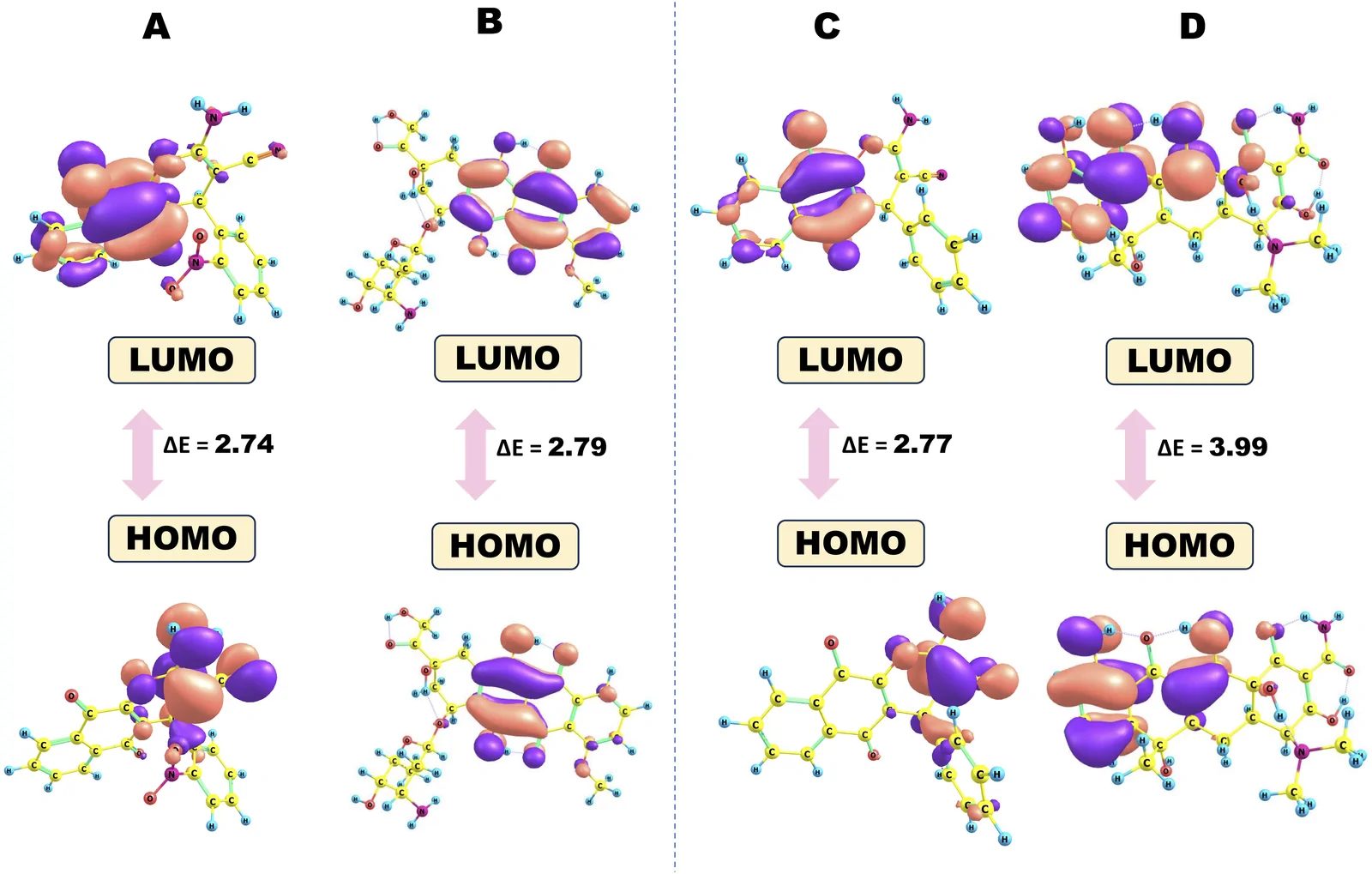
Frontier molecular orbital (HOMO–LUMO) distribution and energy gap (Δ*E*) of (A) 4f, (B) doxorubicin, (C) 4a, and (D) tetracyclin.

To examine the relationships between the calculated electronic properties and cytotoxic activity, exploratory Spearman rank correlation analysis was performed using the 48 h activity data of compounds 4a–4i. The IC_50_ values were converted into pIC_50_ values, and the correlations of cytotoxic activity with Δ*E*, electrophilicity index (*ω*), and dipole moment (*D*) were evaluated separately for the HeLa and MDA-MB-231 cell lines. The correlation coefficients are presented in Table S1. Chemical hardness and softness were not independently included in the analysis because they were mathematically derived from the HOMO–LUMO energy gap. For the HeLa cell line, Δ*E*, *ω*, and *D* showed Spearman correlation coefficients (*r*_s_) of −0.445, −0.176, and −0.483, respectively. Thus, Δ*E* and *D* exhibited moderate negative correlations with cytotoxic activity, whereas *ω* showed a weak negative correlation. For MDA-MB-231 cells, the corresponding *r*_s_ values were −0.359, −0.084, and −0.502, indicating a weak negative correlation for Δ*E*, a very weak negative correlation for *ω*, and a moderate negative correlation for *D*. Among the examined descriptors, dipole moment showed the most consistent relationship with cytotoxic activity across both cell lines. Negative correlations indicate that compounds with lower dipole moments tend to exhibit higher pIC_50_ values and, consequently, greater cytotoxic activity. Compound 4f, which had the lowest dipole moment in the series (2.78 *D*), exhibited the highest cytotoxic activity. Nevertheless, the moderate correlation coefficients indicate that the dipole moment alone does not completely account for the observed activity pattern.

Overall, the cytotoxic activity of the present series cannot be explained by Δ*E*, electrophilicity, or dipole moment individually. The biological response likely reflects the combined influence of electronic distribution, molecular geometry, substituent-dependent interactions, physicochemical behaviour, cellular uptake, and target engagement. Because the analysis involved only nine structurally related compounds, these correlations were interpreted as an exploratory structure–activity assessment rather than a predictive quantitative model.

The HOMO (−6.61 to −5.84 eV) and LUMO (−3.80 to −3.44 eV) energies showed moderate electron-donating and-accepting characteristics across the series. The comparatively narrow variations in the ionisation potential and electron affinity further indicate that aryl substitution primarily fine-tunes the electronic environment of the benzo[*g*]chromene scaffold. The optimised structures and frontier molecular orbitals of compounds 4a–4i, doxorubicin, and tetracycline are presented in Fig. S33.

#### 2.4.2 Molecular electrostatic potential (MEP) mapping

The molecular electrostatic potential (MEP) maps of 4f and 4a (Fig. 4) and the reference drugs were analysed to correlate the charge distribution with the observed biological activity. In the MEP surfaces, the red regions represent electron-rich areas with negative electrostatic potential, the blue regions indicate electron-deficient sites with positive potential, and the green regions correspond to relatively neutral or hydrophobic surfaces. In the MEP surface of compound 4f, the electron-rich regions (red) were concentrated around the nitro and carbonyl groups, suggesting that these functionalities may play an important role in hydrogen bonding and electrostatic interactions with cancer-associated targets.

**Fig. 4:**
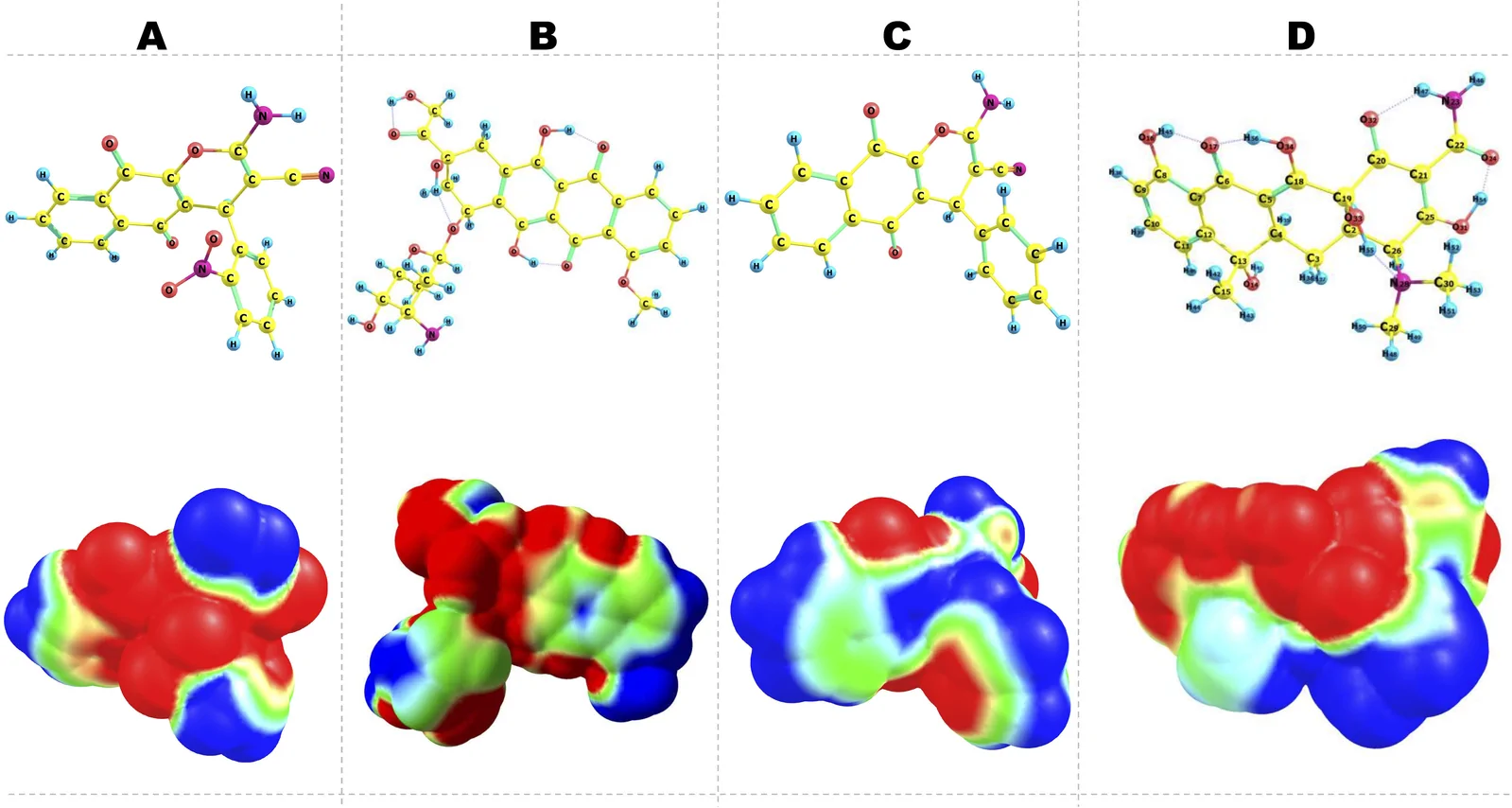
DFT-optimised geometries (top) and molecular electrostatic potential (MEP) maps (bottom) of (A) 4f, (B) doxorubicin, (C) 4a, and (D) tetracycline, illustrating the charge distribution across the molecular framework.

In 4a, the red regions are mainly localised around the quinone carbonyl oxygens (Fig. 4), while the phenyl and fused aromatic regions show broad green/yellow non-blue surfaces, indicating a strong hydrophobic character. This surface pattern is important for *A. baumannii* activity, as it may favour hydrophobic accommodation and reorientation within the bacterial binding pocket rather than relying only on polar interactions. In contrast, tetracycline shows more extensive red and blue polar zones due to multiple oxygenated and amine groups, supporting stronger hydrogen bonding/electrostatic interactions along with hydrophobic contacts. The molecular electrostatic potential (MEP) maps of compounds 4a–4i, doxorubicin, and tetracycline are presented in Fig. S33.

### 2.5 Molecular docking analysis

Molecular docking was performed to examine the predicted binding of benzo[*g*]chromene derivatives 4a–4i to the selected targets (Table 6). Overall, the compounds showed favourable binding across the three proteins. Compound 4f exhibited the strongest affinity among the derivatives toward the HeLa-associated target (5I9B), approaching that of doxorubicin. Its binding was supported by hydrogen bonding and aromatic interactions with residues including PHE250, SER251, PHE256, and TRP206 (Fig. 5A), consistent with its superior cytotoxic activity.

**Table 6:** Molecular docking binding energies (kcal mol^−1^) of the synthesised benzo[*g*]chromene derivatives (4a–4i) with selected targets: HeLa-associated protein (PDB: 5I9B), MDA-MB231-associated protein (PDB: 3KRR), and *Acinetobacter baumannii* target (PDB: 7PQM)

PDB ID	Binding energy (kcal mol^−1^)										
	4a	4b	4c	4d	4e	4f	4g	4h	4i	Tetracycline	Doxorubicin
5I9B (HeLa)	−8.2	−8.2	−7.7	−7.7	−8.0	−8.4	−7.7	−7.9	−8.2	—	−8.8
3KRR (MDA-MB231)	−7.7	−7.7	−7.4	−7.6	−7.3	−7.9	−7.5	−7.4	−7.8	—	−11.0
7PQM (*A.baumannii*)	−7.7	−8.7	−8.1	−7.8	−8.2	−7.6	−8.1	−8.1	−8.1	−6.8	—

**Fig. 5:**
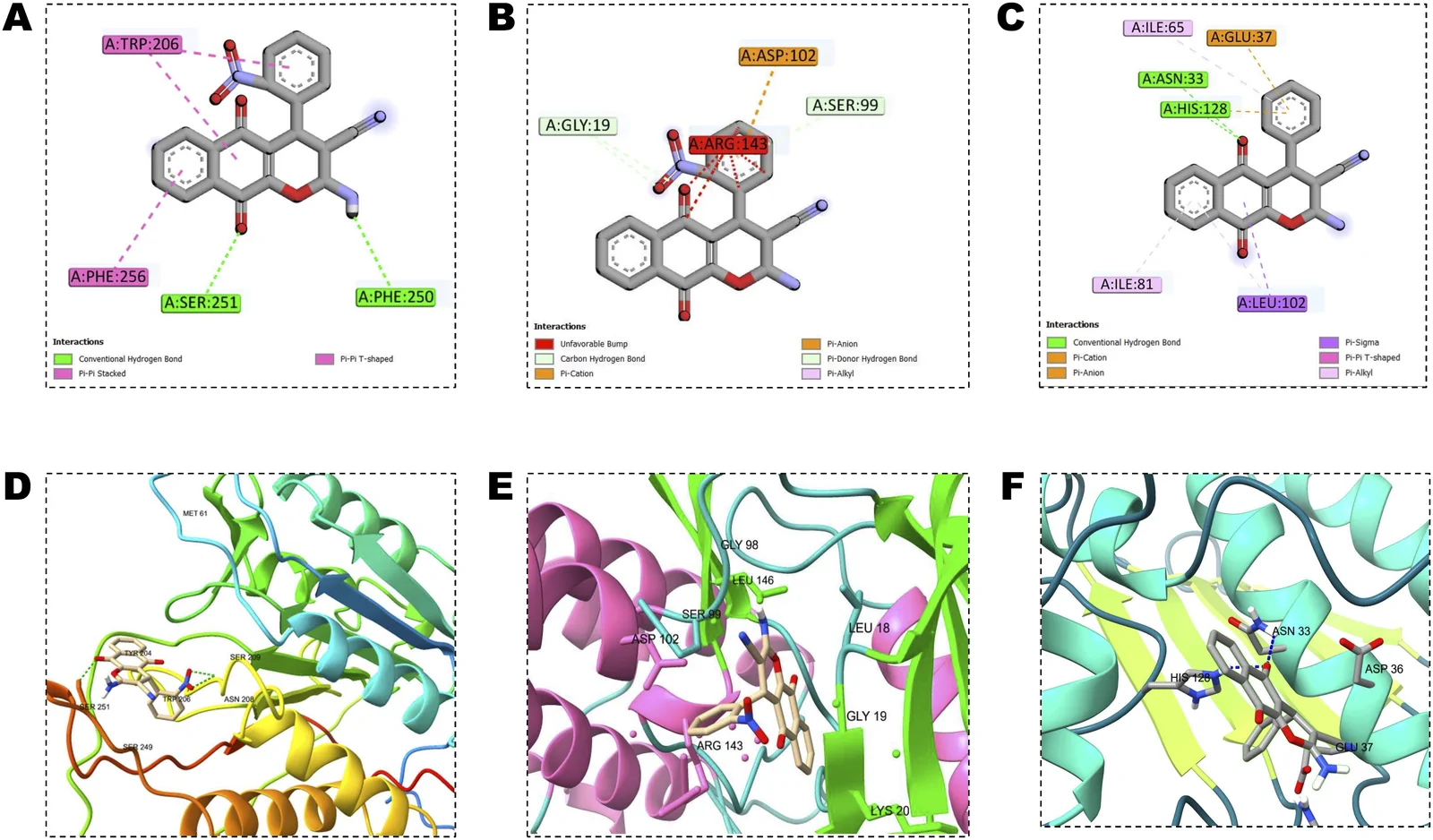
(A and D) 2D interaction and 3D binding visualisation of 4f with HeLa target (5I9B); (B and E) 4f with MDA-MB231 target (3KRR); (C and F) 4a with *A. baumannii* target (7PQM).

Against the MDA-MB-231-associated target (3KRR), the derivatives showed comparable binding, with 4f again giving the most favourable score. Hydrogen-bonding and electrostatic contacts with GLY19, SER99, and ASP102 stabilised its predicted binding mode (Fig. 5B). For the bacterial target 7PQM, 4b showed the strongest predicted affinity, whereas 4a was experimentally the most active derivative. Compound 4a interacted with ASN33, HIS128, and GLU37 (Fig. 5C). This mismatch indicates that antibacterial activity is not governed by docking affinity alone and may also depend on permeability, solubility, and intracellular availability.

The box plots in Fig. 6A–C showed generally consistent binding-energy distributions across the docking poses. Compounds 4f and 4g displayed compact distributions against the cancer-associated targets, while 4a showed consistent binding to **7PQM**. Overall, the docking results support plausible interactions with the selected proteins but do not fully reproduce the experimental activity ranking or establish the cellular mechanism of action.

**Fig. 6:**
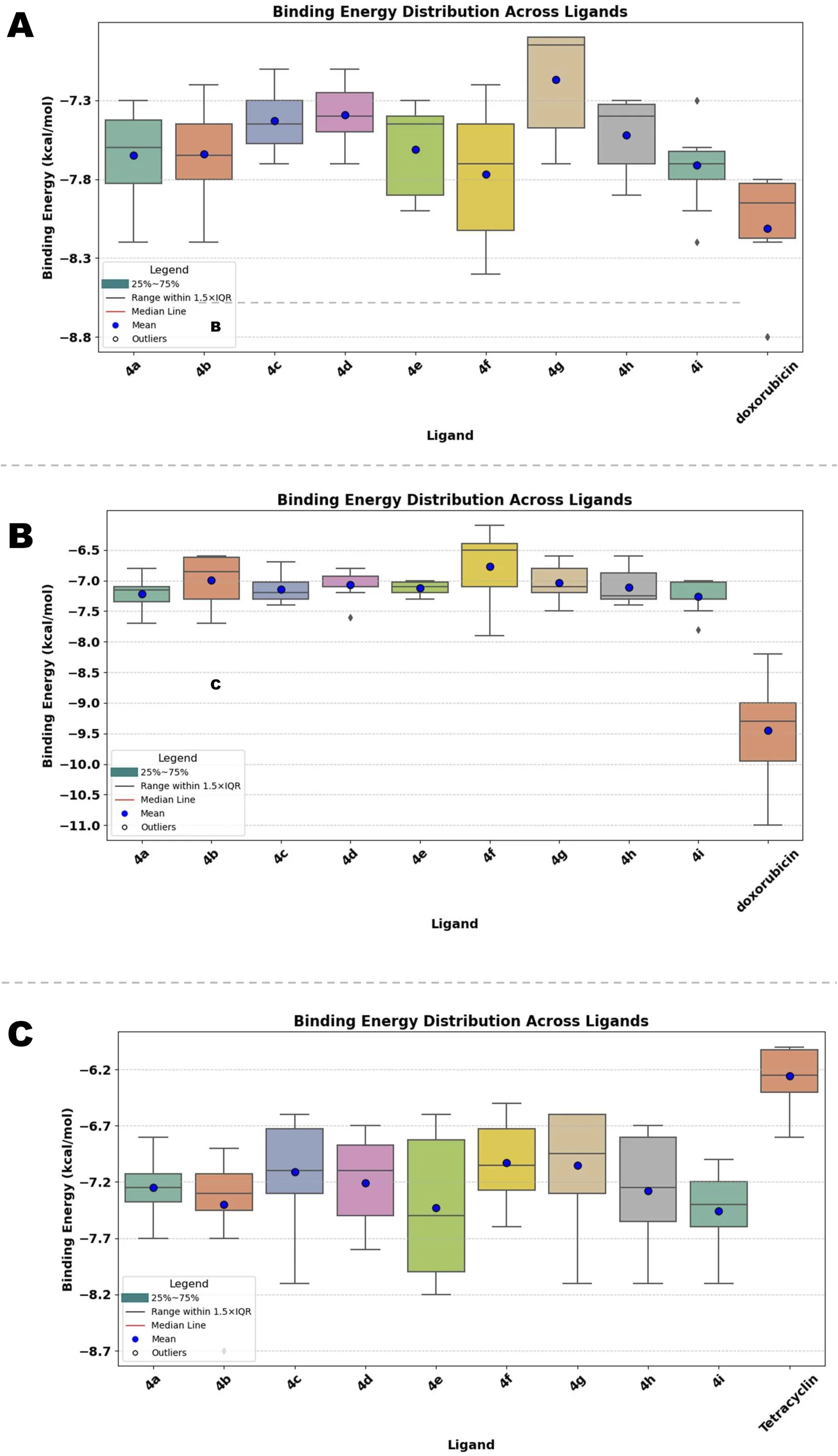
Box plot representation of binding energy (kcal mol^−1^) distributions for the synthesized benzo[*g*]chromene derivatives (4a–4i) across different targets: (A) HeLa-associated protein (PDB: 5I9B), (B) MDA-MB231-associated protein (PDB: 3KRR), and (C) *Acinetobacter baumannii* target (PDB: 7PQM). The plots depict the median, interquartile range (25–75%), and outliers, illustrating the consistency and variability of ligand binding across multiple docking poses. Compounds 4c, 4e, and 4f (cancer targets) and 4a (bacterial target) show comparatively stable binding profiles.

### 2.6 Molecular dynamics (MD) simulation, PCA, FEL and MM/GBSA analysis of 4f against 5I9B and 3KRR

Molecular dynamics simulations of 4f with 5I9B and 3KRR revealed stable protein–ligand interactions and favourable conformational behaviour throughout the simulation period. Further analyses using PCA, FEL, and MM/GBSA supported the dynamic stability and energetic favourability of binding toward both cancer-associated targets.

#### 2.6.1 Molecular dynamics (MD) simulation, PCA, FEL and MM/GBSA analysis of 4f against 5I9B

A 100 ns MD simulation of the **4f–5I9B** complex was performed to evaluate its structural stability and interaction persistence (Fig. 7–11). The RMSD profile (Fig. 7A) showed that the complex reached equilibrium within the first 5–10 ns and remained relatively stable thereafter, with fluctuations primarily between ∼8 Å and 14 Å. Occasional spikes in the ligand RMSD were observed, suggesting conformational rearrangements in the binding pocket. However, the absence of sustained deviations indicates that the ligand remained associated with the protein during the entire simulation. In the RMSF analysis (Fig. 7B), a fluctuation in the range of ∼0.2–0.6 nm was observed, and the higher flexibility was mainly restricted to the terminal regions.

**Fig. 7:**
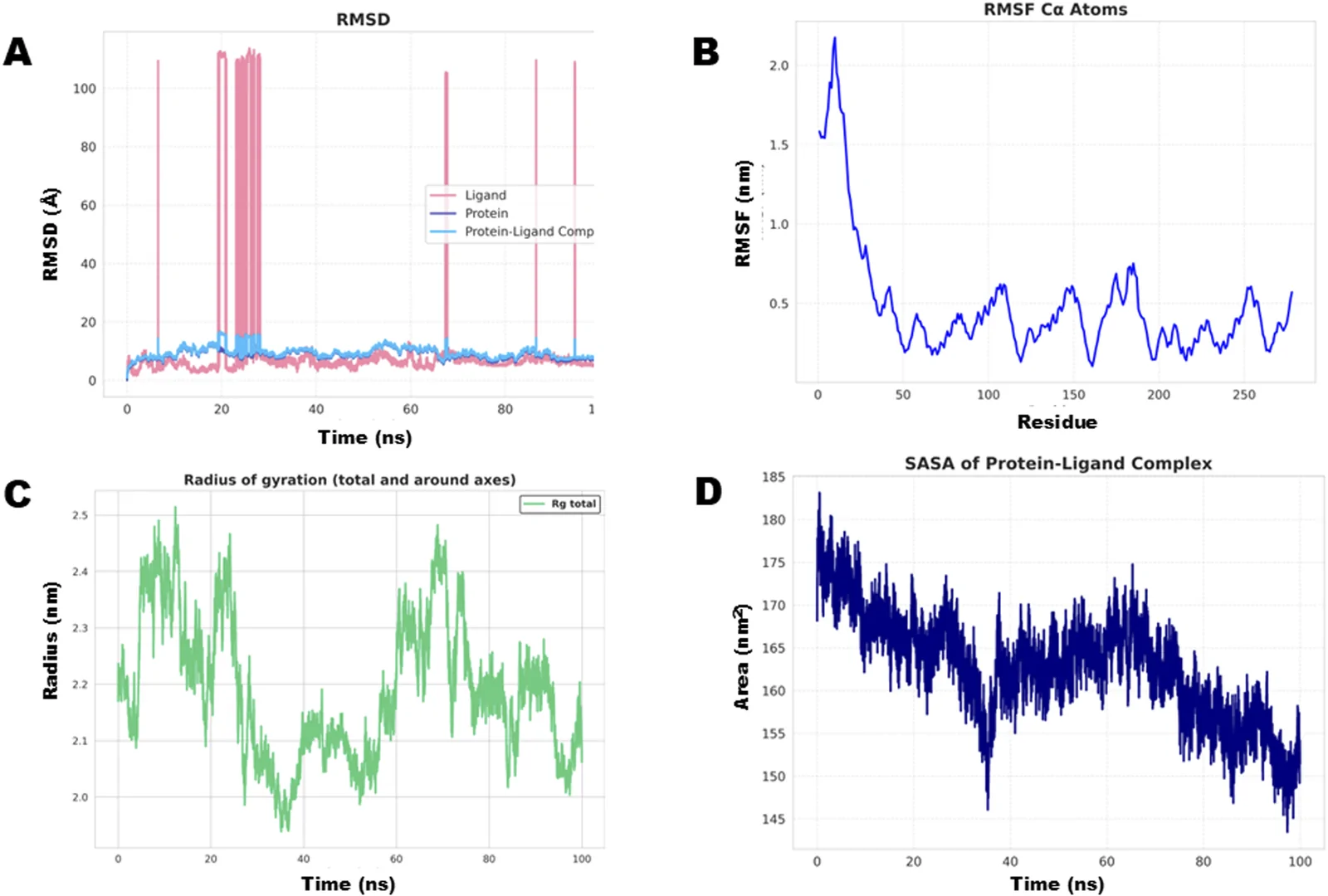
Molecular dynamics analysis of the **4f–HeLa** (5I9B) complex over 100 ns: (A) RMSD, (B) RMSF, (C) radius of gyration, and (D) SASA, indicating stability, flexibility, compactness, and solvent accessibility of the protein–ligand system.

**Fig. 8:**
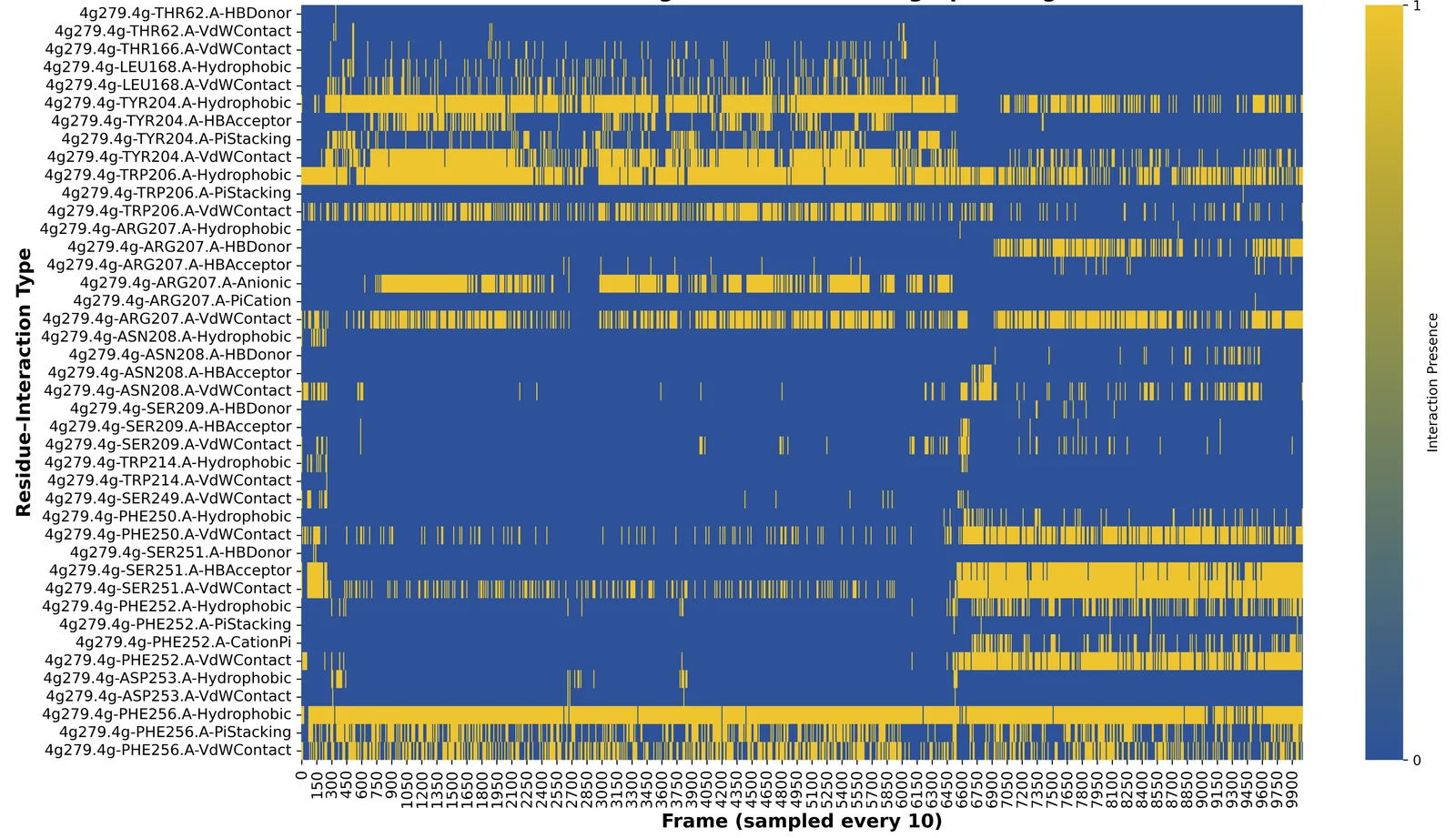
PLIF analysis of the 4f–HeLa (5I9B) complex showing interaction persistence over time, demonstrating stable protein–ligand contacts.

**Fig. 9:**
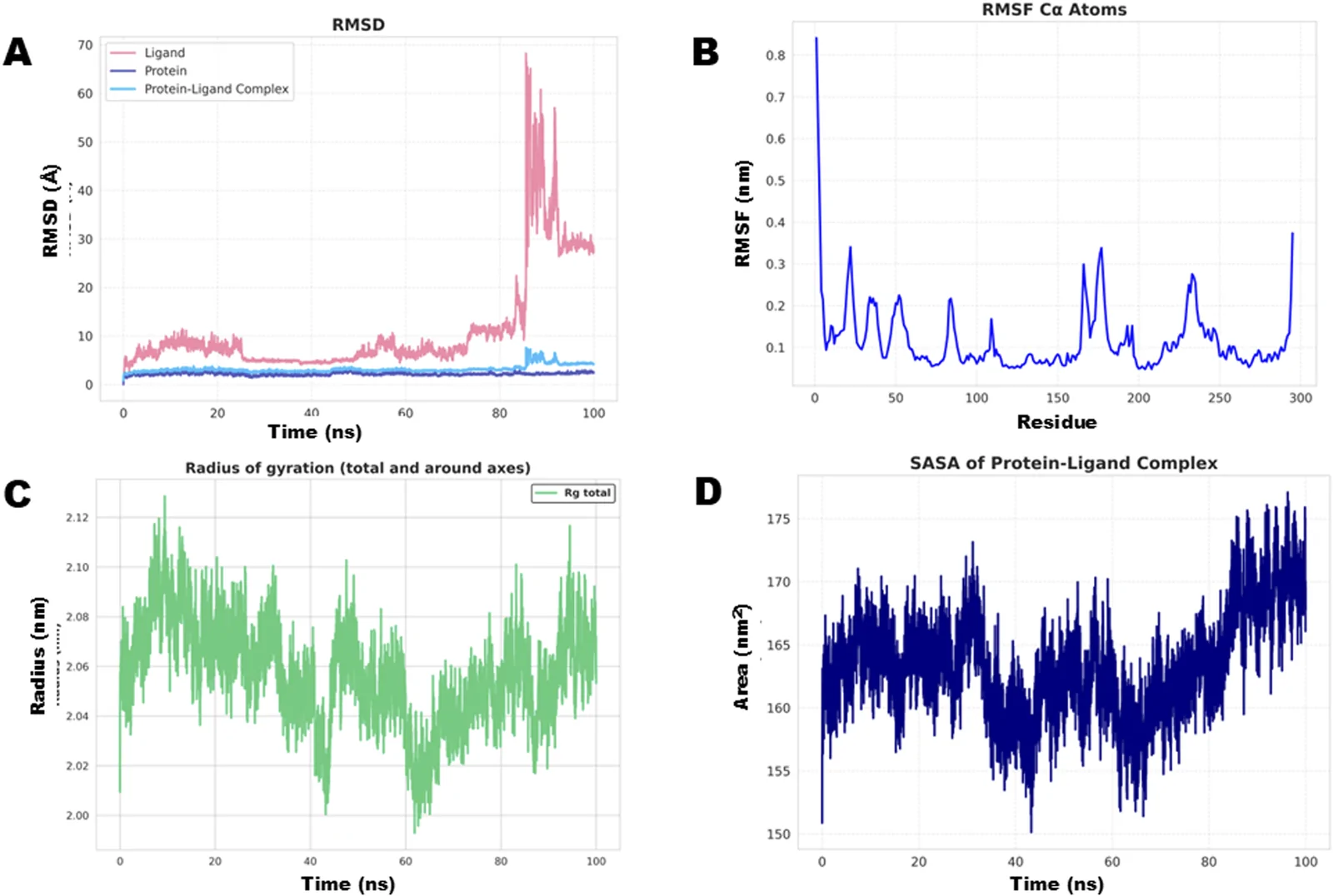
Molecular dynamics analysis of the 4f–MDA-MB231 (3KRR) complex over 100 ns: (A) RMSD, (B) RMSF, (C) radius of gyration, and (D) SASA, indicating stability, flexibility, compactness, and solvent accessibility of the protein–ligand system.

**Fig. 10:**
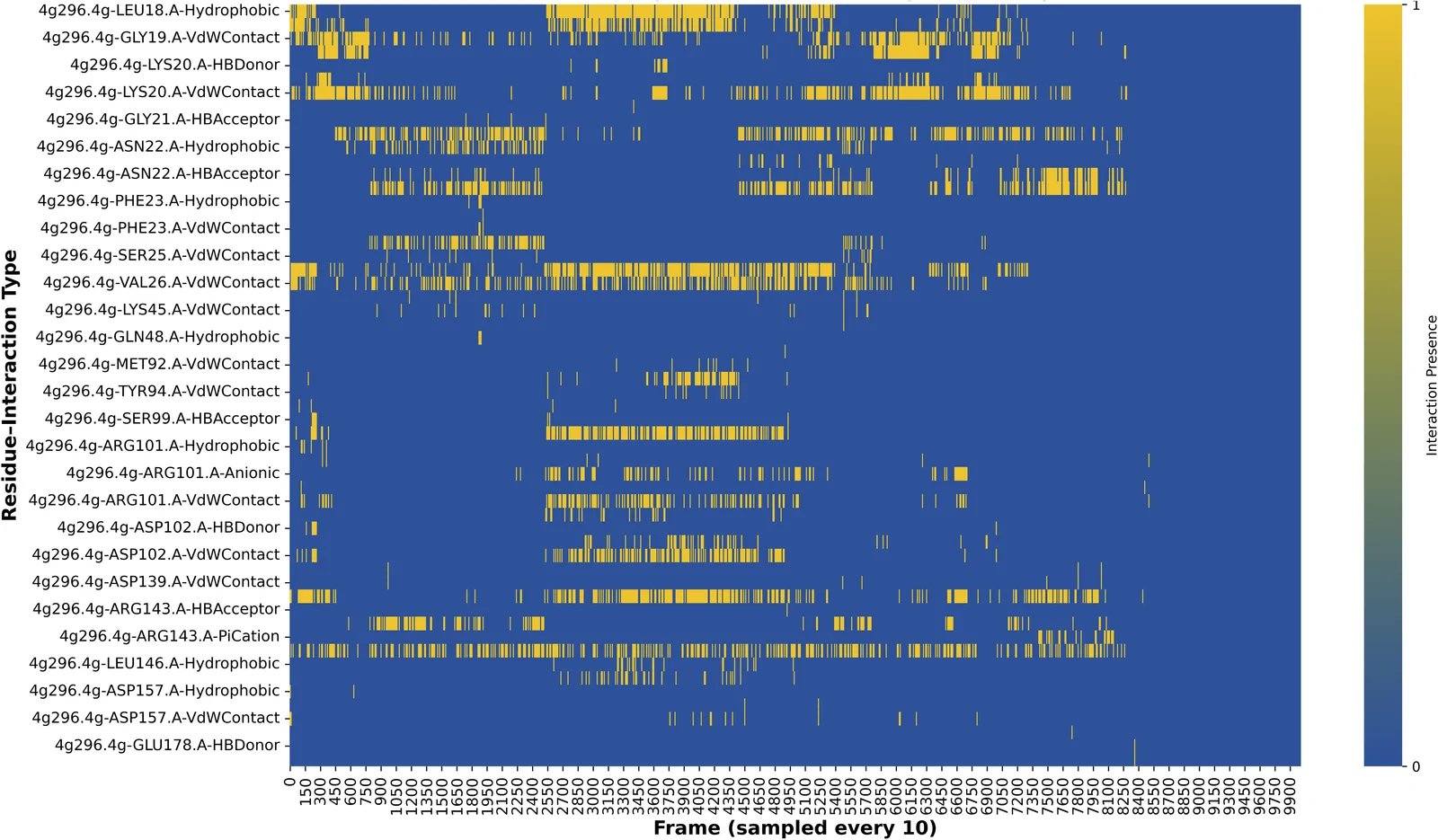
PLIF analysis of the 4f–MDA-MB231 (3KRR) complex showing interaction persistence over time, demonstrating stable protein–ligand contacts.

**Fig. 11:**
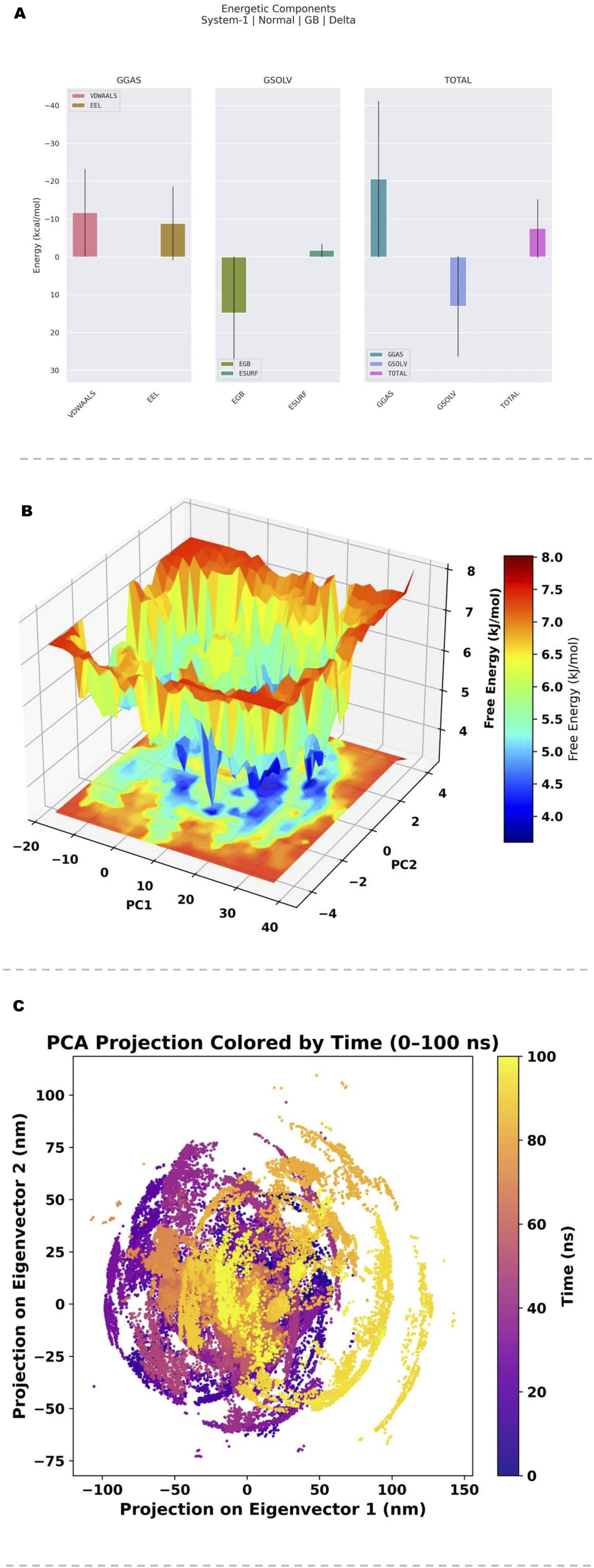
MM/GBSA, PCA, and free energy landscape analysis of the 4f–HeLa (5I9B) complex: (A) energy decomposition of binding interactions, (B) FEL depicting stab towards formational basins, and (C) time-resolved PCA projection showing conformational evolution over 100 ns.

Importantly, PHE256, TRP206, and SER251, which are key residues in the interaction, showed low fluctuations (<0.5 nm), confirming the stability of the active site throughout the simulation. The Rg profile (Fig. 7D) remained nearly constant within ∼2.0–2.45 nm, indicating the maintenance of a compact protein fold, whereas the SASA profile (Fig. 7C) gradually decreased from ∼175 to ∼150 nm^2^, suggesting improved packing of the protein–ligand complex after accommodation.

In general, PLIF analysis demonstrated various types of interactions and their persistence between the ligand and active site residues. In the case of the **4f–5I9B** (HeLa) complex (Fig. 8), hydrophobic and π–π interactions were observed with 60–80% occupancy(Fig. S38), particularly with aromatic residues such as PHE256 and TRP206. SER251 displayed consistent hydrogen-bonding interactions. The time-resolved interaction map further confirmed that these contacts were maintained throughout the trajectory, indicating that 4f formed stable and persistent interactions within the active site, which is consistent with the docking predictions.

MM/GBSA analysis of the **4g–5I9B** complex revealed a favourable binding free energy with a total Δ*G* value of −7.55 kcal mol^−1^, indicating stable ligand association within the active site. The interaction was mainly driven by van der Waals (−11.71 kcal mol^−1^) and electrostatic contributions (−8.88 kcal mol^−1^), suggesting that hydrophobic and non-covalent interactions play major roles in stabilising the complex. In contrast, the positive solvation energy (13.04 kcal mol^−1^), which largely arises from the polar solvation component (14.8 kcal mol^−1^), partially opposes the binding process. Overall, the balance between favourable gas-phase interactions and solvation effects supports the stable binding behaviour of 4f toward the 5I9B target.

Further energetic and conformational validation is presented in Fig. 11. The FEL (Fig. 11B) exhibits a broad low-energy basin (∼4–6 kcal mol^−1^) with a few shallow adjacent minima, indicating that the system predominantly resides in a stable conformational region with limited transitions between closely related states. Compared to 3KRR, the slightly wider basin suggests greater conformational adaptability while retaining overall stability. The PCA projection (Fig. 11C) showed clustering within a defined conformational space (PC1: ∼−100 to +120 nm; PC2: ∼−75 to +100 nm), with a gradual temporal dispersion across the trajectory. The colour gradient from purple (initial simulation period, ∼0 ns) to yellow (final simulation period, ∼100 ns) represents the temporal evolution of the trajectory. The substantial overlap between the early and late clusters indicates conformational convergence rather than directional drift, confirming that the complex remained within energetically favourable states while exhibiting controlled flexibility throughout the simulation.

In summary, MD, PLIF, and conformational analyses indicated that 4f maintained a stable yet dynamically adaptable binding mode within the 5I9B active site, which is consistent with its favourable docking score (−8.4 kcal mol^−1^) and higher cytotoxic activity observed in the MTT assay.

#### 2.6.2 Molecular dynamics (MD) simulation, PCA, FEL and MM/GBSA of 4f–MDA-MB231 (3KRR) complex

The dynamic stability of the **4f–3KRR** complex was evaluated using a 100 ns MD simulation (Fig. 9–12). The RMSD plot with respect to the protein–ligand complex (Fig. 9A) displayed no drastic fluctuation and was stable within ∼2.5–4.5 Å throughout the simulation, indicating overall structural stability. In contrast, the ligand RMSD increased sharply after ∼80 ns to ∼60–70 Å, suggesting substantial conformational reorientation within the binding pocket, rather than complete dissociation. Because the complex RMSD (Fig. 9A) remained relatively stable. The RMSF analysis (Fig. 9B) revealed that most residues fluctuated within ∼0.1–0.3 nm, whereas the N-terminal region displayed higher flexibility (∼0.8–0.85 nm), consistent with the intrinsic mobility of terminal segments. Importantly, relatively low fluctuations (<0.25 nm) were observed for the residues involved in ligand binding. This confirms that the active-site pocket remained stable despite ligand movement. The narrow fluctuations observed in the Rg profile (Fig. 9D), ranging from ∼2.02 to 2.10 nm, indicated that the protein retained a compact conformation throughout the simulation. Similarly, the SASA profile (Fig. 9C) varied between ∼155–170 nm^2^ and showed a slight increase during the later stages of the trajectory, reaching ∼170–178 nm^2^, suggesting minor conformational relaxation and increased solvent exposure in the later stages. Together, these observations indicate that the complex undergoes limited structural adaptation while maintaining overall conformational stability throughout the simulation period.

**Fig. 12:**
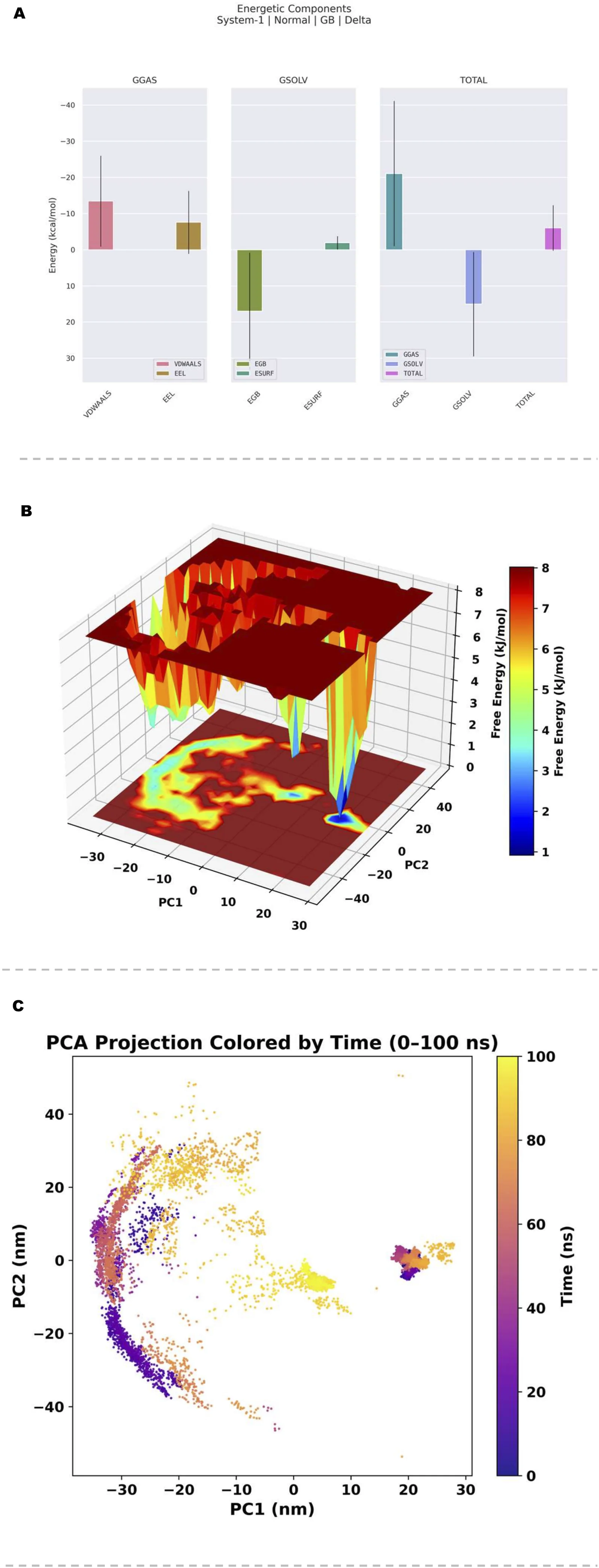
MM/GBSA, PCA, and free energy landscape analysis of the 4f–MDA-MB231(3KRR) complex: (A) energy decomposition of binding interactions, (B) FEL depicting stable conformational basins, and (C) time-resolved PCA projection showing conformational evolution over 100 ns.

The PLIF analysis (Fig. 10) of the **4f–3KRR** (MDA-MB231) complex showed that important residues such as ARG143, LEU18, GLY19, and SER99 had interaction occupancies of approximately 20–35% (Fig. S39). These are mostly van der Waals and hydrophobic contacts, with hydrogen bonding happening from time to time. The time-resolved interaction map showed that these interactions lasted for a long time, even though the ligand was flexible in some places.

Fig. 12 provides additional energetic and conformational validation. The MM/GBSA profile (Fig. 12A) shows that the gas-phase contribution (−20 kcal mol^−1^) outweighs the solvation penalty (13 kcal mol^−1^), resulting in a net favourable binding free energy of ∼−7 kcal mol^−1^, confirming thermodynamically stable binding. A well-defined low-energy basin (∼2–4 kcal mol^−1^) was observed in the FEL (Fig. 12B) indicates that the system predominantly resides in a stable conformation, with limited transitions. The PCA projection (Fig. 12C) shows that the complex remained largely confined to a defined conformational region while continuing to sample nearby conformations over time. This pattern suggests that the complex remained within a restricted conformational region, exhibiting only small thermally driven fluctuations rather than significant structural changes. In summary, MD, PLIF, and energetic analyses indicated that 4f binds dynamically and stably in the 3KRR active site, consistent with its favourable docking profile and observed biological activity. Thereafter, ADME analysis was carried out to complement the findings and evaluate the drug-likeness and pharmacokinetic suitability of the compounds (Table 7).

**Table 7:** Physicochemical properties and pharmacokinetic descriptors of the synthesised compounds (4a–4i)^a^

Code	M. Wt (g mol^−1^)	RB	HBD	HBA	TPSA (Å^2^)	log *P*	log *S* (ESOL)	GI	BBB	log *K*_p_ (cm s^−1^)	Violations	Bioavailability score	DLS
4a	328.32	1	4	1	93.18	2.00	−4.08	High	No	−6.14	0	0.56	−0.28
4b	353.33	1	5	1	116.97	1.98	−4.03	High	No	−6.5	0	0.56	−0.07
4c	362.77	1	4	1	93.18	2.24	−4.68	High	No	−5.91	0	0.56	0.41
4d	344.32	1	5	2	113.41	1.70	−3.94	High	No	−6.50	0	0.56	0.35
4e	374.35	2	6	2	122.64	2.24	−4.02	High	No	−6.69	0	0.56	0.07
4f	373.32	2	6	1	139.00	1.61	−4.15	Low	No	−6.54	0	0.56	−0.55
4g	407.22	1	4	1	93.18	2.48	−4.99	High	No	−6.14	0	0.56	0.12
4h	342.35	1	4	1	93.18	2.29	−4.38	High	No	−5.97	0	0.56	0.03
4i	346.31	1	5	1	93.18	2.24	−4.24	High	No	−6.18	0	0.56	0.33
Doxorubicin	543.52	5	12	6	206.07	2.58	−3.91	Low	No	−8.71	3	0.17	0.97
Tetracycline	444.43	2	6	9	181.62	2.02	−1.78	Low	No	−9.93	1	0.11	1.62

a Parameter definitions: M. Wt, molecular weight; RB, rotatable bonds; HBD, number of hydrogen bond donors; HBA, number of hydrogen bond acceptors; TPSA, topological polar surface area; log *P*, octanol–water partition coefficient; log *S*, solubility (ESOL); GI, gastrointestinal absorption; BBB, blood–brain barrier permeability; log *K*_p_, skin permeability; DLS, drug-likeness score.

### 2.7 ADME analysis

We used the Molsoft (https://molsoft.com/mprop/) and SwissADME (http://www.swissadme.ch/) web tools to determine the pharmacokinetic parameters of the synthesised benzo[*g*]chromene derivatives (4a–4i) (Table 8). All the compounds have the same benzo[*g*]chromene core, but the aryl ring has different substituents. This difference is evident in the behaviour of the compounds in terms of their physical and chemical properties.

**Table 8:** Optimisation of the reaction with different green solvents and conditions

Entry	Ag_2_WO_4_ (mol%)	Solvent/temperature^a^ (°C)	Time (min)	Yield^b^ (%)
1	2.5	H_2_O/RT	60	70
2	2.5	EtOH/RT	60	73
3	2.5	EtOH–H_2_O (1 : 1)/RT	50	76
4	2.5	H_2_O/100	45	78
5	2.5	EtOH/80	40	84
6	**2.5**	**EtOH**–**H**_**2**_**O (1** : **1)/70**	**20**	**95**
7	1.0	EtOH–H_2_O (1 : 1)/70	45	86
8	5.0	EtOH–H_2_O (1 : 1)/70	30	91
9	—	EtOH–H_2_O (1 : 1)/70	360	70

a Reaction conditions: 2-hydroxynaphthalene-1,4-dione (1.15 mmol), cyano-benzaldehyde (1.15 mmol), and malononitrile (1.15 mmol) were combined with different solvents and catalyst amounts at various temperatures.

b Isolated yields.

All synthesised compounds complied with Lipinski's rule of five and showed suitable molecular weights (328–407 g mol^−1^) and balanced lipophilicity (log *P* = 1.61–2.48). Compounds 4c and 4e displayed a favourable balance between polarity and lipophilicity. Although 4f showed the highest cytotoxicity, its nitro group increased the TPSA to 139 Å^2^ and reduced the predicted gastrointestinal absorption, highlighting the need for structural optimisation. In contrast, 4a combined moderate lipophilicity (log *P* = 2.00) with a lower TPSA (93.18 Å^2^), which may support bacterial membrane penetration and comparatively better antibacterial activity. Most derivatives, except 4f, showed favourable predicted gastrointestinal absorption, and none were predicted to cross the blood–brain barrier. Their skin permeability values (log *K*_p_ = −5.67 to −6.69 cm s^−1^) indicated moderate permeability.

The drug-likeness score (DLS) further distinguishes the series apart even more. Compounds 4c (0.41) and 4d (0.35) were more drug-like, whereas compound 4f, bearing the electron-withdrawing –NO_2_ group, exhibited the highest anticancer activity but had the lowest drug-likeness score (DLS = −0.55). Its comparatively high TPSA may restrict passive membrane diffusion, consequently limiting cellular permeability and oral absorption. This apparent mismatch is not unexpected because biological potency reflects target engagement, whereas DLS represents a composite estimate of physicochemical and pharmacokinetic suitability. Thus, the strong activity of 4f may arise from the favourable electronic complementarity and binding interactions contributed by the –NO_2_ substituent, despite its less favourable predicted drug-like properties.

Accordingly, 4f should be regarded as an activity-oriented lead rather than a fully optimised drug candidate. Subsequent optimisation could involve replacing the –NO_2_ group with less polar electron-withdrawing substituents or bioisosteres, such as –CN, –CF_3_, or halogens, to reduce the TPSA while retaining the favourable electron-deficient character of the scaffold.

ADME results showed that the benzo[*g*]chromene scaffold is a naturally occurring drug-like scaffold, and the variation in activity across the series can be attributed to the change in polarity and lipophilicity brought about by substituents. Compounds 4f, 4e, and 4c are promising candidates for anticancer drugs, and 4a is a potential antibacterial drug. This provides a clear path for further optimisation steps. Thus, a structure–activity relationship (SAR) analysis was performed to understand these trends.

### 2.8 Structure–activity relationship (SAR)

The biological activity of benzo[*g*]chromene derivatives 4a–4i varied with aryl substitution, indicating that electronic effects, polarity, lipophilicity, and target interactions collectively influence their activity. The common cyano- and amino-substituted scaffold provides potential hydrogen-bonding and aromatic interaction sites, while the aryl substituents modulate its physicochemical properties.

The nitro-substituted derivative 4f exhibited the highest cytotoxicity against both HeLa and MDA-MB-231 cells. Its nitro group alters the electronic distribution and increases polarity, which may favour interactions within the predicted binding pockets. Docking showed favourable binding of 4f to 5I9B and 3KRR, while MD, MM/GBSA, PCA, and FEL analyses supported dynamically plausible protein–ligand complexes. Nevertheless, the experimental activity ranking did not directly follow the HOMO–LUMO energy-gap order: 4e had the lowest energy gap, whereas 4f possessed an intermediate value. Exploratory correlation analysis likewise showed only weak-to-moderate relationships between the examined electronic descriptors and cytotoxic activity. Therefore, the higher activity of 4f cannot be attributed to a single electronic parameter.

The hydroxy- and methoxy-substituted derivatives 4d and 4e showed moderate cytotoxicity, suggesting that their hydrogen-bonding ability and electronic effects alone were insufficient to match the activity of 4f. The halogenated derivatives also showed moderate activity, indicating that increased hydrophobicity or van der Waals interactions did not independently enhance the cellular response.

The unsubstituted derivative 4a displayed the strongest preliminary antibacterial activity against *A. baumannii*. Its comparatively balanced polarity and lipophilicity may favour biological availability and interaction with the bacterial system. However, 4b produced the most favourable docking score against 7PQM, demonstrating that docking affinity alone did not reproduce the experimental antibacterial ranking. Factors such as solubility, membrane permeability, intracellular availability, and interactions with other cellular targets may also contribute.

Overall, the higher cytotoxicity of 4f probably results from the combined influence of its nitro substituent, electronic distribution, physicochemical behaviour, and predicted protein interactions. However, its elevated polarity, reduced predicted gastrointestinal absorption, and low drug-likeness score indicate the need for structural optimisation. The computational results provide complementary support for the observed activity but do not fully explain the cellular response or confirm the mechanism of action. Further target-validation and mechanistic studies are therefore required.

## 3 Conclusion

A green one-pot method was developed for synthesising benzo[*g*]chromene derivatives 4a–4i using recyclable Ag_2_WO_4_ nanoparticles, representing the first application of this catalyst to this scaffold. The protocol afforded high yields under mild aqueous-ethanol conditions. Compound 4f exhibited the highest cytotoxicity against HeLa and MDA-MB-231 cells, whereas 4a demonstrated the strongest preliminary antibacterial activity against *A. baumannii*. Computational analyses provide complementary insights into the electronic properties and predicted binding behaviour of active compounds; however, they do not establish their cellular mechanisms of action.

The study is limited by its small compound library, cell-based cytotoxicity assessment, preliminary antibacterial screening, and lack of direct target validation and *in vivo* studies. Furthermore, the comparatively low drug-likeness of 4f indicates that structural optimisation is required. Future studies should focus on expanding the derivative library, improving the physicochemical and pharmacokinetic properties of 4f, validating the proposed molecular targets, and assessing the selectivity, toxicity, mechanism of action, and therapeutic efficacy in relevant *in vitro* and *in vivo* models.

## 4 Materials and methods

### 4.1 Synthesis of 4*H*-benzo[*g*]chromenes

A mixture of substituted aromatic aldehyde (1.15 mmol), malononitrile (1.15 mmol), and 2-hydroxynaphthalene-1,4-dione (1.15 mmol) was reacted in an ethanol–water medium (1 : 1, v/v) in the presence of an Ag_2_WO_4_ nanocatalyst (2.5 mol%). The reaction mixture was stirred at 70 °C, and its progress was monitored using thin-layer chromatography (TLC). After completion, the mixture was allowed to cool to room temperature, and the precipitated solid was isolated *via* filtration. The crude product was washed with ethanol and subsequently dissolved in a minimum amount of DMSO. The Ag_2_WO_4_ nanocatalyst was recovered by centrifugation at 10 000 rpm for 10 min, washed two to three times with ethanol, and dried under vacuum at 70 °C prior to reuse. The filtrate was added dropwise to cold water to obtain a precipitate. Final purification was achieved by recrystallisation from ethanol to afford pure 4*H*-benzo[*g*]chromene derivatives (4a–4i).

### 4.2 Catalyst recyclability

For the catalyst recyclability study, the model reaction was performed on a tenfold scale using substituted aromatic aldehydes, malononitrile, and 2-hydroxynaphthalene-1,4-dione (11.5 mmol each) under the optimised reaction conditions. An Ag_2_WO_4_ nanocatalyst (2.5 mol%, 0.2875 mmol, 133 mg) was employed to facilitate reliable recovery and reuse. After each cycle, the catalyst was recovered by centrifugation, washed with ethanol, dried under vacuum at 70 °C, and reused in the next cycle.

### 4.3 Preparation of Ag_2_WO_4_ catalyst

The Ag_2_WO_4_ nanocatalyst used in this study was prepared and characterised as reported previously.^28^ In short, stoichiometric aqueous solutions of silver nitrate (AgNO_3_, 10 mmol) and sodium tungstate dihydrate (Na_2_WO_4_·2H_2_O, 5 mmol) were prepared. To form precipitates, the AgNO_3_ solution was gradually added to the Na_2_WO_4_ solution while stirring continuously. Subsequently, the mixture was placed in a 100 mL Teflon-lined stainless-steel autoclave and hydrothermally treated for 16 h at 180 °C. The product was collected by centrifugation, washed with ethanol and deionised water, and dried overnight at 60 °C after being cooled to room temperature.

### 4.4 Optimization of reaction

A model reaction of *p*-cyanobenzaldehyde, malononitrile, and 2-hydroxynaphthalene-1,4-dione (Scheme 1) was performed under different conditions, using eco-friendly solvents and Ag_2_WO_4_ nanocatalysts. The optimisation results are presented in Table 8. At room temperature, moderate yields were obtained in water (70%) and ethanol (73%) within 60 min of reaction. A mixed solvent system consisting of EtOH and H_2_O in a 1 : 1 ratio resulted in a slightly enhanced yield of 76%. Increasing the temperature enhanced the reaction efficiency, and the yields improved to 78% in water at 100 °C and 84% in ethanol at 80 °C with reduced reaction times. A notable improvement was observed in EtOH–H_2_O (1 : 1) at 70 °C, where the product was obtained with a 95% yield within 20 min.

The effect of catalyst loading was examined under these conditions. Increasing the amount of Ag_2_WO_4_ to 5.0 mol% did not provide any further benefit, whereas reducing it to 1.0 mol% caused a small reduction in the yield (86%) and a longer reaction time. Based on these observations, 2.5 mol% Ag_2_WO_4_ in EtOH–H_2_O (1 : 1) at 70 °C was considered optimal for this transformation.

### 4.5 General experimental procedures

The synthesised 4*H*-benzo[*g*]chromene derivatives were characterised using standard spectroscopic methods. FTIR spectra were recorded on a PerkinElmer Spectrum Two instrument equipped with an ATR accessory (diamond crystal, DTGS detector) over the range of 4000–400 cm^−1^ at a resolution of 4 cm^−1^. The spectra were acquired in the ATR mode without further sample preparation. ^1^H and ^13^C NMR spectra were obtained in DMSO-d_6_ using a Bruker Avance III 400 MHz spectrometer.

### 4.6 Characterisation data

#### 4.6.1 2-Amino-5,10-dioxo-4-phenyl-5,10-dihydro-4*H*-benzo[*g*]chromene-3-carbonitrile (4a)

Yellowish brown solid; mp 259–261 °C; ^1^H NMR (400 MHz, DMSO-d_6_): *δ* = 8.56 (1H, s), 7.93–6.97 (10H, m), 4.65 (1H, s) ppm; ^13^C NMR (100 MHz, DMSO-d_6_): *δ* = 196.77, 188.49, 165.95, 159.51, 157.85, 152.80, 142.02, 136.48, 131.52, 129.12, 127.87, 126.42, 112.44, 57.84, 35.34 ppm; FT-IR (*ν*, cm^−1^): 3400, 3322 (N–H stretching), 3193 (aromatic C–H), 3023 (C–H stretching), 2199 (C≡N), 1668 (C=O), 1593 (C=C, aromatic), 1245 (C–O–C), 1071 (C–O), 787, 719 (aromatic C–H bending); HRMS (ESI) *m*/*z* [M + H]^+^ calcd for C_20_H_13_N_2_O_3_^+^: 329.0921 found: 329.0929.

#### 4.6.2 2-Amino-4-(4-cyanophenyl)-5,10-dioxo-5,10-dihydro-4*H*-benzo[*g*]chromene-3-carbonitrile (4b)

Orange solid; mp 279–281 °C; ^1^H NMR (400 MHz, DMSO-d_6_): *δ* = 8.14–7.94 (1H, m), 7.90–7.67 (5H, m), 7.63–7.29 (4H, m), 4.74 (1H, s) ppm; ^13^C NMR (100 MHz, DMSO-d_6_): *δ* = 182.55, 176.74, 158.40, 149.46, 149.05, 134.54, 134.22, 132.59, 130.94, 130.71, 128.88, 126.09, 125.83, 120.69, 119.07, 118.74, 109.93, 56.54, 36.68 ppm; FT-IR (*ν*, cm^−1^): 3436, 3331 (N–H stretching), 3191, 3061 (aromatic C–H stretching), 2233, 2197 (C≡N), 1663 (C=O), 1595, 1506 (aromatic C=C), 1239, 1073 (C–O–C), 839, 779, 716 (aromatic C–H bending); HRMS (ESI) *m*/*z* [M + H]^+^ calcd for C_21_H_12_N_3_O_3_^+^: 354.0873 found: 354.0875.

#### 4.6.3 2-Amino-4-(4-chlorophenyl)-5,10-dioxo-5,10-dihydro-4*H*-benzo[*g*]chromene-3-carbonitrile (4c)

Dark brown solid; mp 243–245 °C; ^1^H NMR (400 MHz, DMSO-d_6_): *δ* = 8.55 (1H, s), 8.32–7.75 (5H, m), 7.77–7.65 (1H, m), 7.38 (3H, d, J 5.1), 4.65 (1H, s) ppm; ^13^C NMR (100 MHz, DMSO-d_6_): *δ* = 183.02, 177.28, 160.59, 158.79, 149.53, 143.10, 139.50, 134.99, 134.63, 132.61, 132.16, 131.45, 131.12, 130.56, 130.20, 130.15, 128.97, 126.52, 126.27, 121.82, 119.67, 114.54, 113.50, 82.74, 57.52, 36.45 ppm; FT-IR (*ν*, cm^−1^): 3409, 3332 (N–H stretching), 3193 (aromatic C–H), 2206 (C≡N), 1658 (C=O), 1578, 1490 (aromatic C=C), 1301, 1244, 1040 (C–O–C/C–O), 872, 827, 715 (aromatic C–H bending), 615 (C–Cl); HRMS (ESI) *m*/*z* [M + H]^+^ calcd for C_20_H_12_ClN_2_O^+^: 363.0531 found: 363.0536.

#### 4.6.4 2-Amino-4-(4-hydroxyphenyl)-5,10-dioxo-5,10-dihydro-4*H*-benzo[*g*]chromene-3-carbonitrile (4d)

Dark brown solid; mp 255–257 °C; ^1^H NMR (400 MHz, DMSO-d_6_): *δ* = 8.55 (1H, s), 8.13–7.99 (1H, m), 7.97–7.74 (4H, m), 7.60–7.14 (5H, m), 4.64 (1H, s) ppm; ^13^C NMR (100 MHz, DMSO-d_6_): *δ* = 192.66, 182.57, 165.02, 160.31, 158.30, 149.09, 143.07, 134.17, 132.70, 131.41, 130.99, 130.05, 128.31, 126.06, 125.80, 120.21, 56.94, 38.90 ppm; FT-IR (*ν*, cm^−1^): 3400, 3334 (N–H/O–H stretching), 3197 (aromatic C–H), 2207 (C≡N), 1645 (C=O), 1592, 1515 (aromatic C=C), 1366, 1207 (C–O–C/C–O), 715 (aromatic C–H bending); HRMS (ESI) *m*/*z* [M + H]^+^ calcd for C_20_H_13_N_2_O_4_^+^: 345.0870 found: 345.0873.

#### 4.6.5 2-Amino-4-(3-hydroxy-4-methoxyphenyl)-5,10-dioxo-5,10-dihydro-4*H*-benzo[*g*]chromene-3-carbonitrile (4e)

Dark brown solid; mp 260–262 °C; ^1^H NMR (400 MHz, DMSO-d_6_): *δ* = 9.85 (1H, s), 8.30 (2H, s), 8.09–7.61 (3H, m), 7.61–7.30 (3H, m), 7.17 (2H, d, J 8.5), 3.90 (3H, s) ppm; ^13^C NMR (100 MHz, DMSO-d_6_): *δ* = 160.70, 153.86, 146.98, 126.39, 124.35, 115.28, 114.99, 113.93, 112.20, 76.22, 55.98 ppm; FT-IR (*ν*, cm^−1^): 3388 (O–H/N–H stretching), 3027 (aromatic C–H), 2229 (C≡N), 1642 (C=O), 1562, 1524 (aromatic C=C), 1342, 1280, 1137 (C–O–C/C–O), 1012 (C–O, methoxy), 870, 760 (aromatic C–H bending); HRMS (ESI) *m*/*z* [M + H]^+^ calcd for C_21_H_15_N_2_O_5_^+^: 375.0975 found: 375.0978.

#### 4.6.6 2-Amino-4-(2-nitrophenyl)-5,10-dioxo-5,10-dihydro-4*H*-benzo[*g*]chromene-3-carbonitrile (4f)

Orange solid; mp 243–245 °C; ^1^H NMR (400 MHz, DMSO-d_6_): *δ* = 8.26–7.78 (5H, m), 7.74–7.53 (2H, m), 7.53–7.37 (3H, m), 5.40 (1H, s) ppm; ^13^C NMR (100 MHz, DMSO-d_6_): *δ* = 183.16, 177.23, 159.37, 149.51, 149.02, 138.32, 135.07, 134.76, 134.29, 131.83, 131.23, 131.10, 128.89, 126.57, 126.31, 124.54, 121.63, 119.19, 56.21, 31.51 ppm; FT-IR (*ν*, cm^−1^): 3392, 3335 (N–H stretching), 3028 (aromatic C–H), 2230 (C≡N), 1666 (C=O), 1590, 1525 (aromatic C=C/NO_2_ asymmetric), 1360 (NO_2_ symmetric), 1250, 1136, 1013 (C–O–C/C–O), 857, 785, 714 (aromatic C–H bending); HRMS (ESI) *m*/*z* [M + H]^+^ calcd for C_20_H_12_N_3_O_5_^+^: 374.0771 found: 374.0776.

#### 4.6.7 2-Amino-4-(4-bromophenyl)-5,10-dioxo-5,10-dihydro-4*H*-benzo[*g*]chromene-3-carbonitrile (4g)

Dark brown solid; mp 253–255 °C; ^1^H NMR (400 MHz, DMSO-d_6_): *δ* = 8.05 (2H, dt, *J* 4.9, 3.1), 8.00–7.77 (3H, m), 7.63–7.11 (5H, m), 5.65 (1H, s) ppm; ^13^C NMR (100 MHz, DMSO-d_6_): *δ* = 182.92, 177.18, 159.69, 150.22, 136.13, 135.39, 134.84, 131.17, 130.79, 130.62, 130.18, 126.68, 126.44, 120.60, 118.97, 53.33, 33.94 ppm; FT-IR (*ν*, cm^−1^): 3405, 3331 (N–H stretching), 3106 (aromatic C–H), 2205 (C≡N), 1668 (C=O), 1593, 1488 (aromatic C=C), 1365, 1244, 1073, 1010 (C–O–C/C–O), 828, 770, 714 (aromatic C–H bending), 614 (C–Br); HRMS (ESI) *m*/*z* [M + H]^+^ calcd for C_20_H_12_BrN_2_O_3_^+^: 408.0026 found: 408.0032.

#### 4.6.8 2-Amino-5,10-dioxo-4-(*p*-tolyl)-5,10-dihydro-4*H*-benzo[*g*]chromene-3-carbonitrile (4h)

Dark brown solid; mp 240–242 °C; ^1^H NMR (400 MHz, DMSO-d_6_): *δ* = 8.19–8.01 (1H, m), 7.87 (3H, m), 7.43–7.14 (6H, m), 4.62 (1H, s), 2.53 (3H, s) ppm; ^13^C NMR (100 MHz, DMSO-d_6_): *δ* = 183.05, 177.35, 168.37, 158.82, 149.43, 144.08, 135.01, 134.63, 131.49, 129.06, 128.15, 127.55, 126.55, 126.28, 122.42, 57.91, 36.95 ppm; FT-IR (*ν*, cm^−1^): 3322 (N–H stretching), 3194 (aromatic C–H), 2201 (C≡N), 1668 (C=O), 1594, 1364 (aromatic C=C), 1245, 1076 (C–O–C/C–O), 715 (aromatic C–H bending); HRMS (ESI) *m*/*z* [M + H]^+^ calcd for C_21_H_15_N_2_O_3_^+^: 343.1077 found: 343.1085.

#### 4.6.9 2-Amino-4-(4-fluorophenyl)-5,10-dioxo-5,10-dihydro-4*H*-benzo[*g*]chromene-3-carbonitrile (4i)

Dark brown solid; mp 287–289 °C; ^1^H NMR (400 MHz, DMSO-d_6_): *δ* = 8.26–7.99 (1H, m), 7.93–7.68 (5H, m), 7.66–7.33 (4H, m), 4.75 (1H, s) ppm; ^13^C NMR (100 MHz, DMSO-d_6_): *δ* = 183.03, 177.21, 158.83, 149.95, 149.54, 134.99, 134.68, 133.04, 131.42, 131.21, 129.33, 126.55, 126.28, 121.10, 119.52, 119.19, 110.36, 56.95, 37.10 ppm; FT-IR (*ν*, cm^−1^): 3323 (N–H stretching), 3194 (aromatic C–H), 2201 (C≡N), 1667 (C=O), 1594, 1509 (aromatic C=C), 1362, 1205, 1075 (C–O–C/C–O), 950 (C–F), 716 (aromatic C–H bending), 507 (C–F); HRMS (ESI) *m*/*z* [M + H]^+^ calcd for C_20_H_12_FN_2_O_3_^+^: 347.0826 found: 347.0835.

### 4.7 Computational studies

#### 4.7.1 Density functional theory (DFT) calculations

Avogadro software was used to generate the initial geometries of the 4*H*-benzo[*g*]chromene derivatives (4a–4i).^32^ We used the B3LYP/def2-TZVP method in ORCA 6.0.1 to perform Density Functional Theory (DFT) computations.^33,34^ This method uses a def2/J auxiliary basis set for the resolution of the identity (RI) approximation.^35–38^ Koopmans' theorem was used to determine the electronic structural features and global reactivity descriptors.^39^ We used NBO 7.0 and ORCA to perform Natural Bond Orbital (NBO) analysis to investigate donor–acceptor interactions and measure second-order perturbation energies.^40,41^ All NBO computations were conducted at the B3LYP/def2-TZVP level of theory.^42^ Chemcraft was used to create molecular visualisations, such as HOMO–LUMO surfaces and Molecular Electrostatic Potential (MEP) maps.^43^

#### 4.7.2 Molecular docking

The binding affinity and interaction profile of the synthesised ligands (4a–4i) with particular biological targets pertinent to the experimental investigations were examined using molecular docking analysis. For the anticancer studies, the human caspase-3 receptor (PDB ID: 5I9B) was considered in relation to HeLa cell line activity, whereas Janus kinase 2 (JAK2) (PDB ID: 3KRR) was selected for the MDA-MB231 breast cancer model. In addition, DNA gyrase B from *Acinetobacter baumannii* (PDB ID: 7PQM) was employed as the target protein to support antimicrobial (MIC) studies.

The crystal structures of the proteins were retrieved from the RCSB Protein Data Bank at resolutions of 2.10 Å (5I9B), 1.80 Å (3KRR), and 1.55 Å (7PQM). Before docking, the protein structures were prepared using AutoDock Tools 1.5.7. This involved removing the co-crystallised ligands and water molecules, adding polar hydrogen atoms, and assigning the correct charges. The prepared protein structures were used for subsequent docking analyses.

Ligand geometries were optimised at the B3LYP/def2-TZVP level using ORCA 6.0.1, and the resulting structures were converted into 3D formats for docking after assignment of Gasteiger charges. The prepared ligands were saved in PDBQT format and docked using AutoDock Vina.^44^ For each target protein (5I9B, 3KRR, and 7PQM), the grid box was centred on the active site to adequately encompass the key binding residues. The protein–ligand complexes were further analysed using Discovery Studio Visualiser^45^ and UCSF ChimeraX^46^ to evaluate the binding orientation, hydrophobic interactions, and hydrogen bonding within the active site.

#### 4.7.3 Molecular dynamic simulation

Molecular dynamics simulations were conducted using GROMACS 2025.4 with GPU support in the Google Colab Pro environment.^47^ The protein–ligand system was parameterised using the CHARMM36m force field, with ligand parameters generated *via* the CGenFF server and adapted for GROMACS.^48^ The complex was solvated in a triclinic TIP3P water box and neutralised with Na^+^ and Cl^−^ ions.^49^ After energy minimisation using the steepest descent method, the system was equilibrated under NVT and NPT conditions (298 K, 1 atm) using the V-rescale thermostat and Parrinello–Rahman barostat.^50,51^ A 100 ns production simulation was subsequently performed under periodic boundary conditions. We performed trajectory analyses using GROMACS tools and complementary Python packages. Conformational dynamics were evaluated using principal component analysis (PCA) and free energy landscape (FEL) calculations based on backbone covariance. The binding free energies were calculated using the MM/GBSA method implemented in the gmx_MMPBSA.^52^

#### 4.7.4 Pharmacokinetics and drug-likeness prediction

The SwissADME online server (https://www.swissadme.ch/)^53^ and the MolSoft drug-likeness calculator (https://molsoft.com/mprop/)^54^ were used to assess the pharmacokinetics and drug-likeness of the synthesised compounds (4a–4i). SwissADME was used to predict the key pharmacokinetic parameters, and MolSoft was used to estimate the drug-likeness scores. These *in silico* assessments provide preliminary indications of the oral druggability and pharmacokinetic potential of the synthesised molecules.

### 4.8 MTT assay

Cells procured from the NCCS (Pune, India) were maintained in DMEM supplemented with 10% FBS and antibiotics under standard culture conditions (37 °C, 5% CO_2_). Cells were seeded into 96-well plates at a density of 5 × 10^3^ cells per well and allowed to adhere overnight. Subsequently, the cells were exposed to compounds 4a–4i (0.1–100 µM) for 24 and 48 h. Untreated cells served as the control, with 0.2% DMSO as the vehicle control and doxorubicin as the reference. Following treatment, MTT reagent (5 mg mL^−1^) was added and incubated for 3–4 h to allow for formazan formation, which was then solubilised in DMSO. Absorbance was recorded at 540 nm, and cell viability was calculated as a percentage relative to that of the control (mean ± SE, *n* = 3). Additional experimental details are provided in the SI (Section S3).^55,56^

## Author contributions

Sathiaseelan Perumal: writing – original draft, methodology, visualisation, software, investigation, formal analysis, and data curation. Eswar Rao Tatta: investigation, formal analysis. Kiran Kumar Nalla: investigation. Perumal Muthuraja: resources, writing – review & editing. Venkatesan Muthukumar: resources. Manisankar Paramasivam: project administration and resources, conceptualization. Viswanathan Subramanian: project administration and resources, writing – review & editing, and supervision.

## Conflicts of interest

There are no conflicts of interest to declare.

## Supplementary Material

RA-016-D6RA05223A-s001

## Data Availability

All data generated or analysed during this study are included in this published article and its supplementary information (SI). Supplementary information is available. See DOI: https://doi.org/10.1039/d6ra05223a.
