# Supplementary material for: Nanocatalysed green synthesis of benzo[g]chromene derivatives: DFT, docking, molecular dynamics and biological evaluation
Source: RSC Adv. 2026 Aug 18;16(43):52422–41. doi: 10.1039/d6ra05223a (PMC13484244; doi:10.1039/d6ra05223a)
Supplement: Supplementary file 1 [file RA-016-D6RA05223A-s001.pdf]

Supplementary Information (SI) for RSC Advances.
This journal is © The Royal Society of Chemistry 2026

Nanocatalysed Green Synthesis of Benzo[g]chromene Derivatives: DFT, Docking, Molecular Dynamics and Biological Evaluation

Sathiaseelan Perumal [a, b], Eswar Rao Tatta [c], Kiran Kumar Nalla [d], Perumal Muthuraja [e], Venkatesan Muthukumar [f], Paramasivam Manisankar [a*], Viswanathan Subramanian [a*]

[a] Department of Industrial Chemistry, Alagappa University, Karaikudi, Tamil Nadu 630 006, India.

[b] Department of Chemistry, Bishop Heber College, Tiruchirappalli, Tamil Nadu 620 017, India.

[c] School of Life Sciences, Department of Biotechnology, Pondicherry University, Puducherry-605014 India

[d] School of Life Sciences, University of Hyderabad, Hyderabad-500046, Telangana, India.

[e] Department of Chemistry, Indian Institute of Technology Guwahati, Guwahati-781 039, India.

[f] Department of Chemistry, School of Advanced Sciences, VIT, Vellore, Tamil Nadu 632014, India.

*Corresponding Authors: Viswanathan Subramanian, Paramasivam Manisankar

E-mail addresses:

viswanathans@alagappauniversity.ac.in (V.S), manisankarp@alagappauniversity.ac.in (P.M)

Table of Contents – Supplementary Information

Section S1. NMR and IR Spectra

Fig. S1–S27: NMR (^{1}H, ^{13}C) and IR spectra of compound 4a-4i

Section S2. Molecular Docking

Fig. S28 Two-dimensional protein–ligand interaction diagrams of benzo[g]chromene derivatives (A–I) within the active site of the *Acinetobacter baumannii* target (PDB: 7PQM).

Fig. S29 Two-dimensional protein–ligand interaction diagrams of benzo[g]chromene derivatives (A–I) within the active site of the HeLa-associated protein (PDB: 5I9B).

Fig. S30 Two-dimensional protein–ligand interaction diagrams of benzo[g]chromene derivatives (A–I) within the active site of the MDA-MB231-associated target protein (PDB: 3KRR).

Section S3. MTT Assay

3.1 Maintenance and sub-culturing of cell lines

3.2 Cell count:

3.3 Drug treatments and MTT assay for compounds

Fig. S31: Cell viability graph of 4d compound for MDA-MB231, HeLa and Hek293 cells for 24 and 48 hrs

Fig. S32: Cell viability graph of 4f compound for MDA-MB231, HeLa and Hek293 cells for 24 and 48 hrs

Section S4. DFT

Fig. S33: (HOMO, LUMO) orbitals, Molecular Electrostatic Potential plots (MEP) and optimized structures (4a–4i, doxorubicin and tetracycline) at B3LYP/def2-TZVP level in gas phase.

Table S1. Exploratory Spearman rank correlations between the calculated electronic descriptors and 48 h cytotoxic activity of compounds **4a–4i**.

Section S5. Characterisation of Ag_2WO_4 nanocatalyst

S5.1 X-ray diffraction analysis

Fig.S34**:** X-ray diffraction (XRD) pattern of synthesized Ag_2WO_4 nanocatalyst, showing characteristic diffraction peaks

S5.2 SEM, EDX, and elemental-mapping analyses

Fig. S35: SEM images of Ag_2WO_4 at different magnifications: (a, b) low magnification showing agglomerated particle clusters, and (c, d) higher magnification revealing fused rod-like structures with uneven growth and rough surfaces. (e) EDAX spectrum confirming the presence of Ag, W, and O without any impurities. Elemental mapping images (Ag, W, O).

S5.3 XPS Analysis of Ag_4WO_4

Fig. S36: X-ray photoelectron spectroscopy (XPS) analysis of the Ag_2WO_4 nanoparticles: high-resolution deconvoluted spectra of (A) O 1s, (B) Ag 3d, and (C) W 4f regions, and (D) the wide-range survey spectrum confirming the presence of Ag, W, and O elements. The experimental data, fitted envelope, and individual fitted components are shown in the respective high-resolution spectra.

S5.4 Investigation of Reusability and Recyclability of Ag_2WO_4

Fig. S37. Reusability of the Ag_2WO_4 nanocatalyst over five consecutive reaction cycles under the optimized reaction conditions.

Table S2. Comparison of the catalytic performance, reaction conditions, product yields, recyclability, and turnover parameters of Ag_2WO_4 nanoparticles with previously reported catalytic systems for the synthesis of benzo[g]chromene derivatives.

S5.4 Calculation of green chemistry metrics

Table S3. Material inventory

Section S6. NBO Analysis

Table S4 Selected second-order perturbation theory interactions ($\Delta E > 20.0$ kcal/mol) from the Fock matrix in the NBO basis for compound **4f** showed significant donor–acceptor delocalisation, contributing to electronic stabilisation.

Section S7. MD Simulation

Fig. S38. Protein–ligand interaction fingerprint (PLIF) occupancy profile of compound 4f with the HeLa-associated target 5I9B during the molecular dynamics simulation. The plot shows the percentage occupancy of the principal hydrophobic, van der Waals, hydrogen-bonding, electrostatic, and aromatic interactions.

Fig. S39. Protein–ligand interaction fingerprint (PLIF) occupancy profile of compound 4f with the MDA-MB231-associated target 3KRR during the molecular dynamics simulation. The plot shows the percentage occupancy of the principal hydrophobic, van der Waals, hydrogen-bonding, electrostatic, and aromatic interactions.

Section S1. NMR and IR Spectra

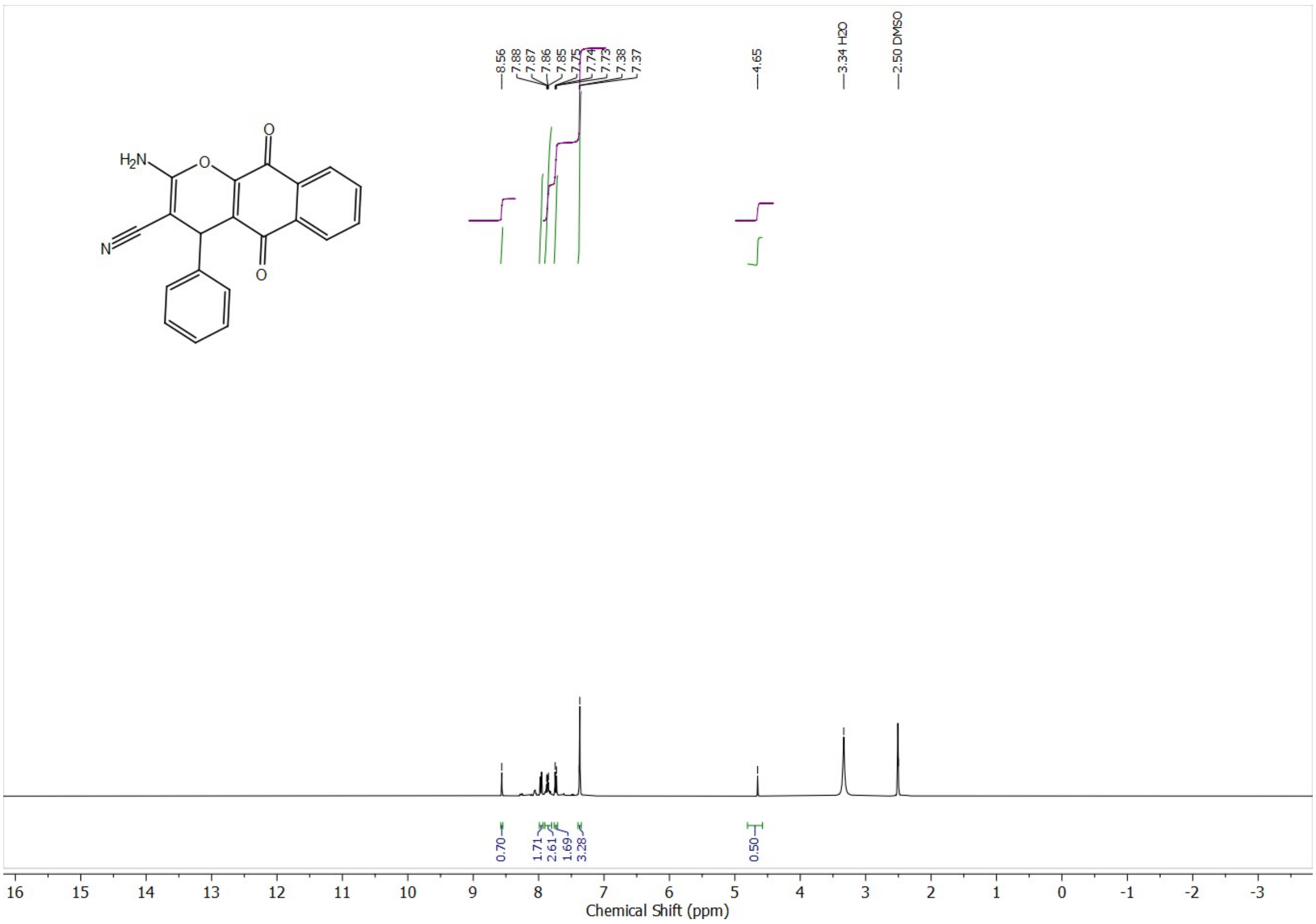

Fig. S1. ^{1}H NMR spectrum of **compound 4a** recorded in DMSO-d$_6$ (400 MHz).

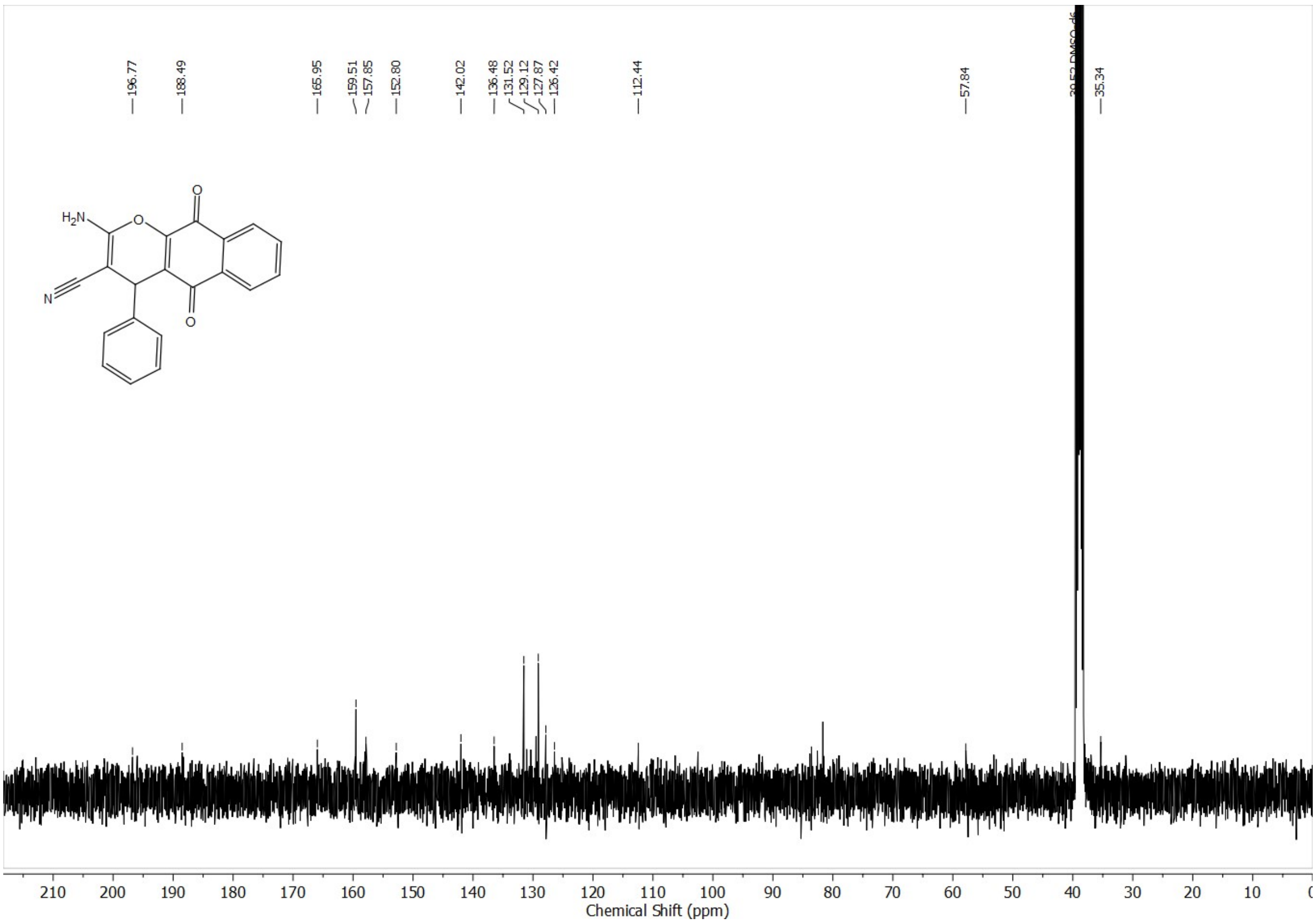

Fig. S2. ^{13}C NMR spectrum of **compound 4a** in DMSO-d_6 (101 MHz).

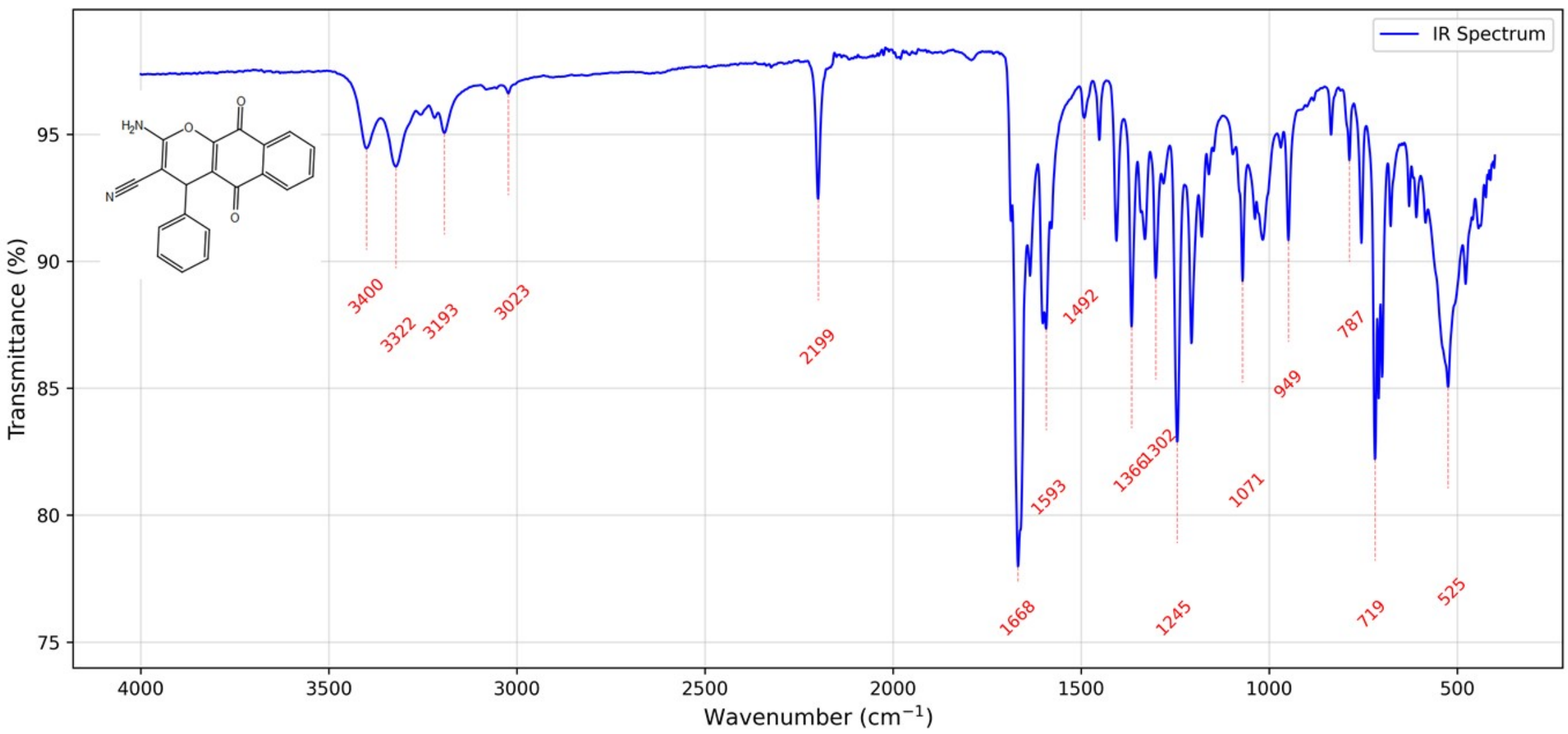

Fig. S3. IR spectrum of **compound 4a** (FT-IR).

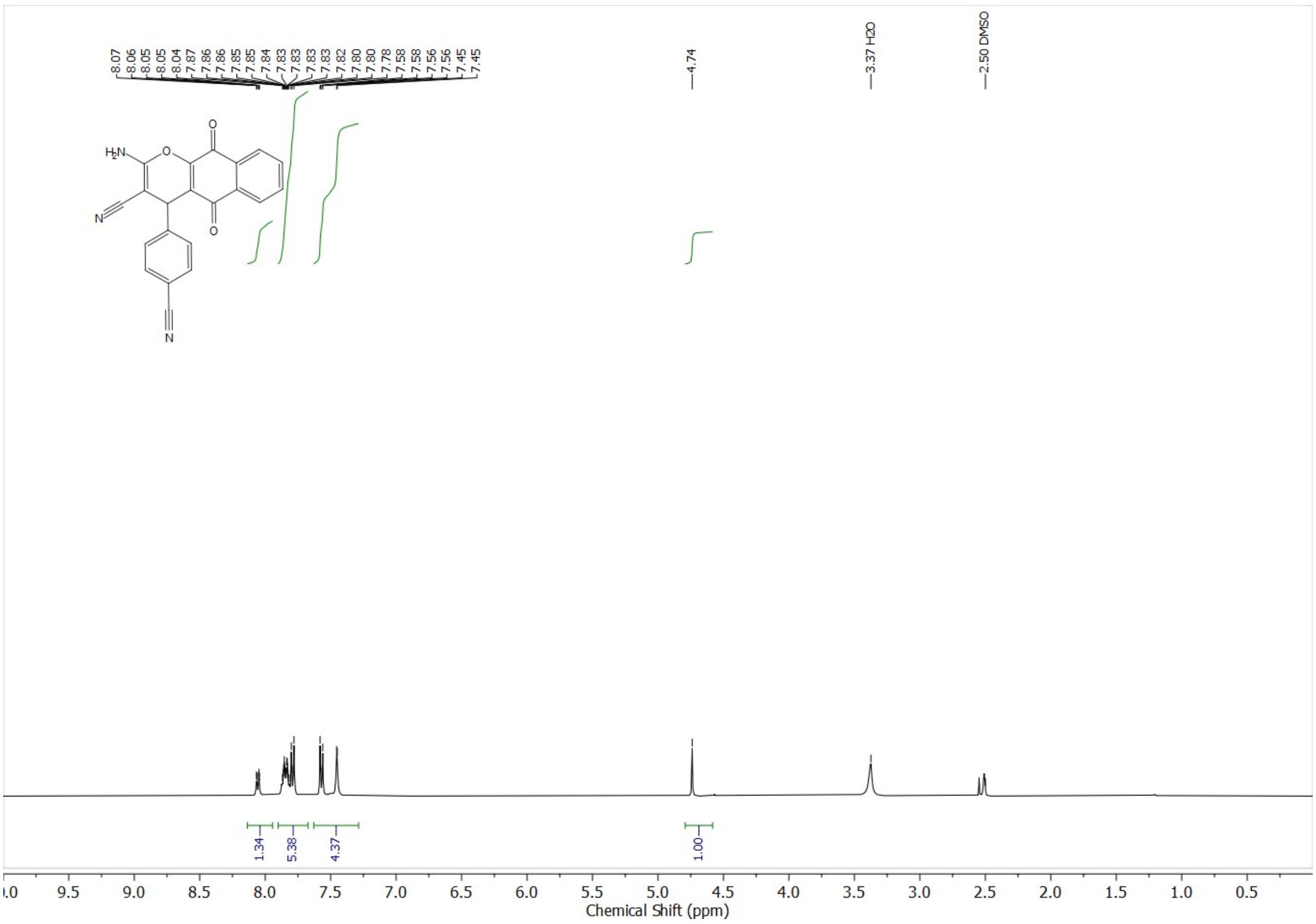

Fig. S4. ^{1}H NMR spectrum of **compound 4b** recorded in DMSO-d_6 (400 MHz).

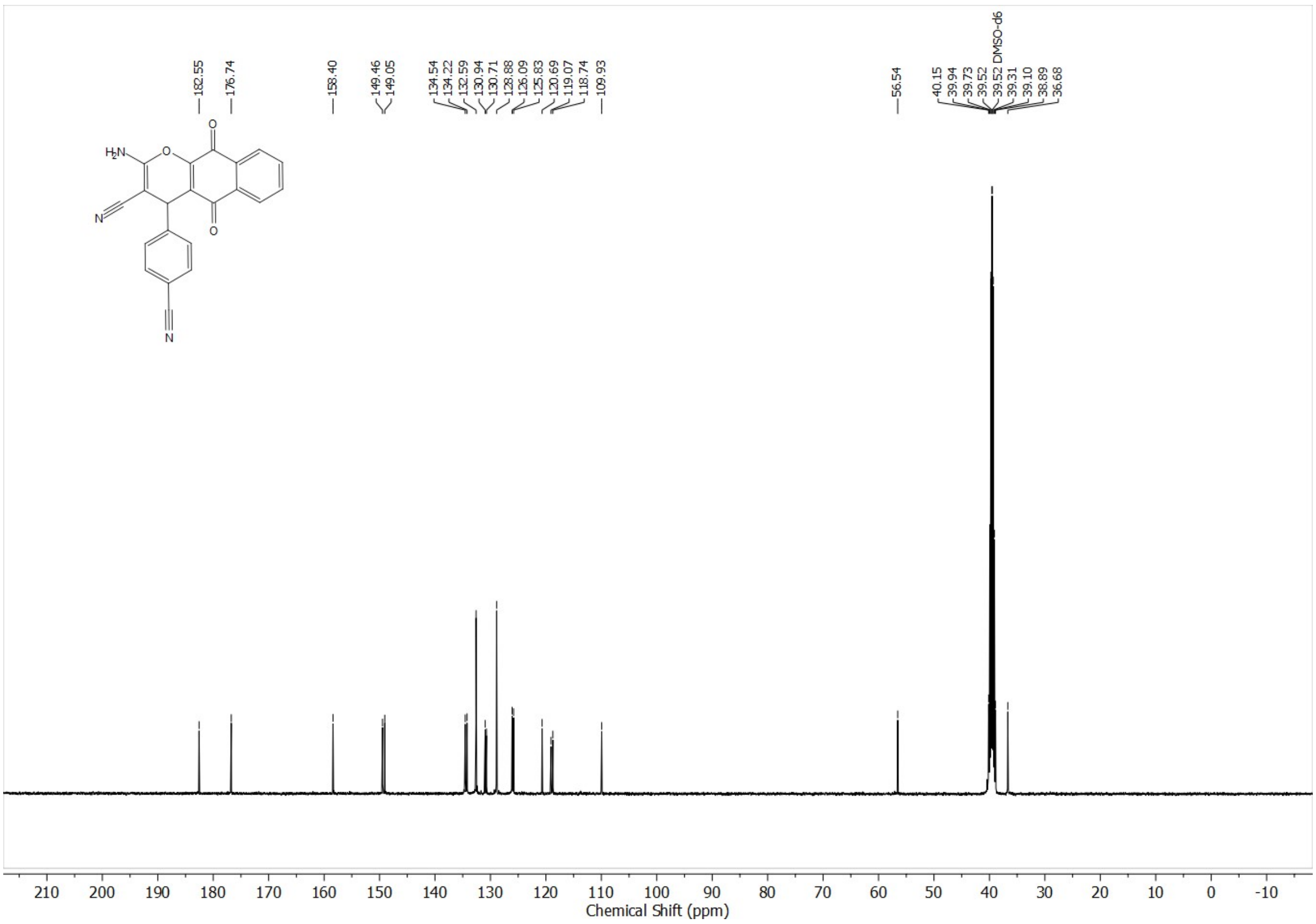

Fig. S5. ^{13}C NMR spectrum of **compound 4b** in DMSO-d_6 (101 MHz).

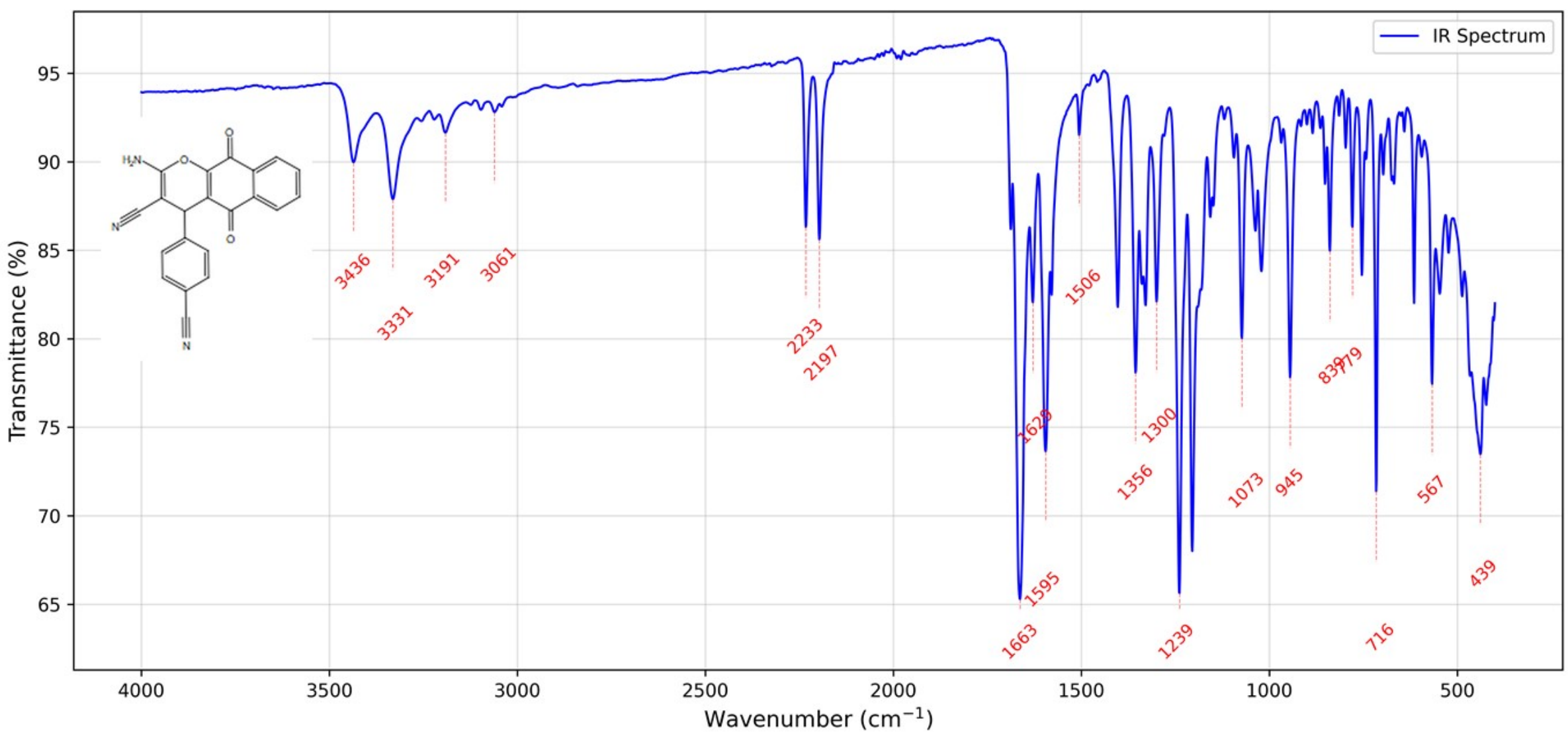

Fig. S6. IR spectrum of **compound 4b** (FT-IR).

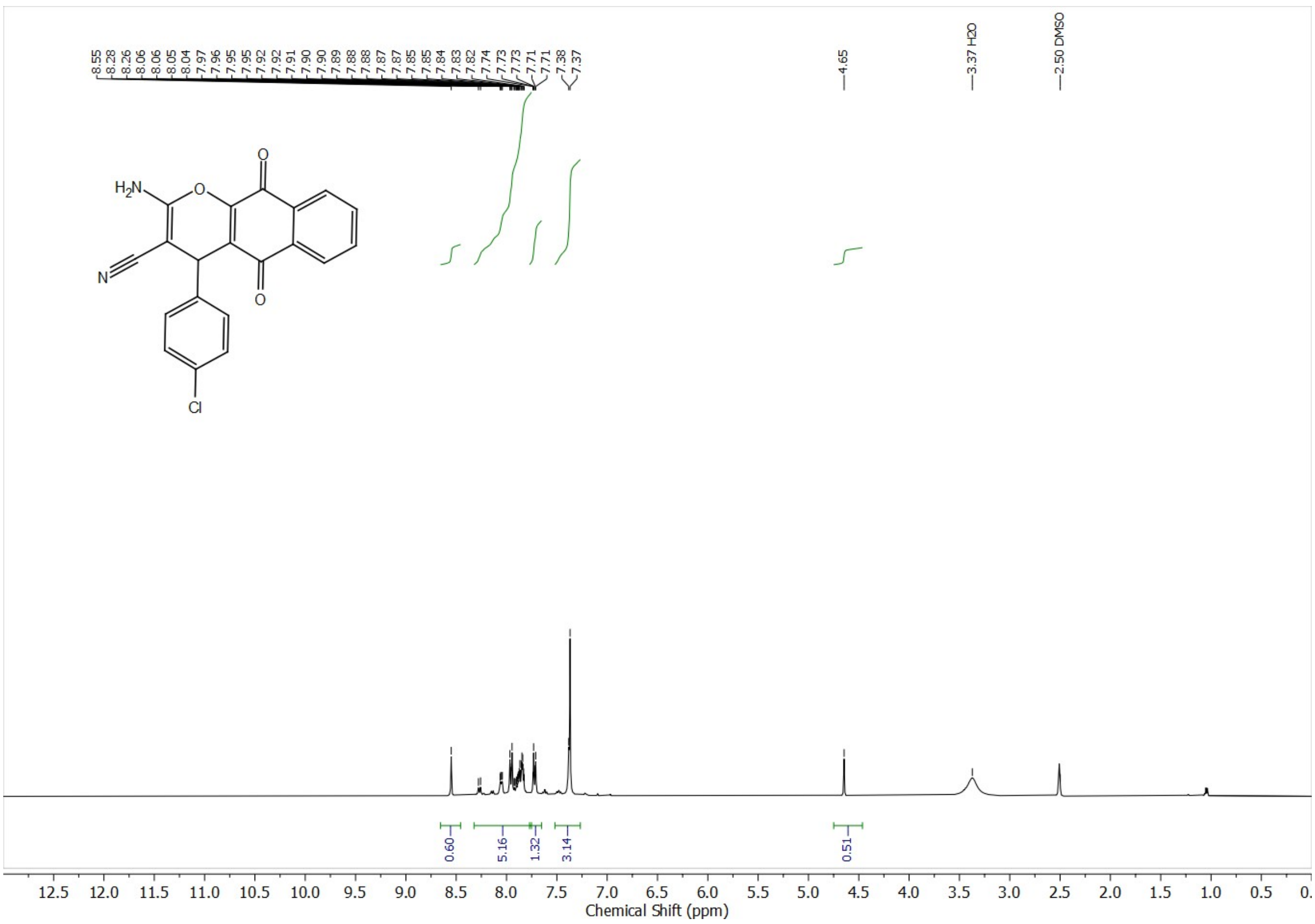

Fig. S7. ^{1}H NMR spectrum of **compound 4c** recorded in DMSO-d_6 (400 MHz).

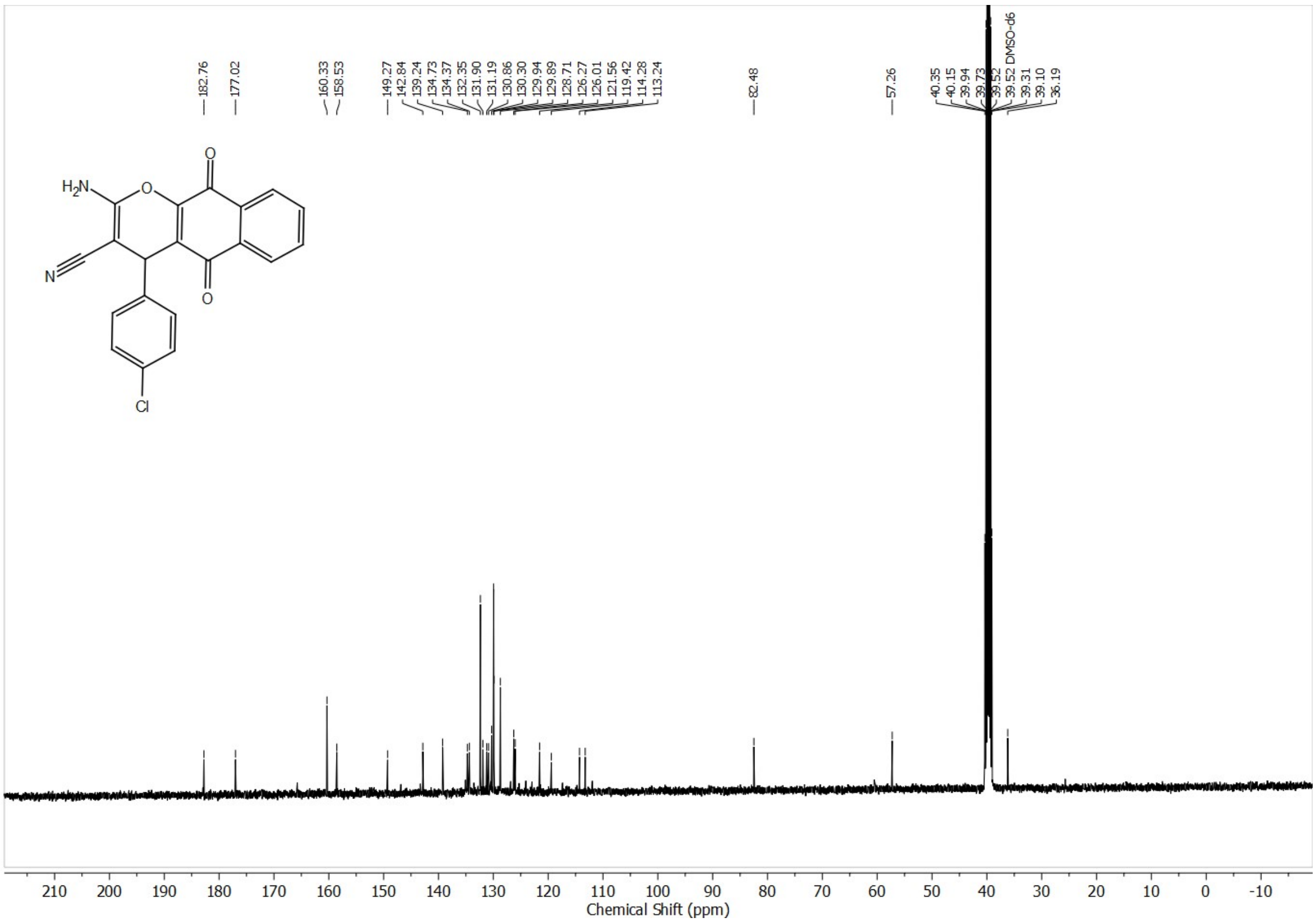

Fig. S8. ^{13}C NMR spectrum of **compound 4c** in DMSO-d_6 (101 MHz).

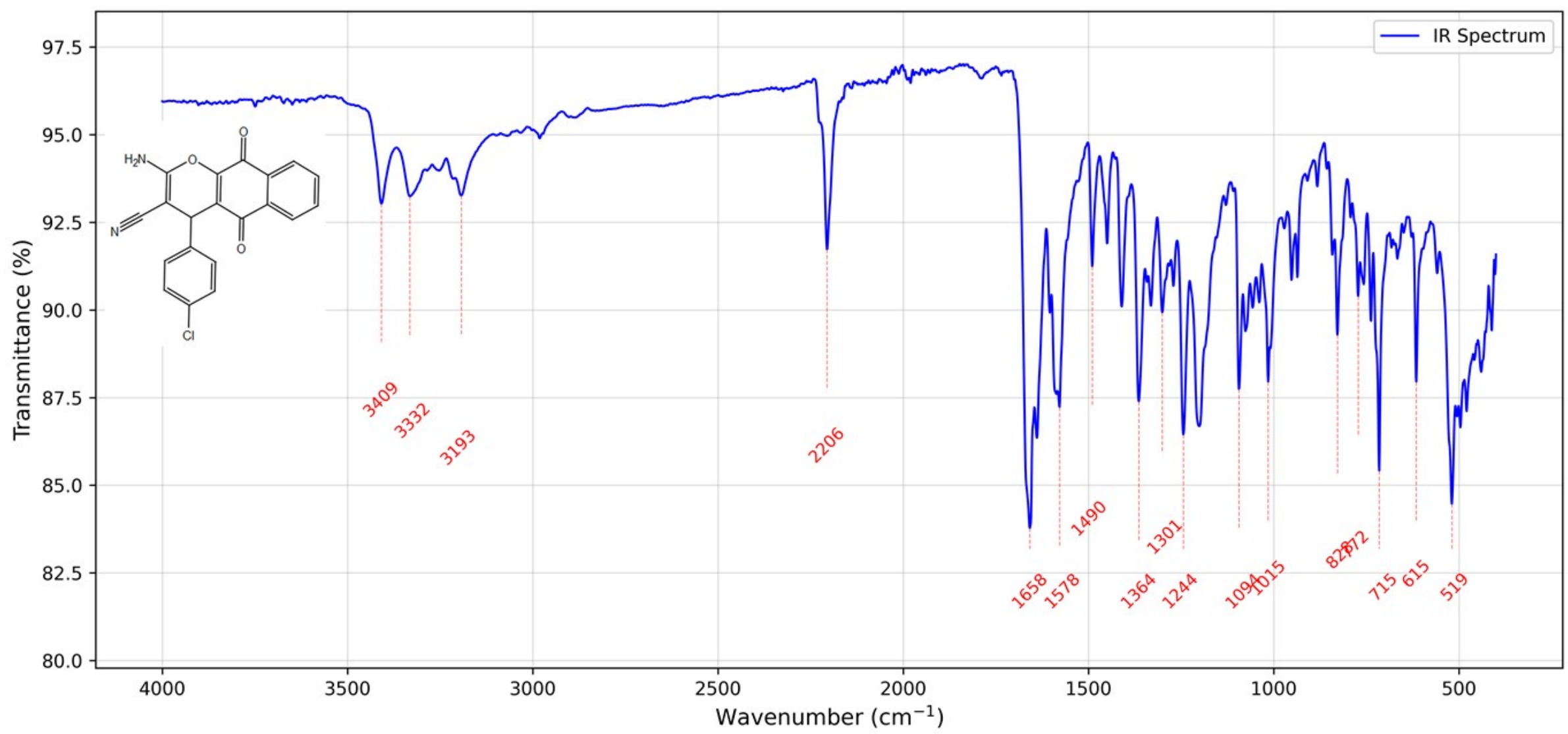

Fig. S9. IR spectrum of **compound 4c** (FT-IR).

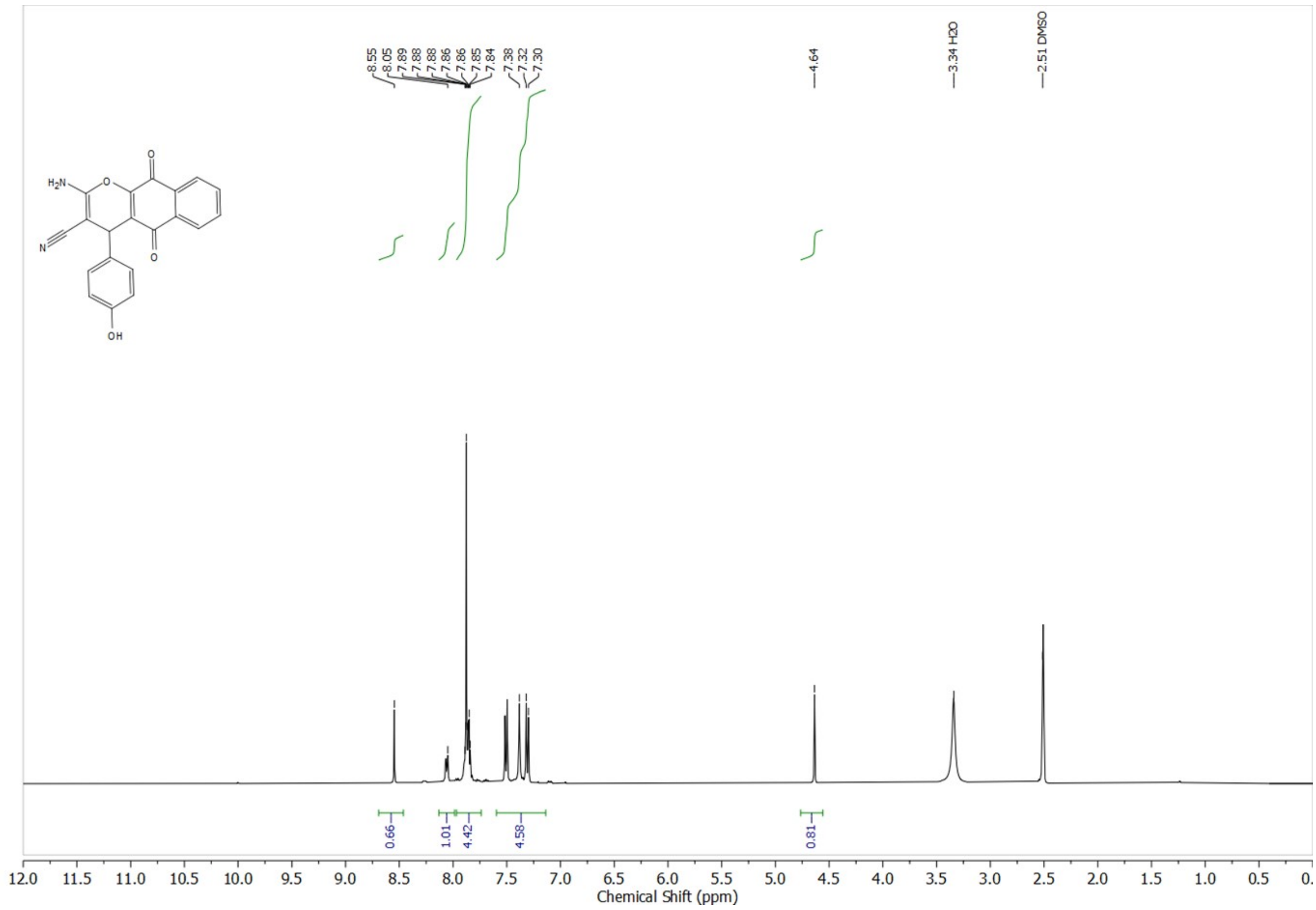

Fig. S10. ^{1}H NMR spectrum of **compound 4d** recorded in DMSO-d$_6$ (400 MHz).

\

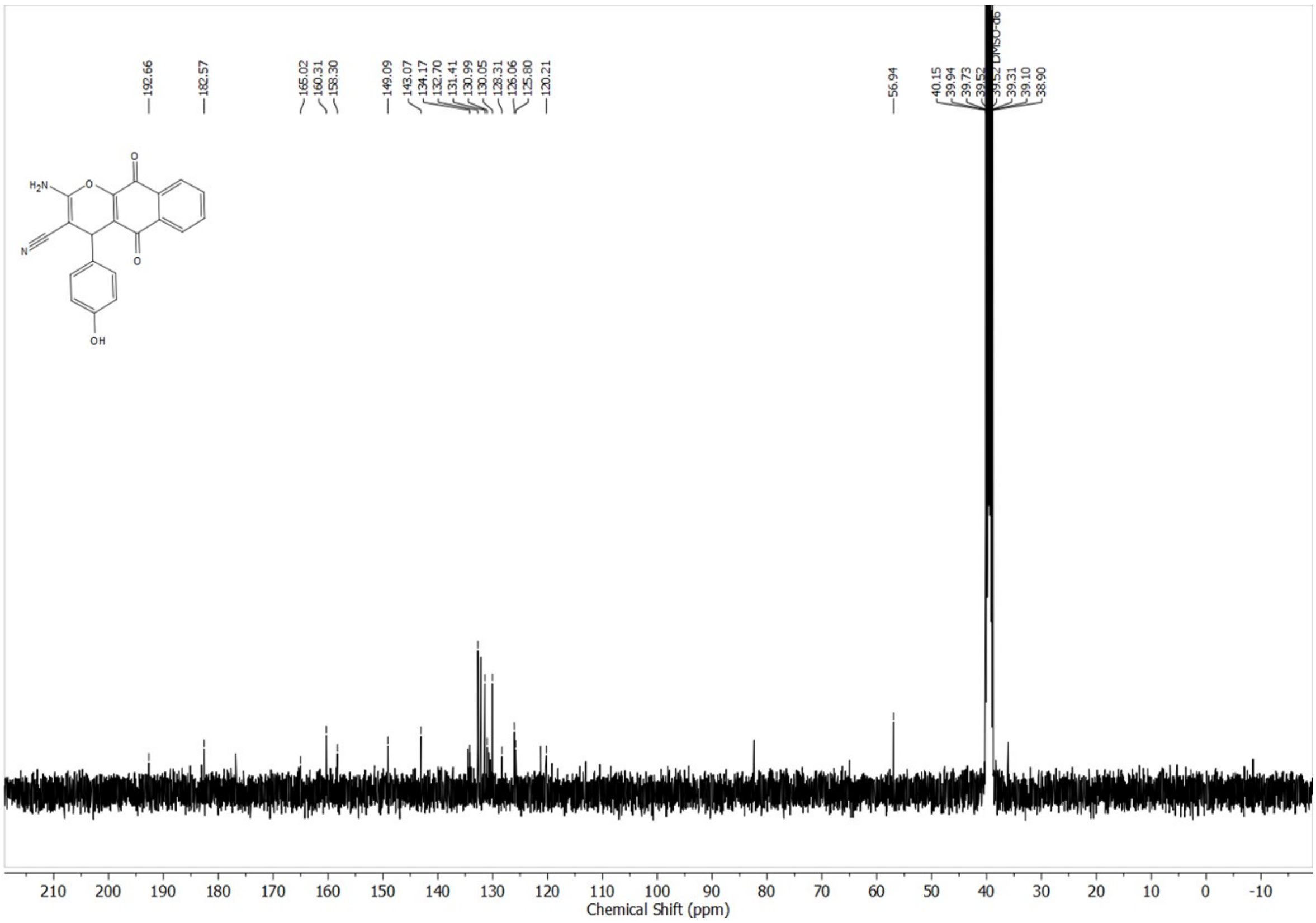

Fig. S11. ^{13}C NMR spectrum of **compound 4d** in DMSO-d$_6$ (101 MHz).

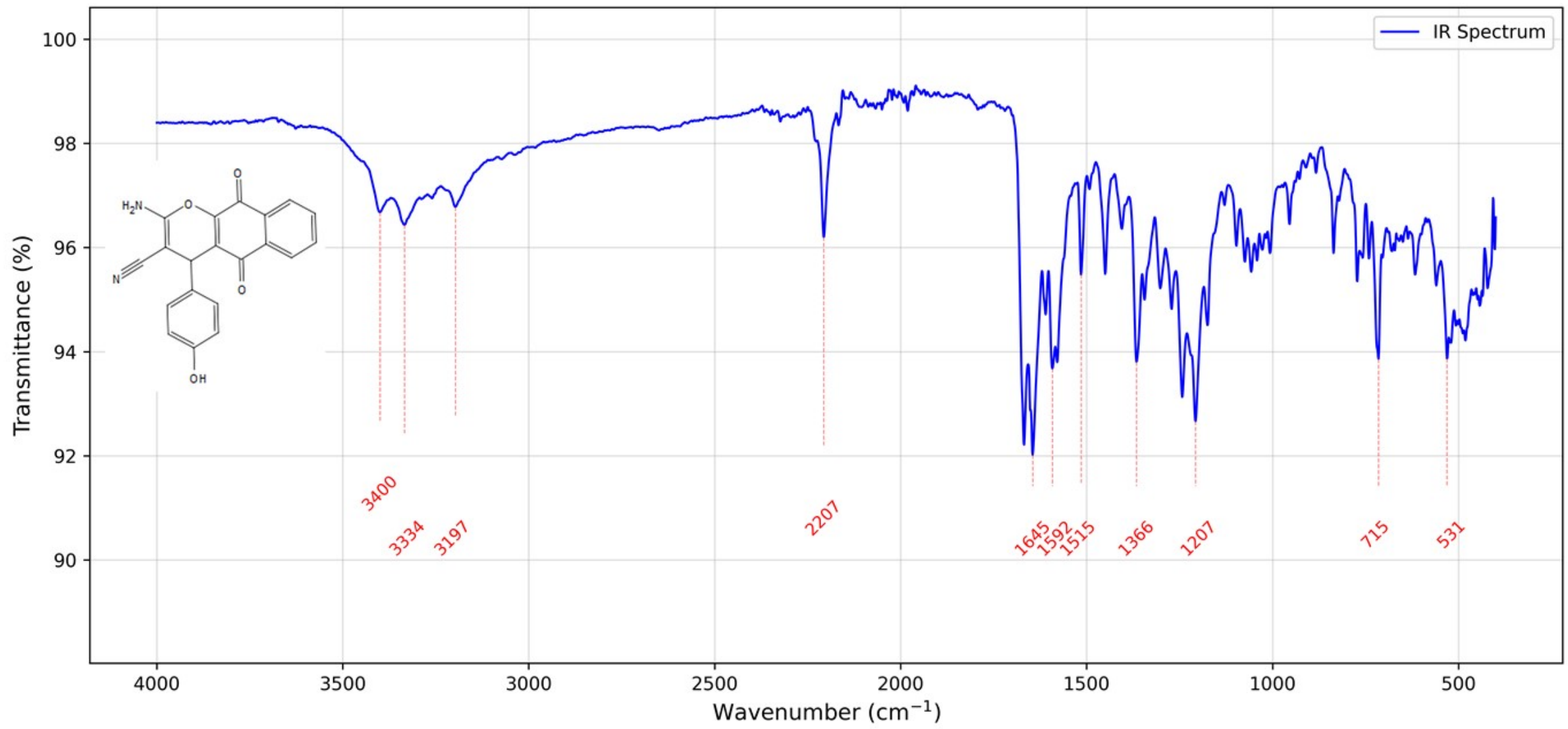

Fig. S12. IR spectrum of **compound 4d** (FT-IR).

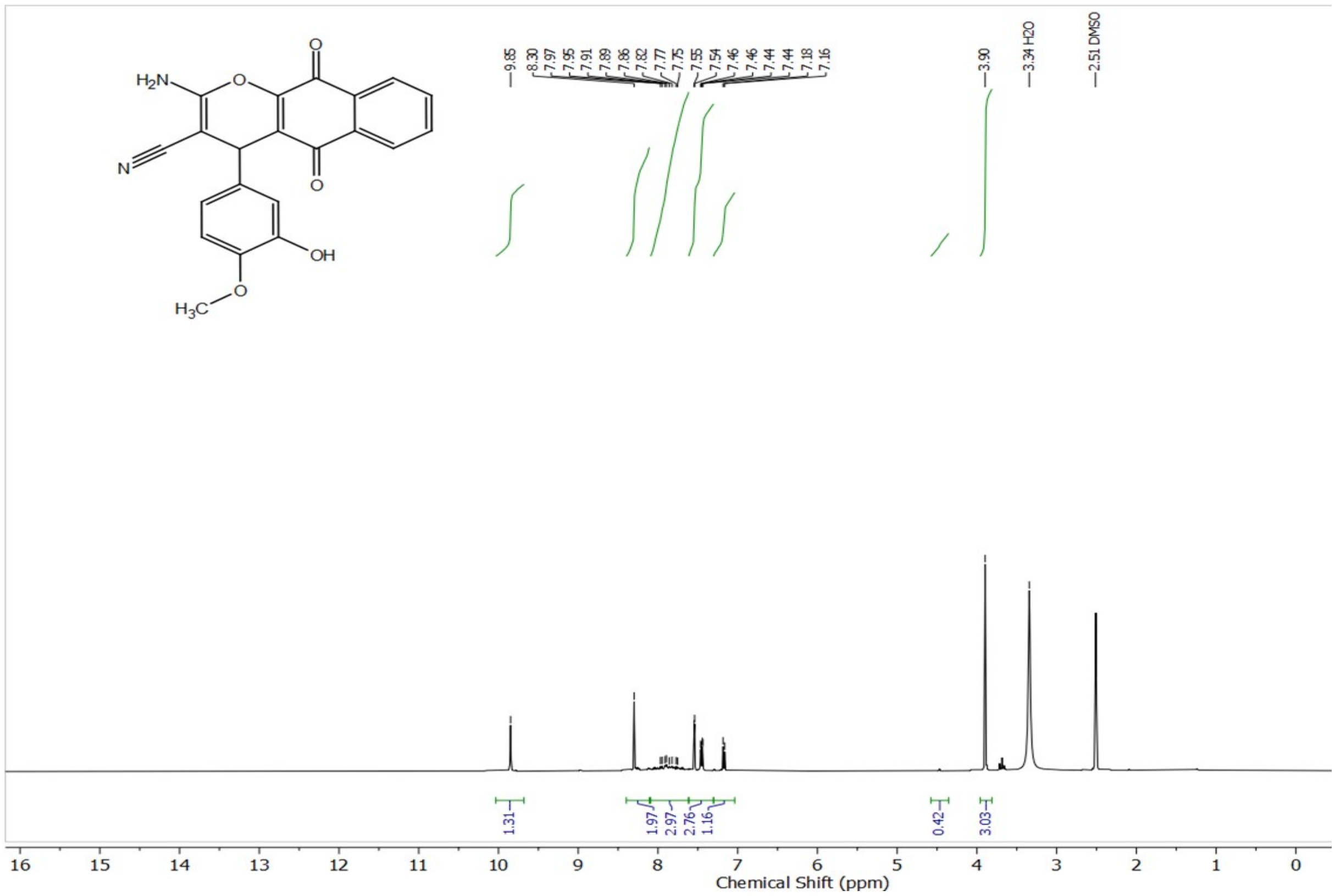

Fig. S13. ¹H NMR spectrum of **compound 4e** recorded in DMSO-d₆ (400 MHz).

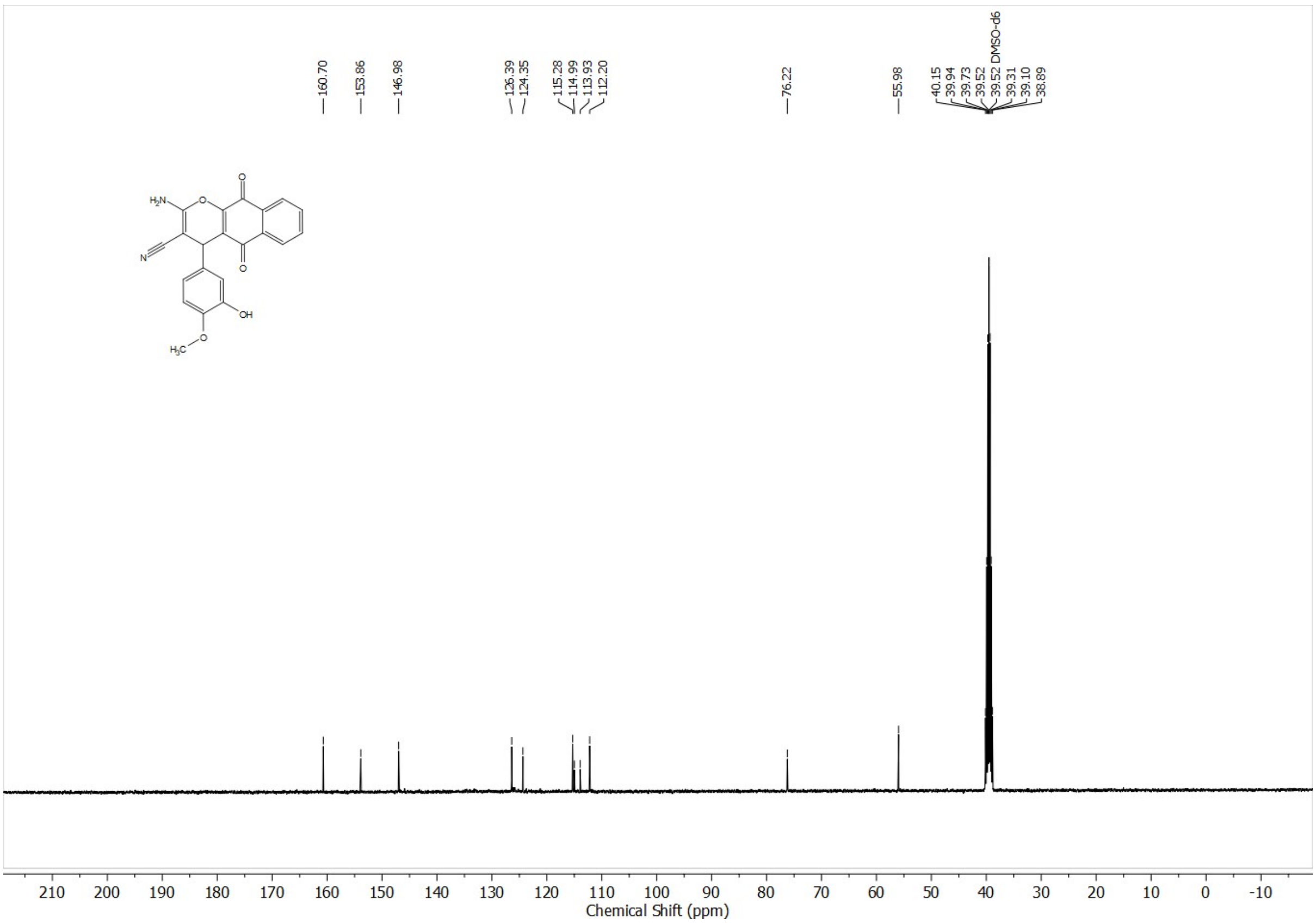

Fig. S14. ^{13}C NMR spectrum of **compound 4e** in DMSO-d$_6$ (101 MHz).

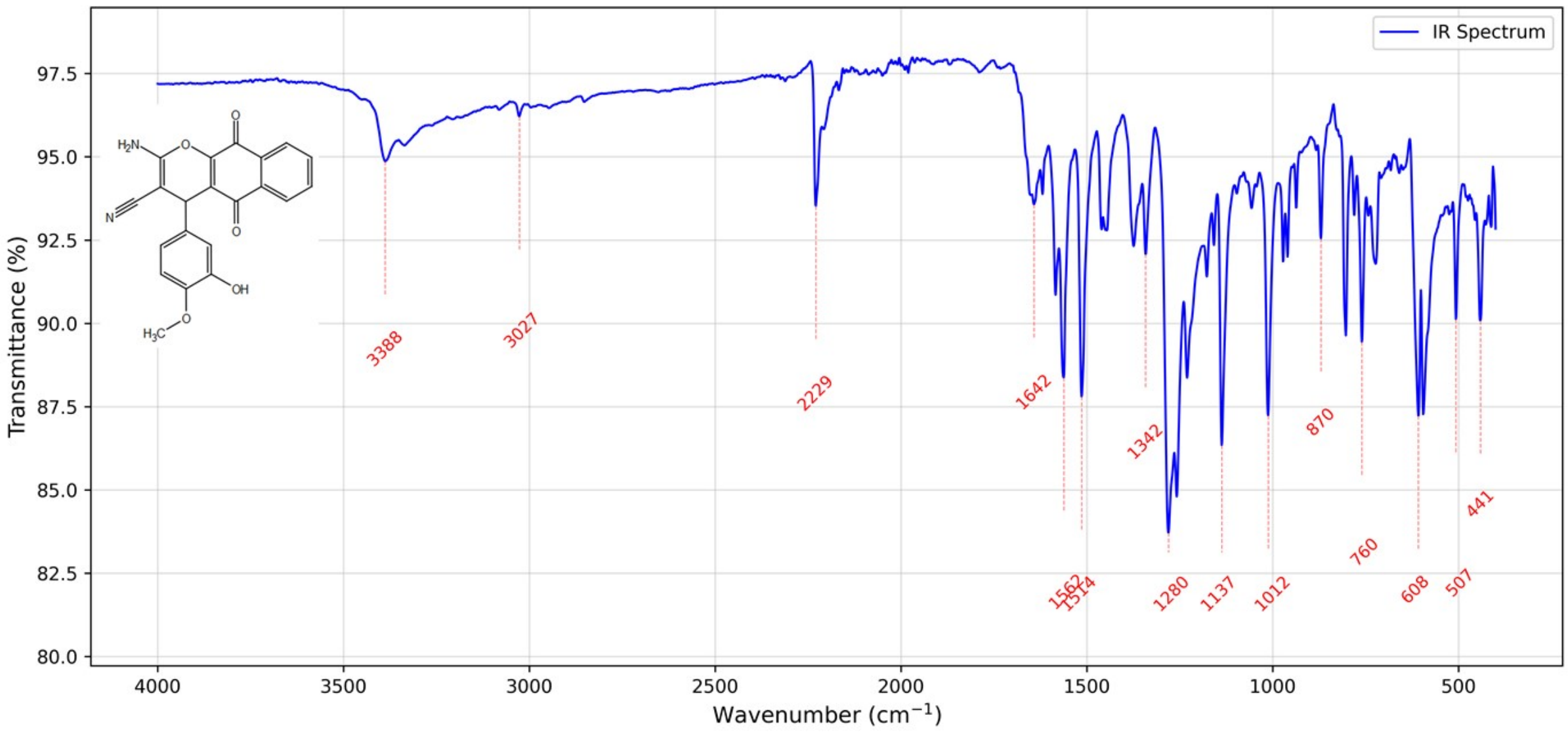

Fig. S15. IR spectrum of **compound 4e** (FT-IR).

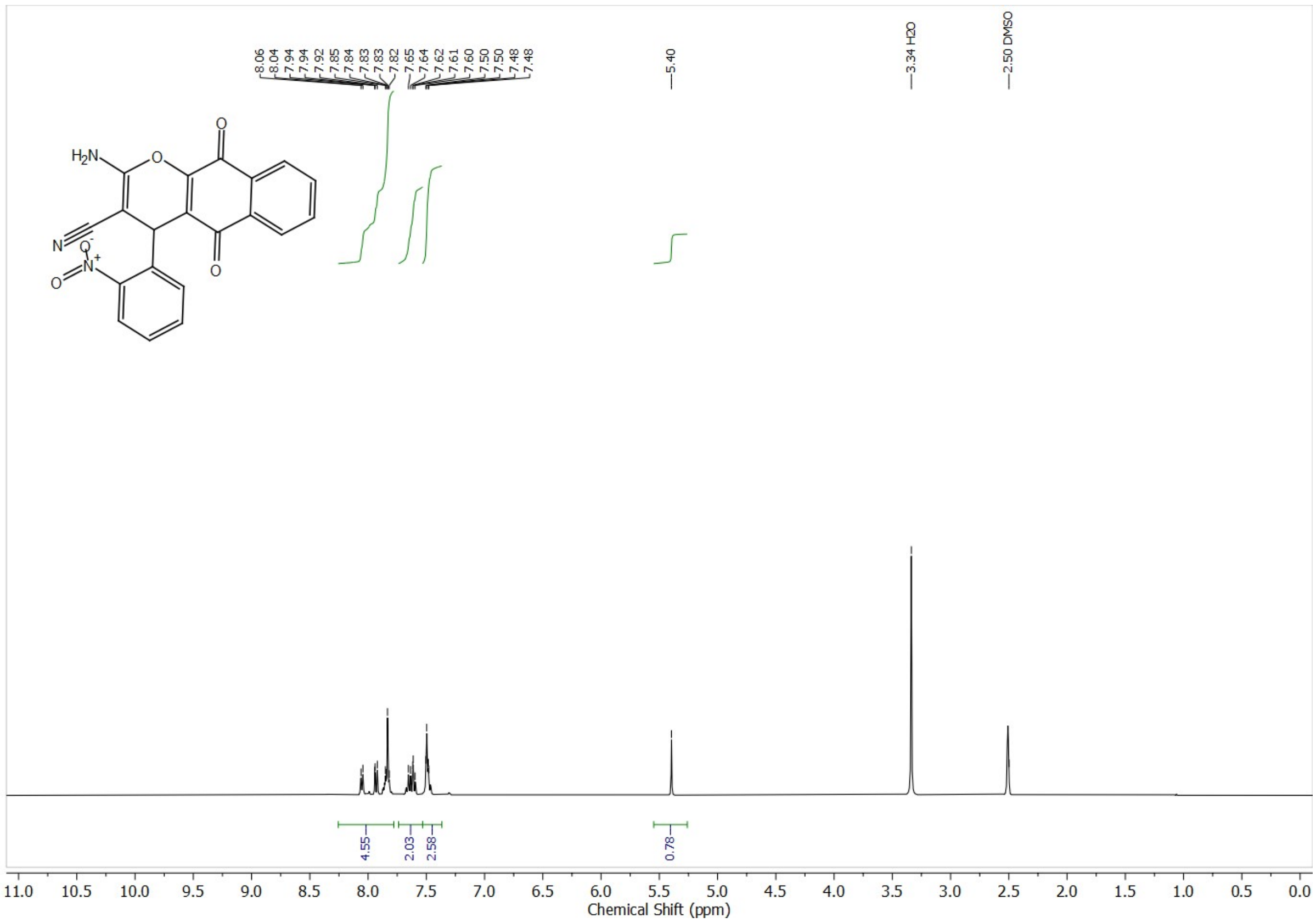

Fig. S16. ^{1}H NMR spectrum of **compound 4f** recorded in DMSO-d_6 (400 MHz).

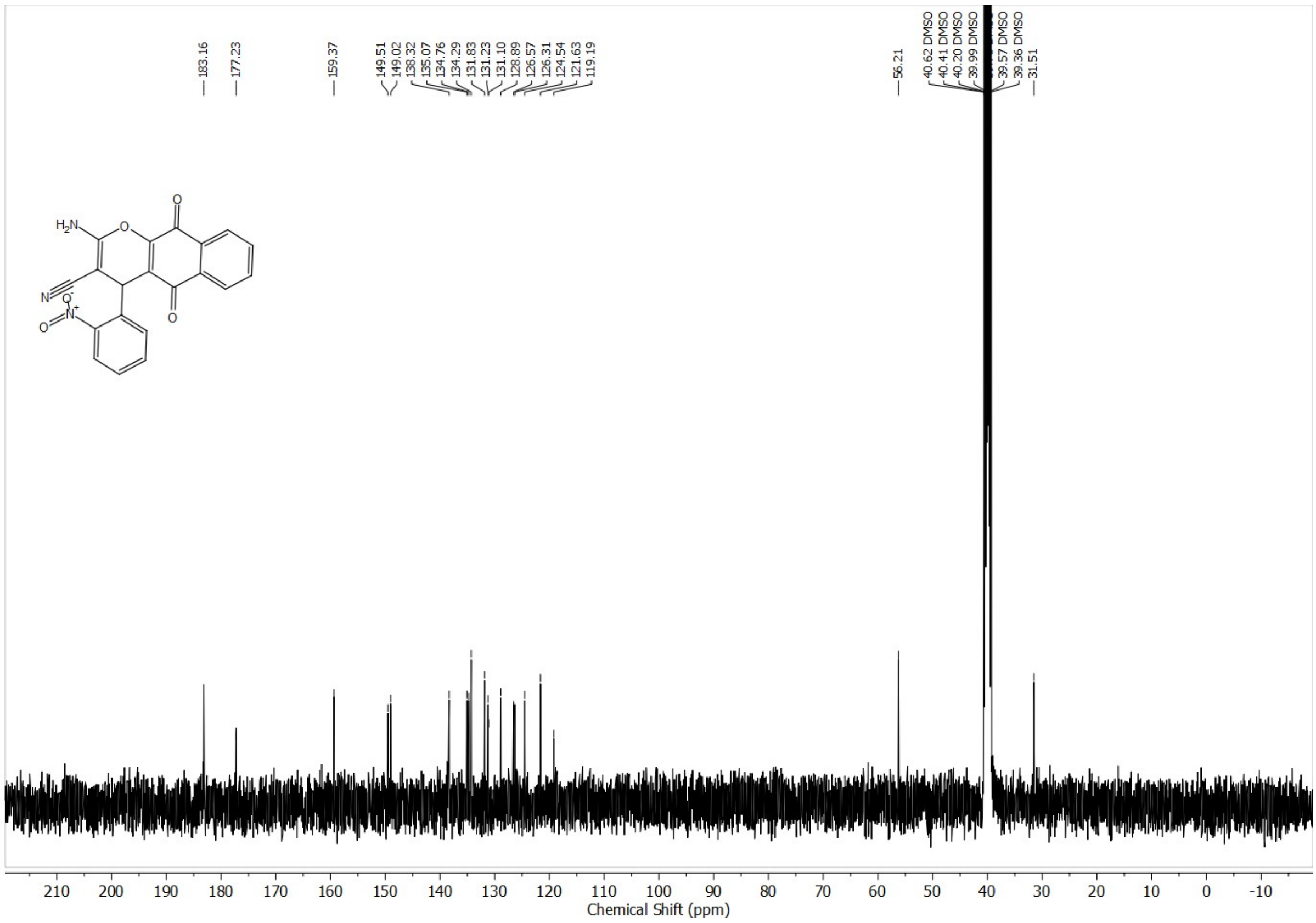

Fig. S17. ^{13}C NMR spectrum of **compound 4f** in DMSO-d_6 (101 MHz).

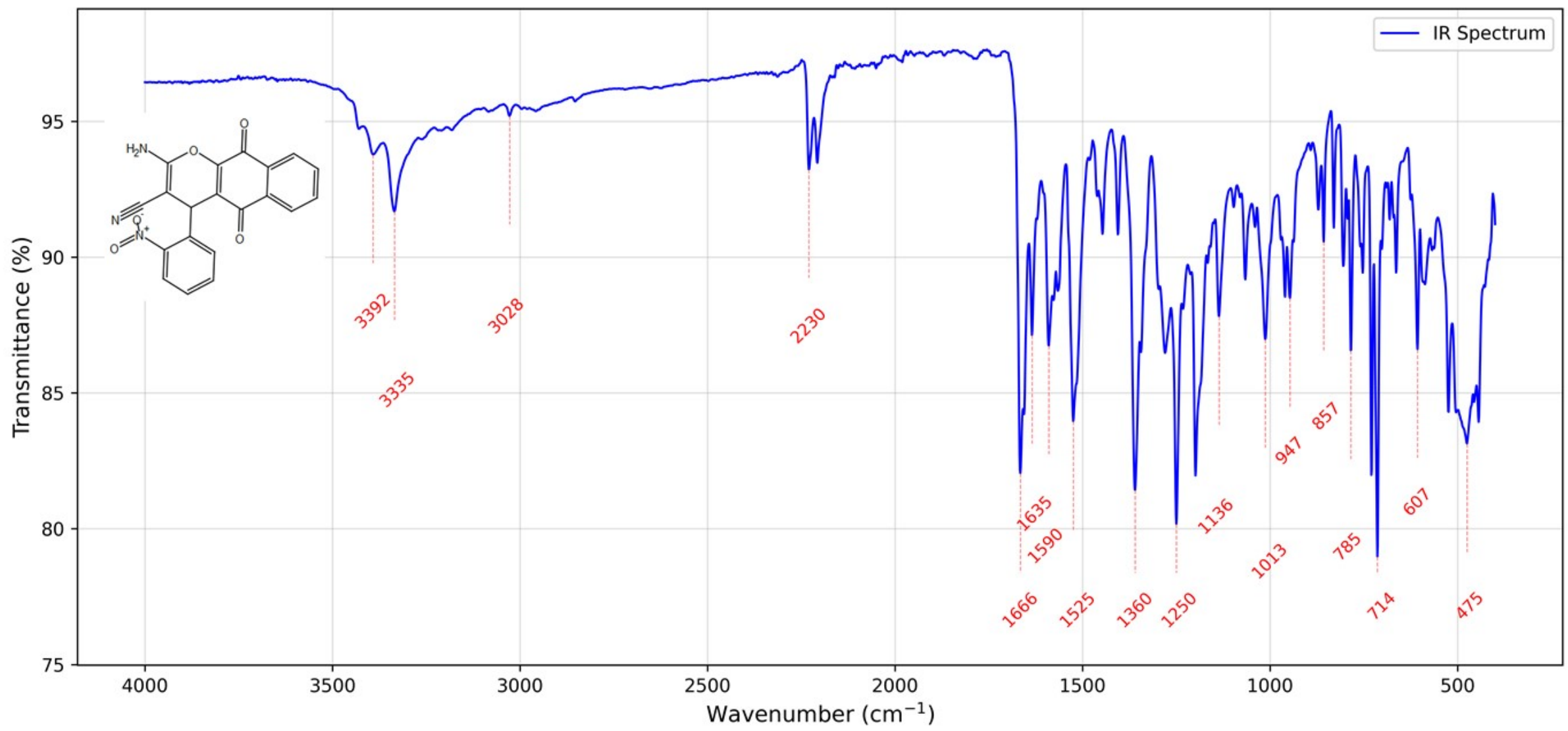

Fig. S18. IR spectrum of **compound 4f** (FT-IR).

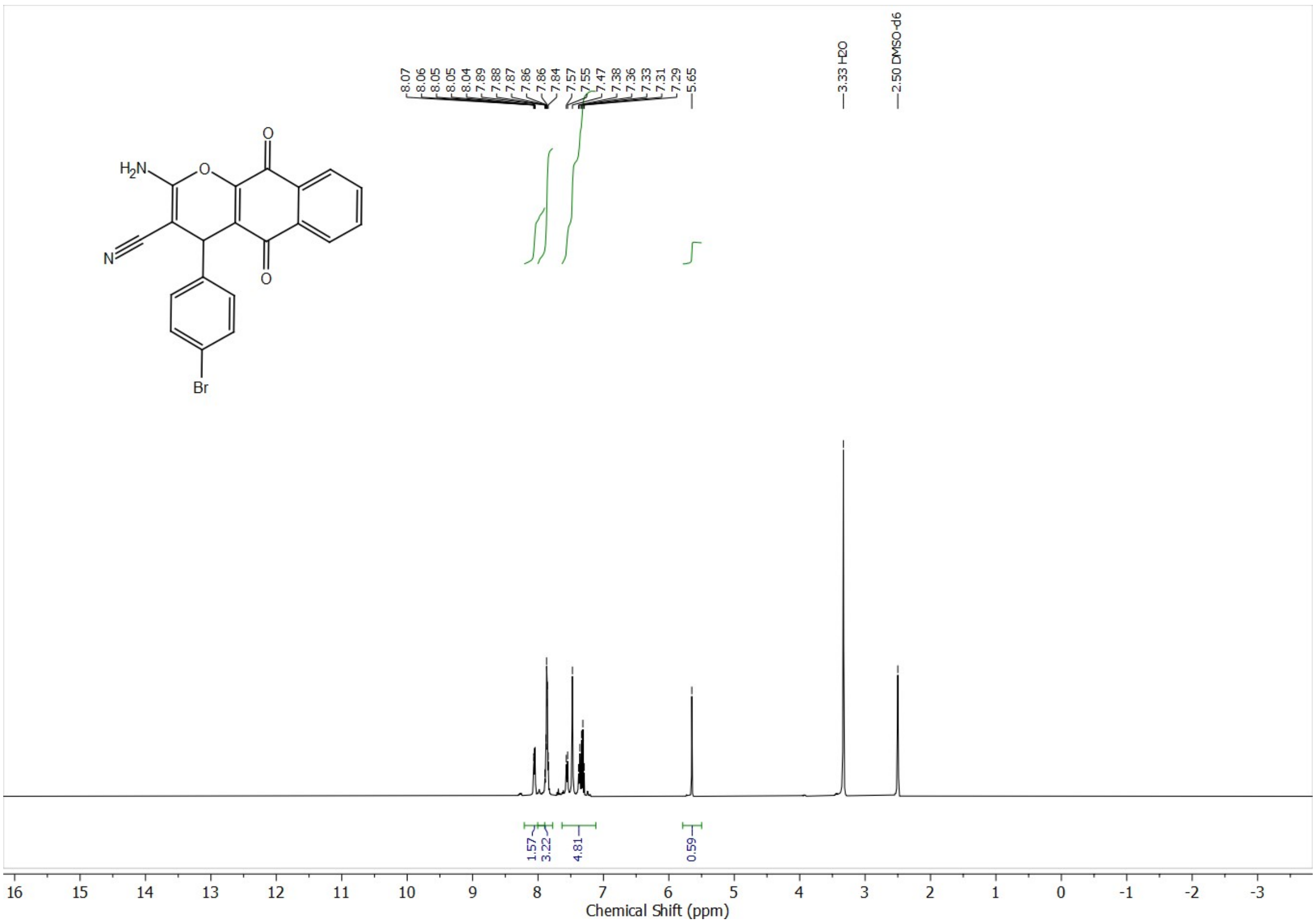

Fig. S19. ^{1}H NMR spectrum of **compound 4g** recorded in DMSO-d_6 (400 MHz).

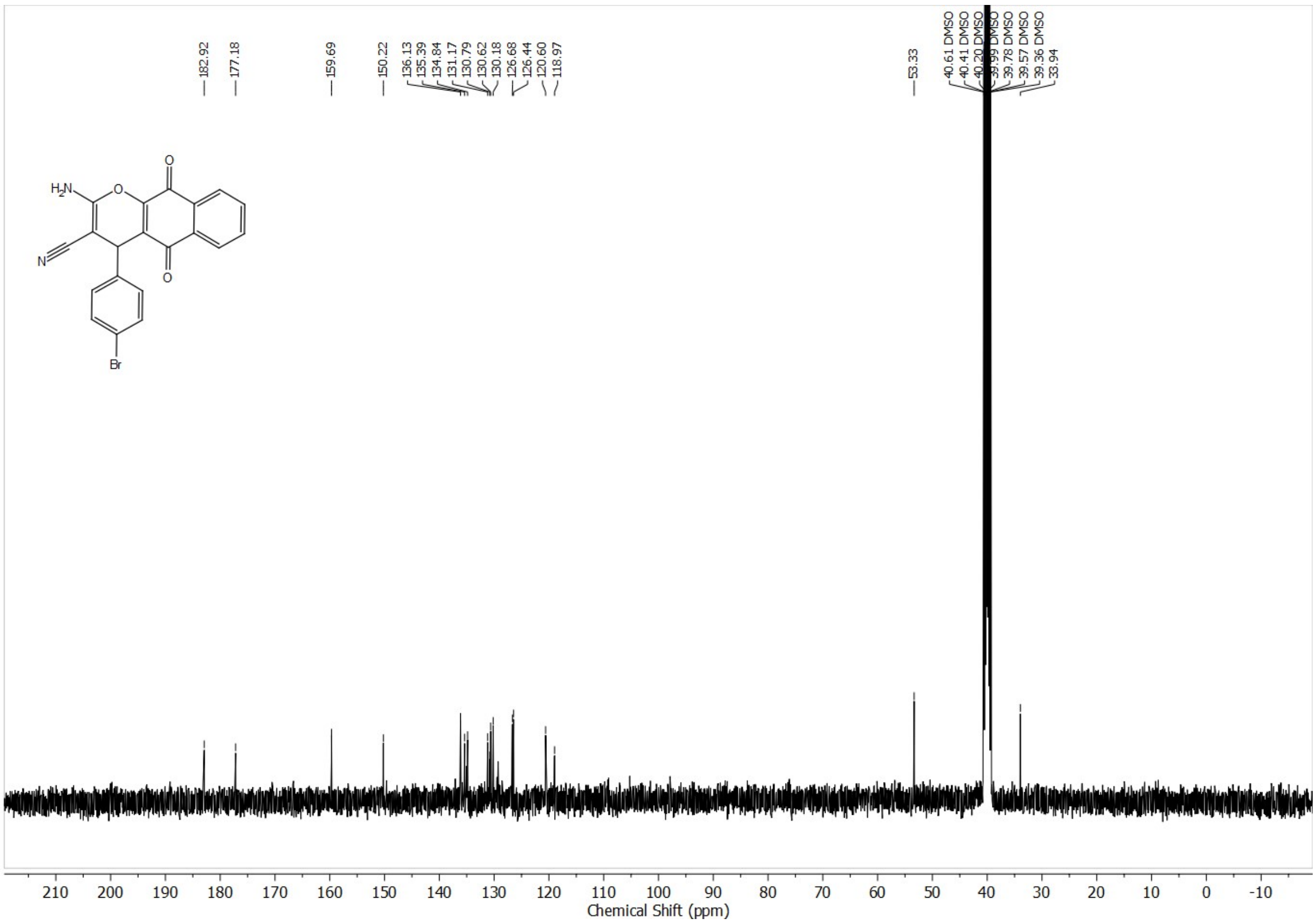

Fig. S20. ^{13}C NMR spectrum of **compound 4g** in DMSO-d_6 (101 MHz).

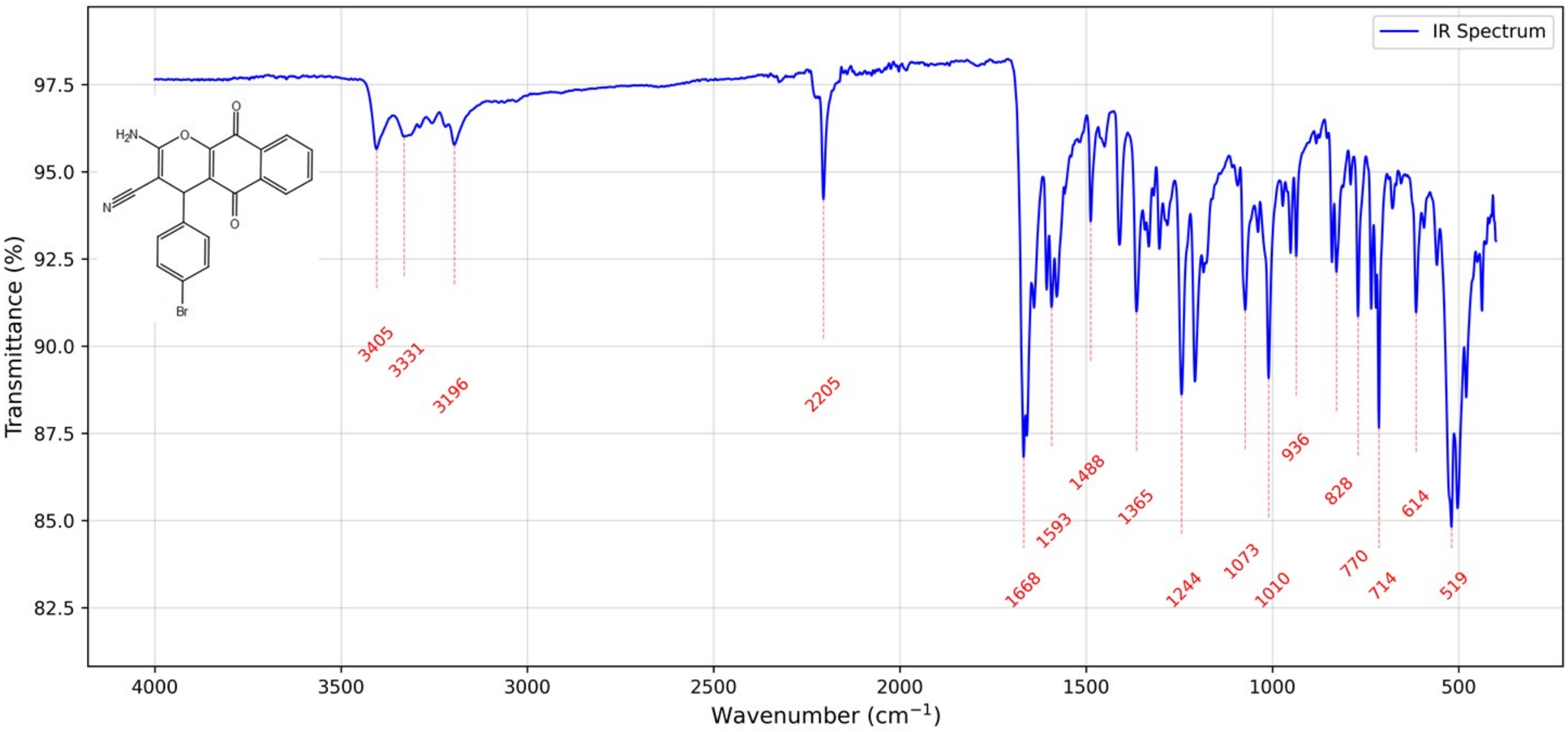

Fig. S21. IR spectrum of **compound 4g** (FT-IR).

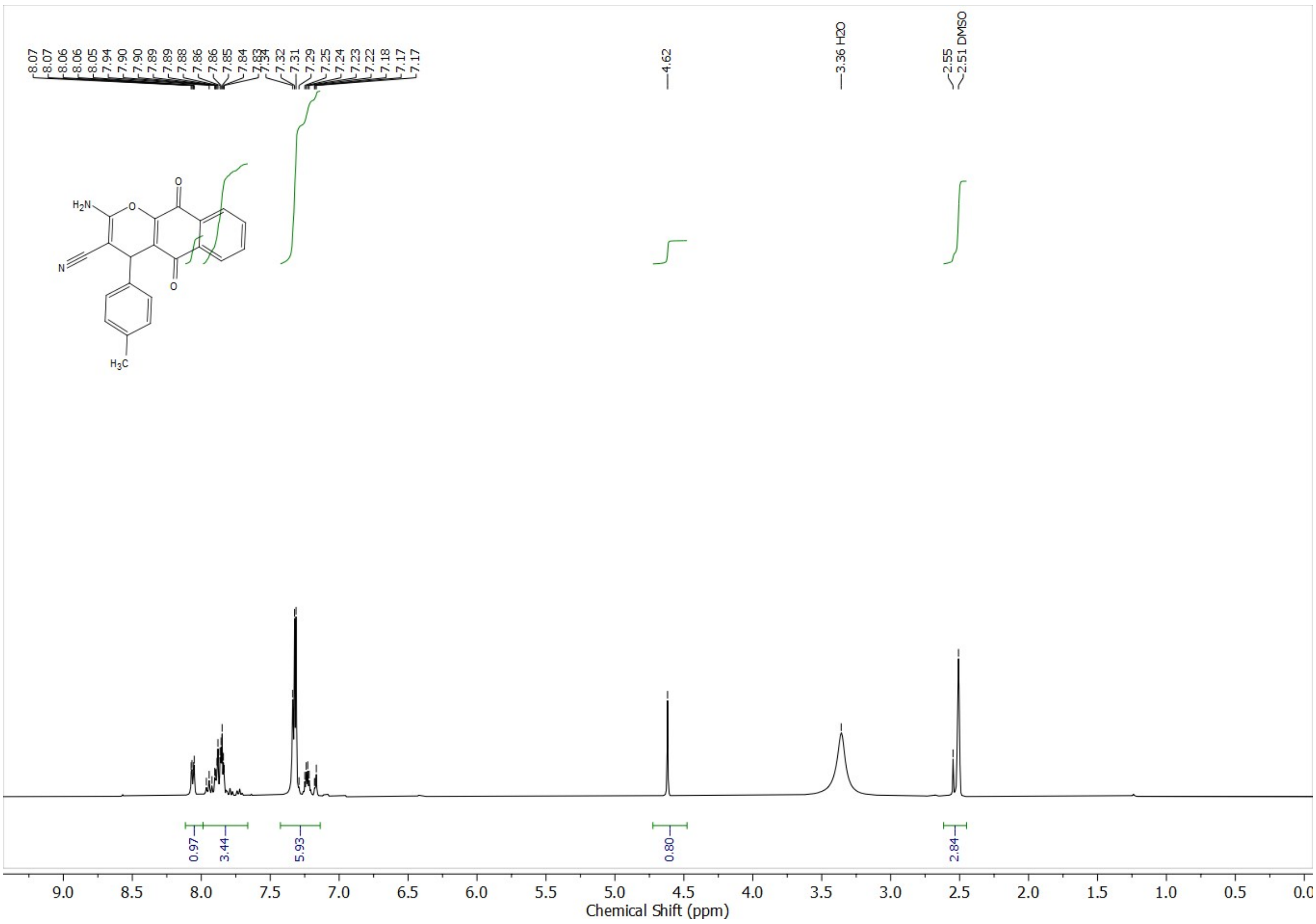

Fig. S22. ^{1}H NMR spectrum of **compound 4h** recorded in DMSO-d_6 (400 MHz).

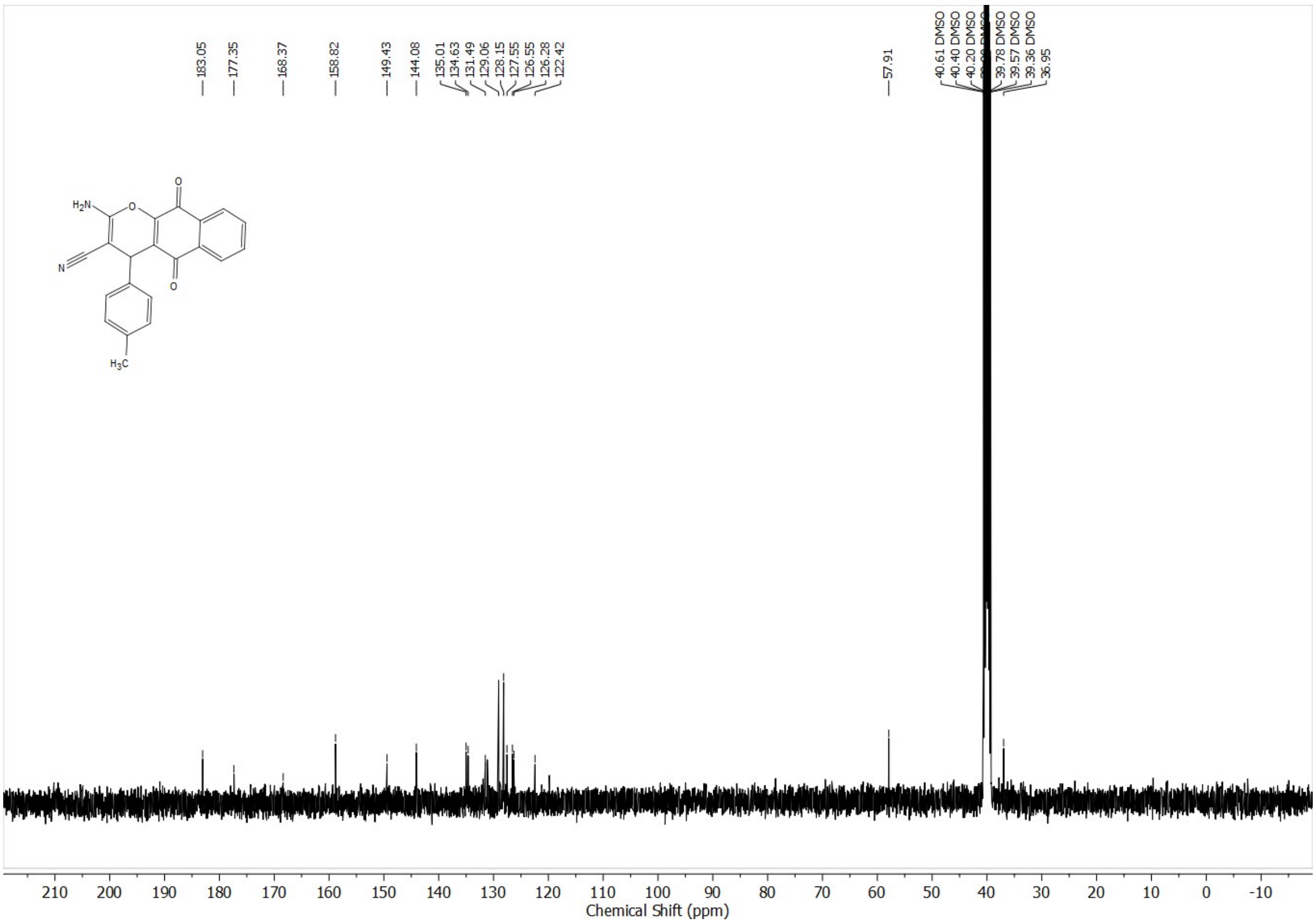

Fig. S23. ^{13}C NMR spectrum of **compound 4h** in DMSO-d$_6$ (101 MHz).

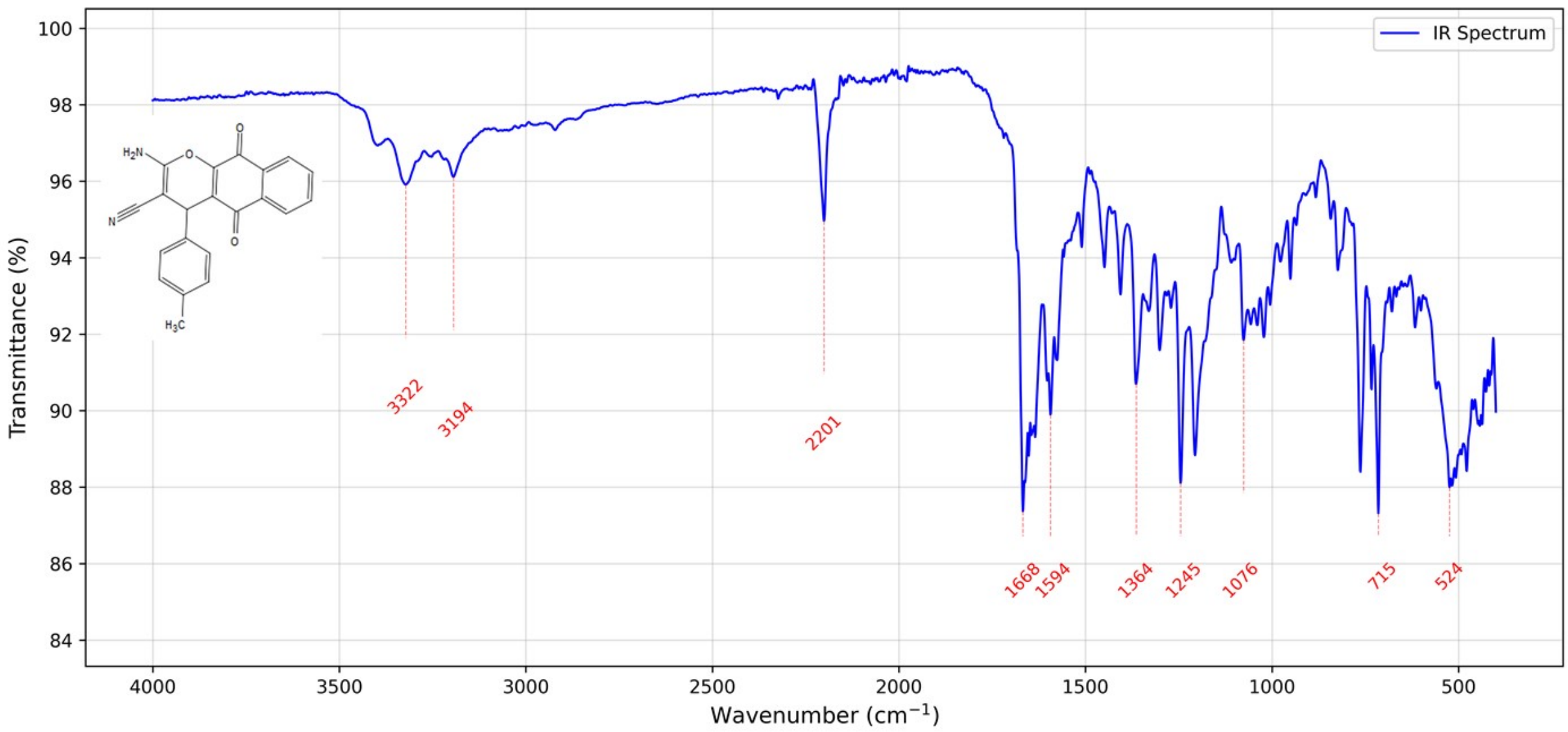

Fig. S24. IR spectrum of **compound 4h** (FT-IR).

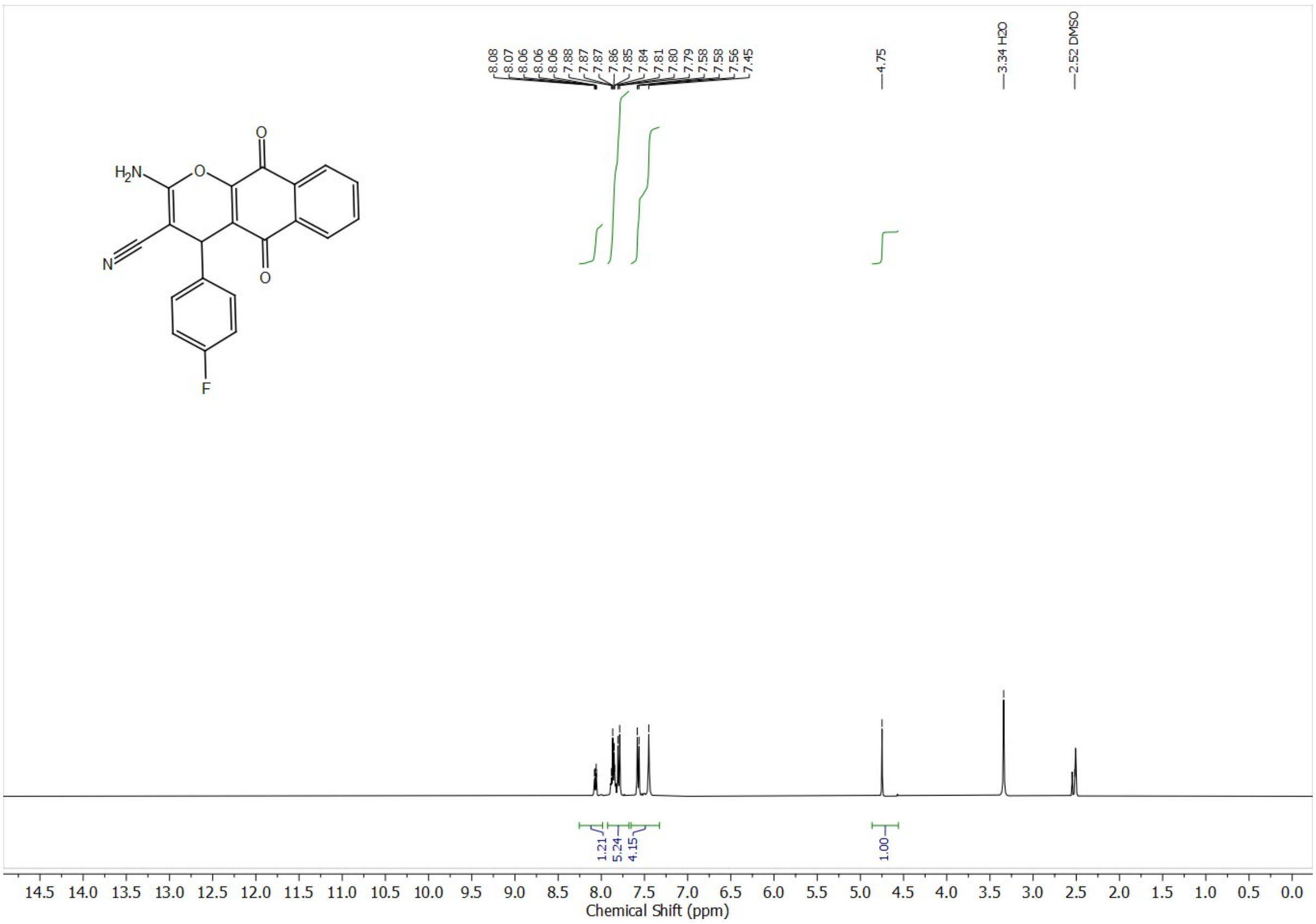

Fig. S25. ^{1}H NMR spectrum of **compound 4i** recorded in DMSO-d$_6$ (400 MHz).

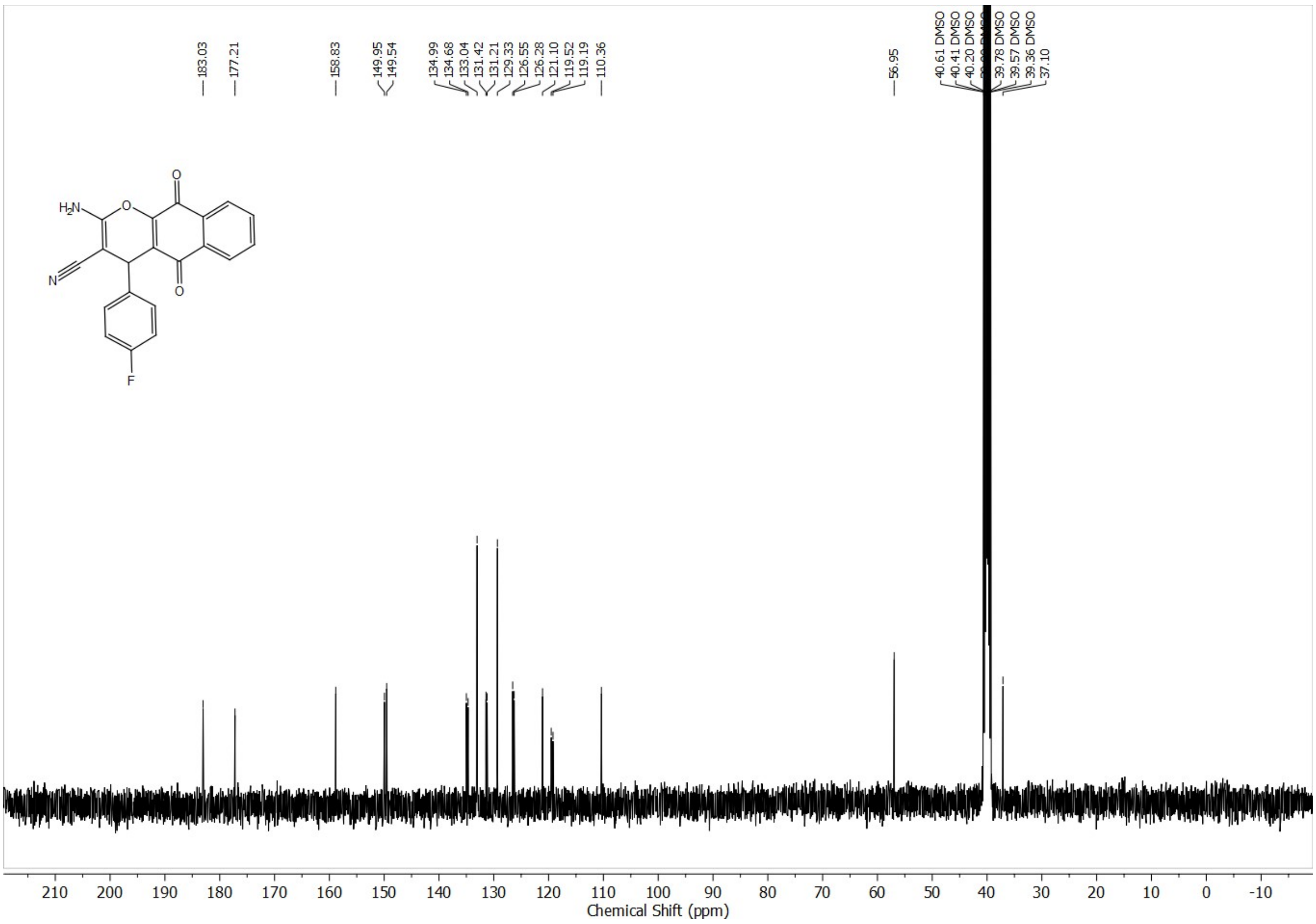

Fig. S26. ^{13}C NMR spectrum of **compound 4i** in DMSO-d_6 (101 MHz).

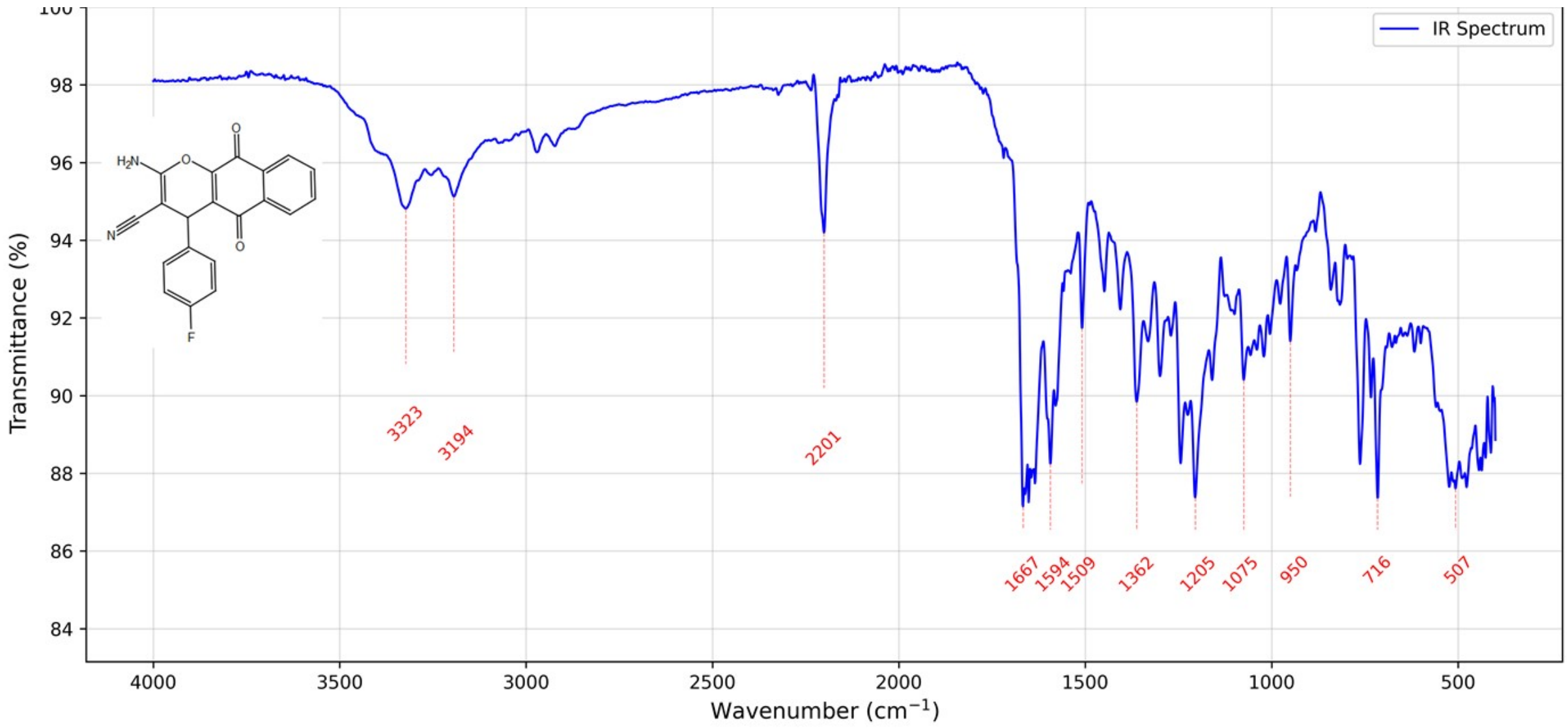

Fig. S27. IR spectrum of **compound 4i** (FT-IR).

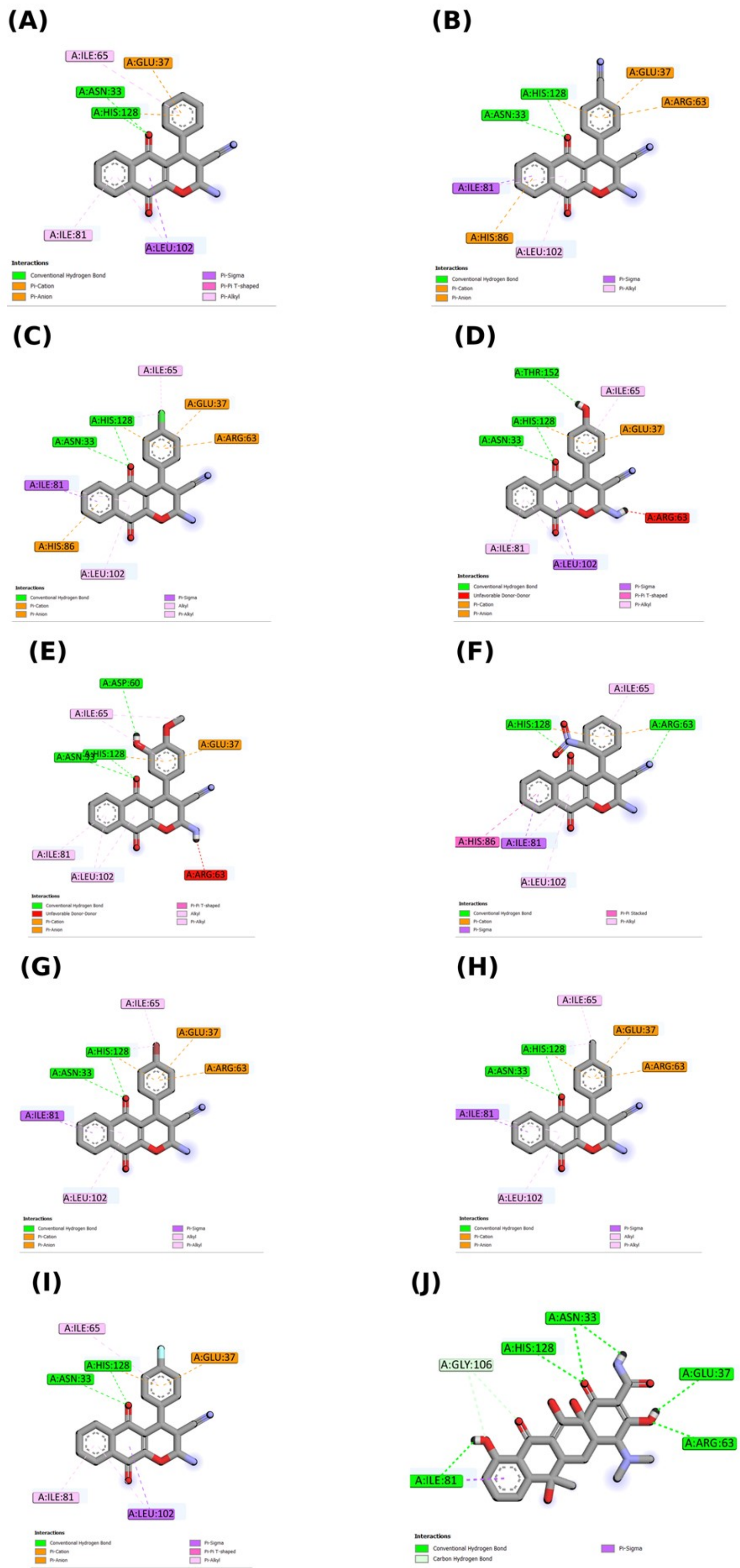

Fig. S28 Two-dimensional protein–ligand interaction diagrams of benzo[g]chromene derivatives (A–I) within the active site of the *Acinetobacter baumannii* target (PDB: 7PQM). Key interactions include hydrogen bonding, π–π stacking, and hydrophobic contacts with residues such as Asn33, His128, Glu37, Arg63, and Ile81, highlighting the binding mode of the synthesized compounds. (J) represents the interaction profile of the reference drug tetracycline within the same binding pocket for comparison.

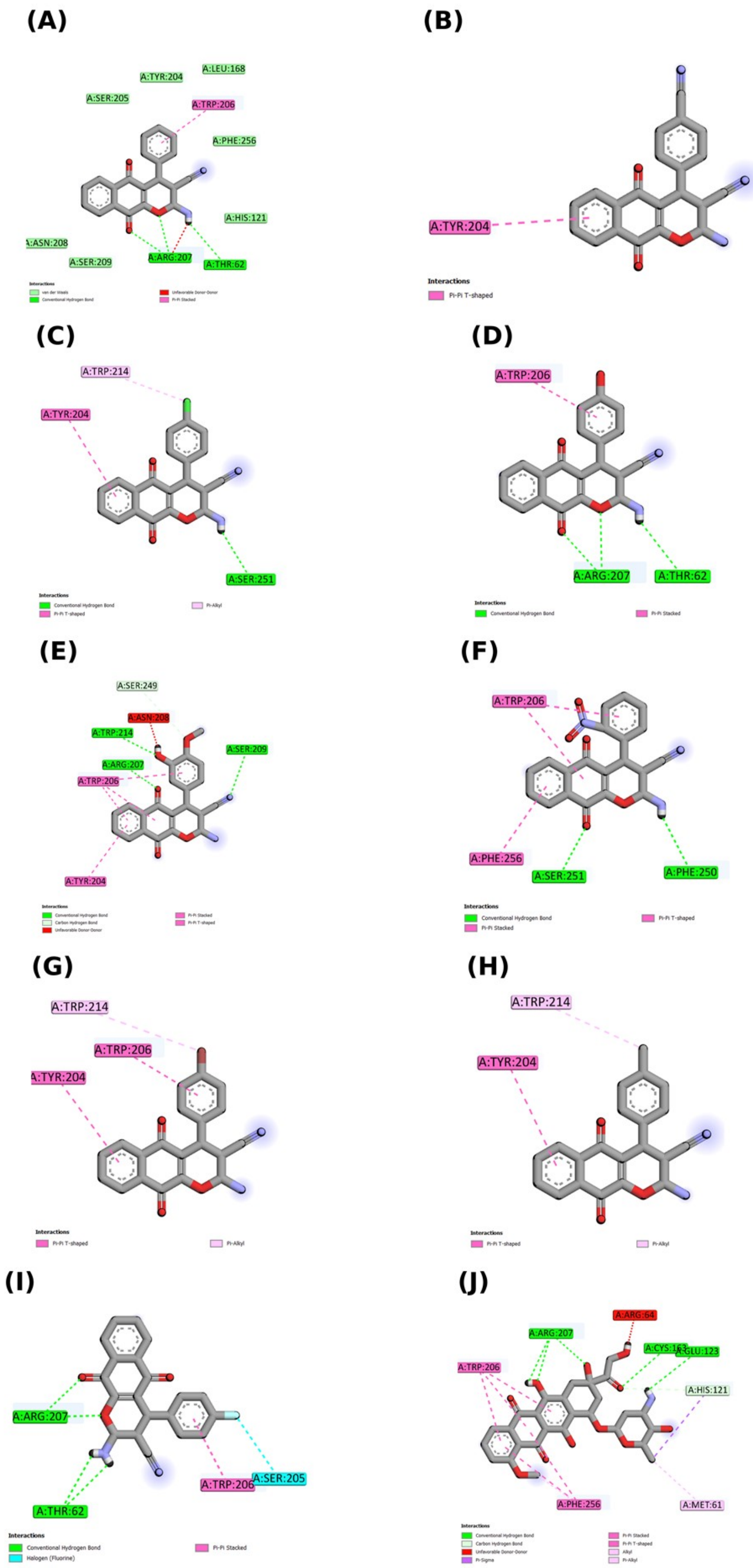

Fig. S29 Two-dimensional protein–ligand interaction diagrams of benzo[g]chromene derivatives (A–I) within the active site of the HeLa-associated protein (PDB: 5I9B). The compounds exhibit key interactions such as conventional hydrogen bonding, π–π stacking, and hydrophobic contacts with residues including Tyr204, Trp206, Arg207, Ser205, Thr62, and Phe256, indicating their binding orientation and stability within the pocket. (J) represents the interaction profile of the reference drug doxorubicin within the same active site for comparison.

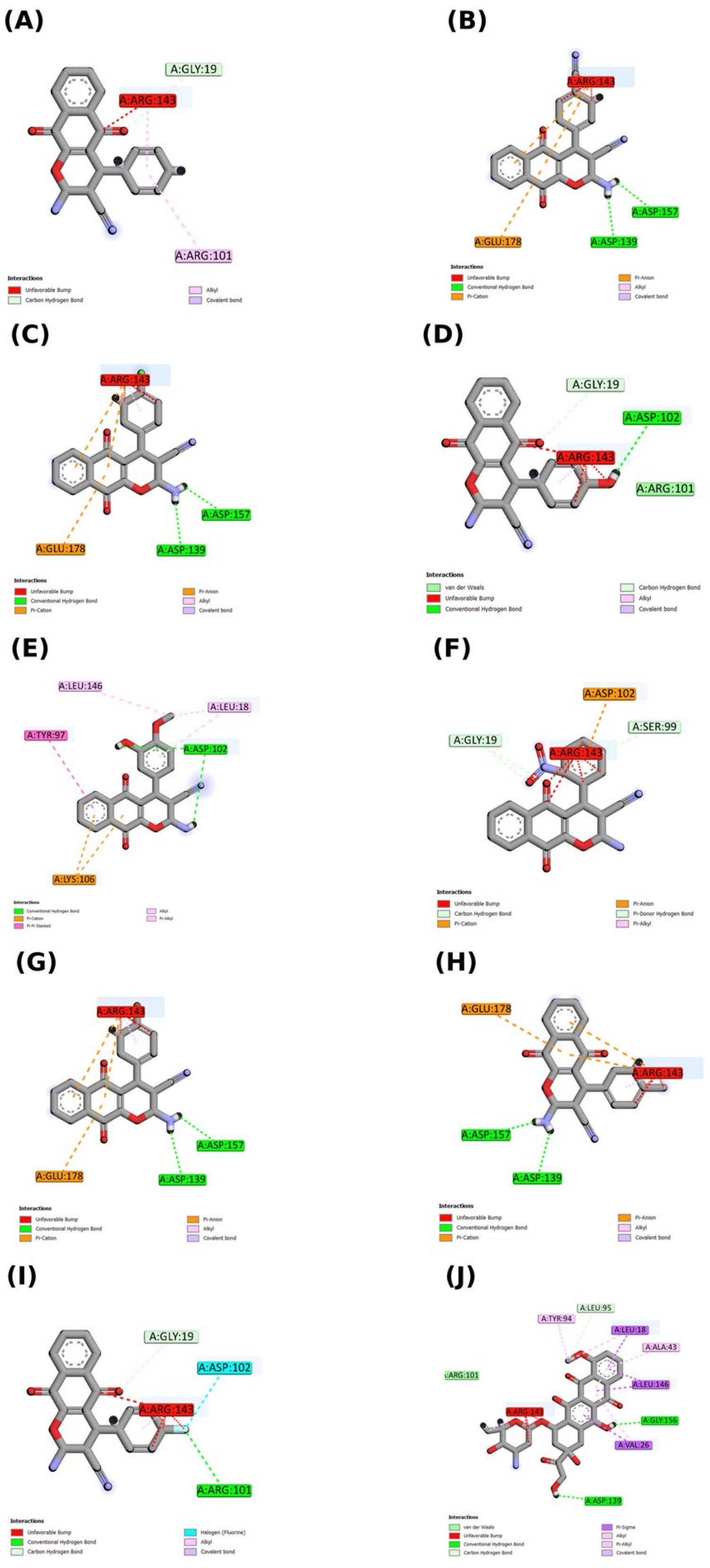

Fig. S30 Two-dimensional protein–ligand interaction diagrams of benzo[g]chromene derivatives (A–I) within the active site of the MDA-MB231-associated target protein (PDB: 3KRR). The ligands exhibit key interactions including conventional hydrogen bonding, π–π stacking, and hydrophobic contacts with residues such as Arg143, Asp102, Asp139, Asp157, Glu178, and Gly19, indicating their binding orientation and stabilisation within the active pocket. (J) represents the interaction profile of the reference drug doxorubicin, highlighting its binding pattern for comparison with the synthesized derivatives.

Section S3. MTT Assay

3.1 Maintenance and sub-culturing of cell lines

The above-mentioned all cell lines were purchased from National Center for Cell Science complex (NCCS), Pune India, and these cell lines were grown at 37 ^{0}C in a humidified CO_2 incubator with 95% air and 5% CO_2 supply in a DMEM (Dulbecco's modified Eagle's) medium supplemented with 10% FBS (fetal Bovine Serum, and 200 IU/ml penicillin, 200g/ml streptomycin antibiotics, cells were maintained aseptically by changing the medium on every other day, and once 80% confluence reached, cells were sub-cultured by following trypsinization method.

3.2 Cell count:

Cells once reached to 80% confluence, they were subjected to subculture by trypsinization method- briefly, the media was aspirated off aseptically, given 1X-PBS wash to remove dead cells, and aspirated PBS, added 1 ml Trypsin EDTA to the cell culture dish and incubated @ 37^{0}C for three minutes, after incubation, neutralized Trypsin-EDTA action by adding 1:2 ratio of DMEM medium to the dish, collected the solution into 15ml eppendorf and centrifuged briefly for 5 minutes at 1700 RPM and discarded supernatant, re-suspended pellet in 1ml fresh media, counted the cells by using Neubauer chamber by using Trypan blue dye exclusion method, and seeded on 96 well plates at a density of 5 x 10^3 cells and incubated overnight at 37^{0}C in a humidified incubator with 5% CO_2 supply.

3.3 Drug treatments and MTT assay for compounds:

The MTT assay was performed for checking the cytotoxicity levels of the compounds **4a-4i** were done as per described protocol previously [1,2]. Compounds were prepared as 10mM main stocks and from that various working stocks were prepared i.e., 0.1, 1, 10, 20, 40, 60, 80 and 100 µM were prepared by diluting in serum free media. After overnight incubation, the media was aspirated off, different concentrations of drug (control (without drug), 0.2% DMSO, 0.1, 1, 10, 20, 40, 60, 80 and 100 µM and Doxorubicin were administrated to the cells as treatment for 24 & 48 hours in a volume of 100µl per well in triplicates, and cells without drug used as a negative control and 02% DMSO as positive control. At the end of the treatment for 24 and 48 hours, the media was aspirated carefully through the corners of the plate without disturbing the cells, then added 20µl of MTT (5mg/ml in 1x-PBS solution) and 80µl of fresh serum free media, and cells were further incubated for 3 to 4 hours as per standard methods during the incubation the crystals were formed with dead cells. After incubation aspirated media, and to dissolve the crystals, 100µl of DMSO was added to each well to dissolve the reduced MTT

crystals. Absorbance was measured at 540nm on a microplate reader (Tecon), two independent experiments were performed in triplicates. The cell responses were calculated using control as the 100 % reference. The mean ± SE was calculated and reported cell response (%) versus drug concentration(µM).

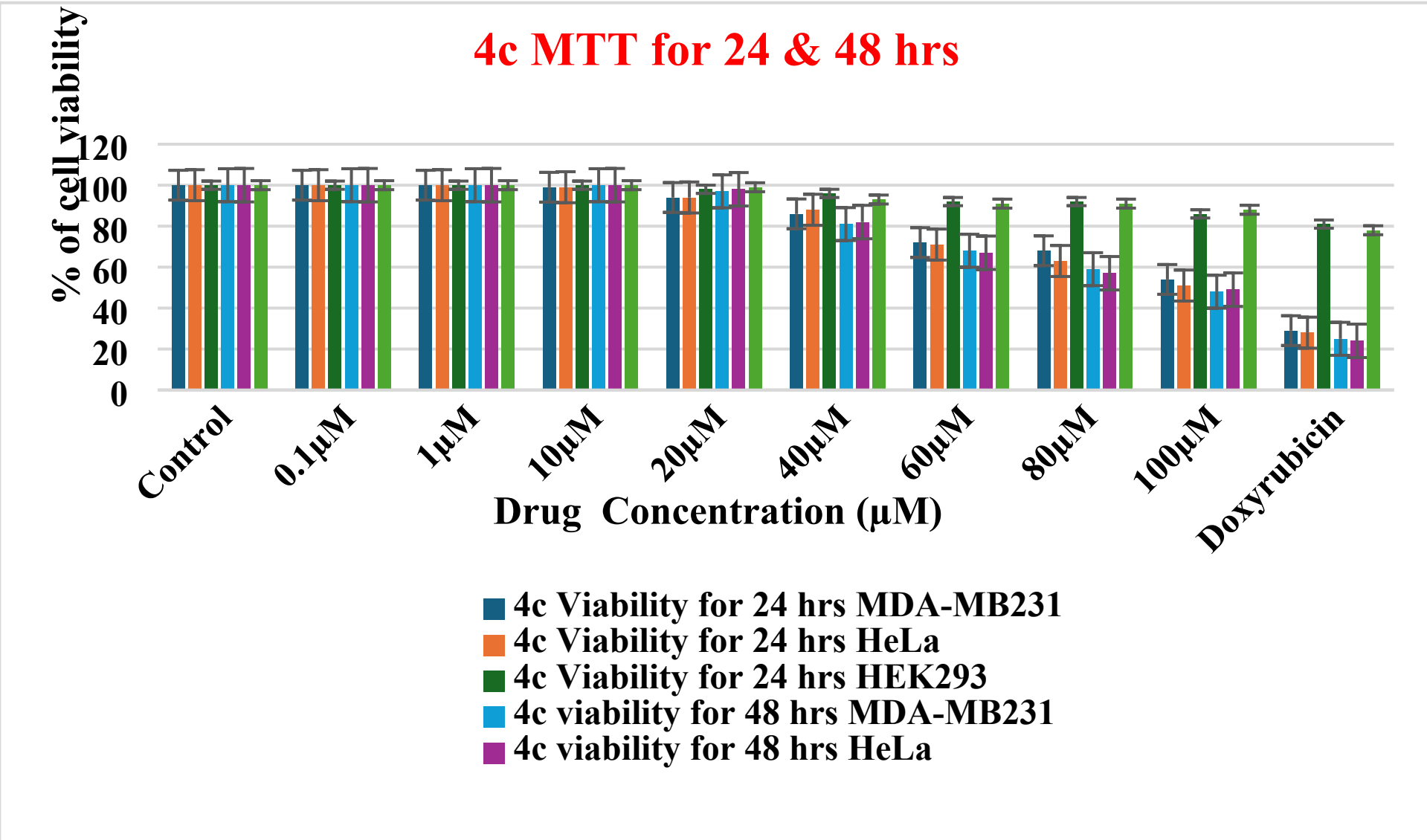

Fig. S31: Cell viability graph of 4d compound for MDA-MB231, HeLa and Hek293 cells for 24 and 48 hrs

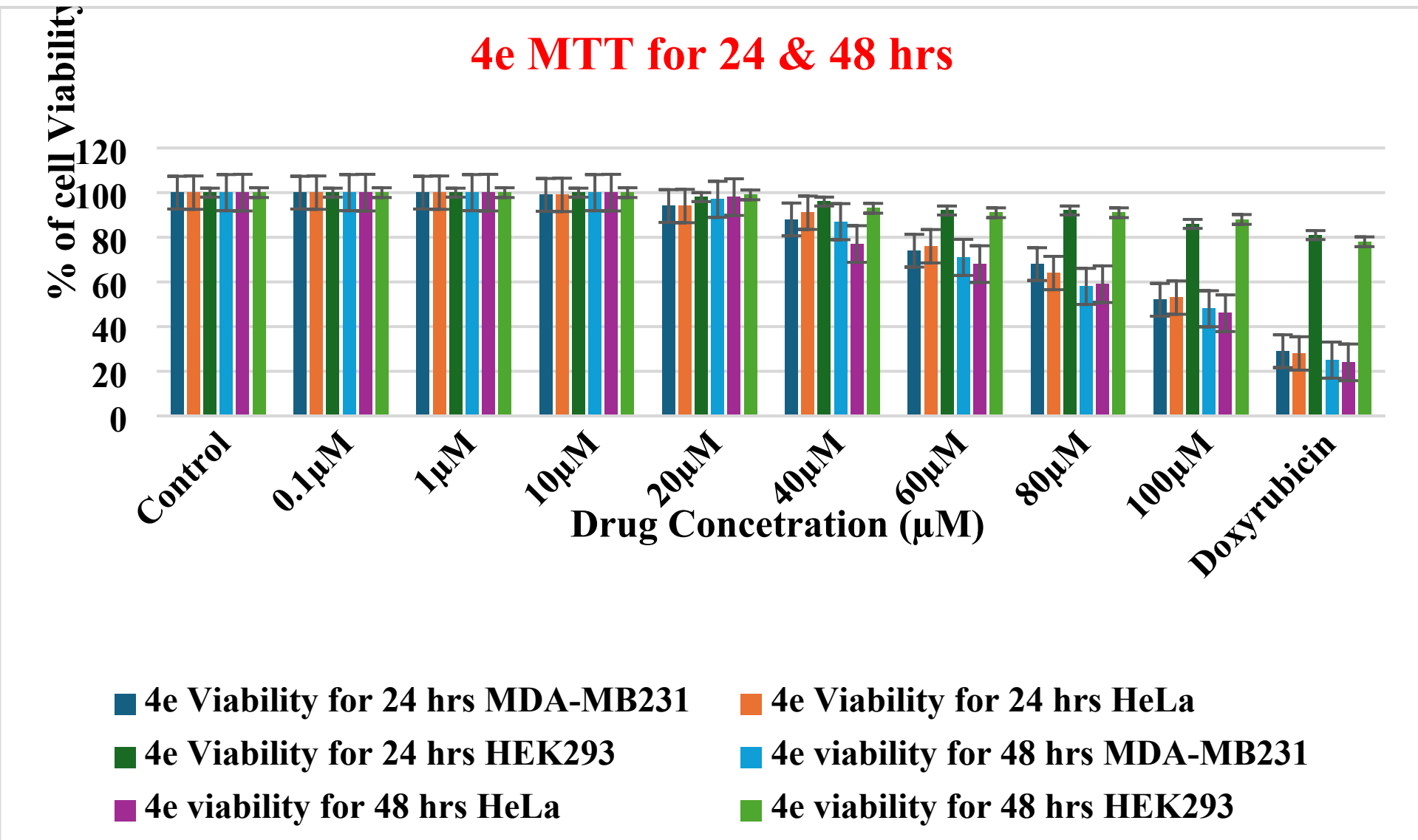

Fig. S32: Cell viability graph of 4f compound for MDA-MB231, HeLa and Hek293 cells for 24 and 48 hrs

Code	Optimised structure	HOMO	LUMO	MEP

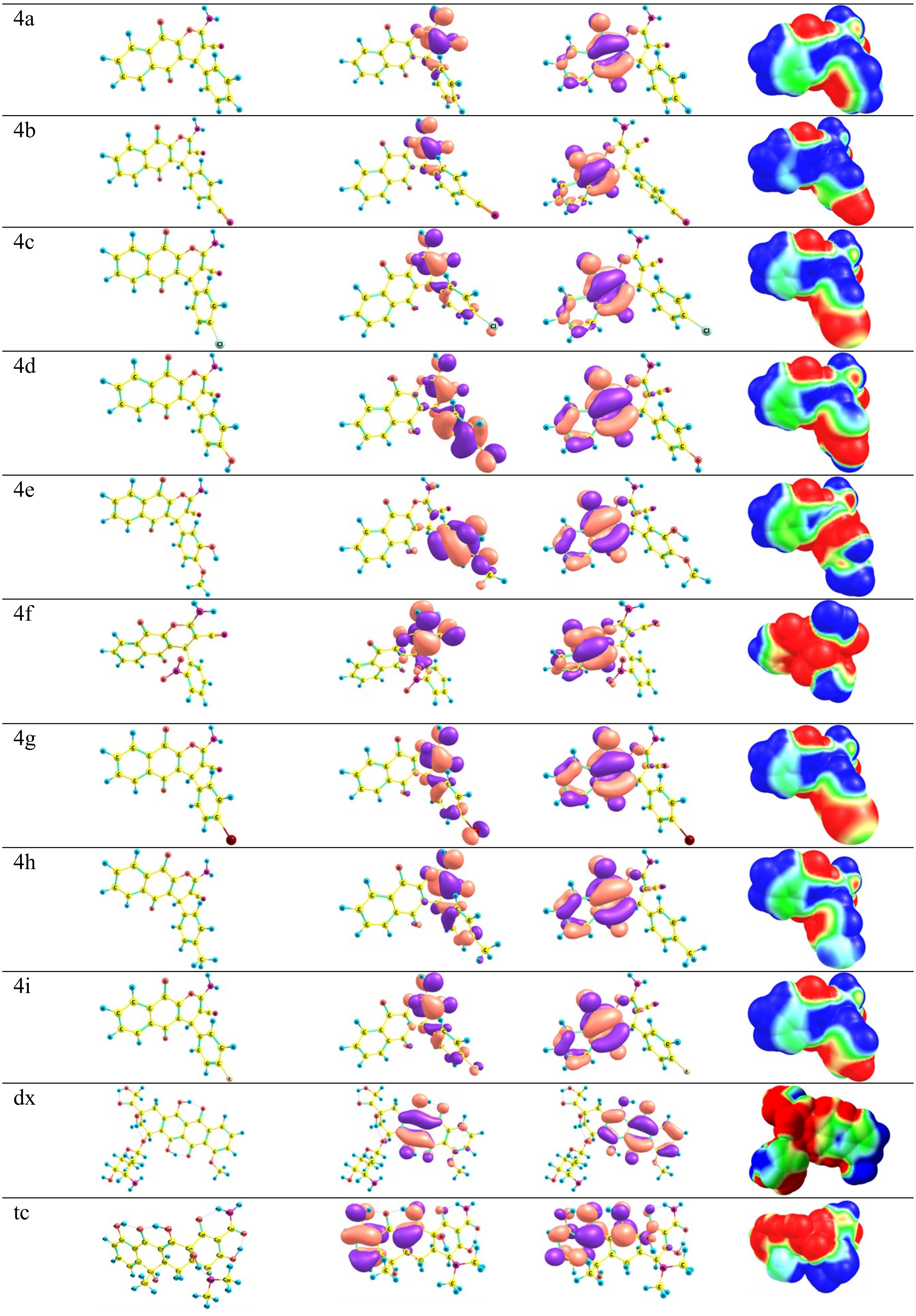

Fig. S33 (HOMO, LUMO) orbitals, Molecular Electrostatic Potential plots (MEP) and optimized structures (4a–4i, dx-doxorubicin and tc-tetracycline) at B3LYP/def2-TZVP level in gas phase. dx-doxorubicin, tc-tetracycline.

Table S1. Exploratory Spearman rank correlations between the calculated electronic descriptors and 48 h cytotoxic activity of compounds **4a–4i**.

Electronic descriptor	HeLa pIC_{50}, r_s	Relationship	MDA-MB-231 pIC_{50}, r_s	Relationship
**HOMO–LUMO energy gap (ΔE)**	−0.445	Moderate negative	−0.359	Weak negative
**Electrophilicity index (ω)**	−0.176	Very weak negative	−0.084	Very weak negative
**Dipole moment (D)**	−0.483	Moderate negative	−0.502	Moderate negative

IC_{50} values obtained after 48 h exposure were converted into pIC_{50} using $pIC_{50} = -\log_{10}(IC_{50}$ in mol $L^{-1})$. The Spearman rank-correlation coefficients (r_s) were calculated using compounds 4a–4i ($n = 9$). Positive pIC_{50} values correspond to increasing cytotoxic activity. The correlations are exploratory and are not presented as a predictive QSAR model.

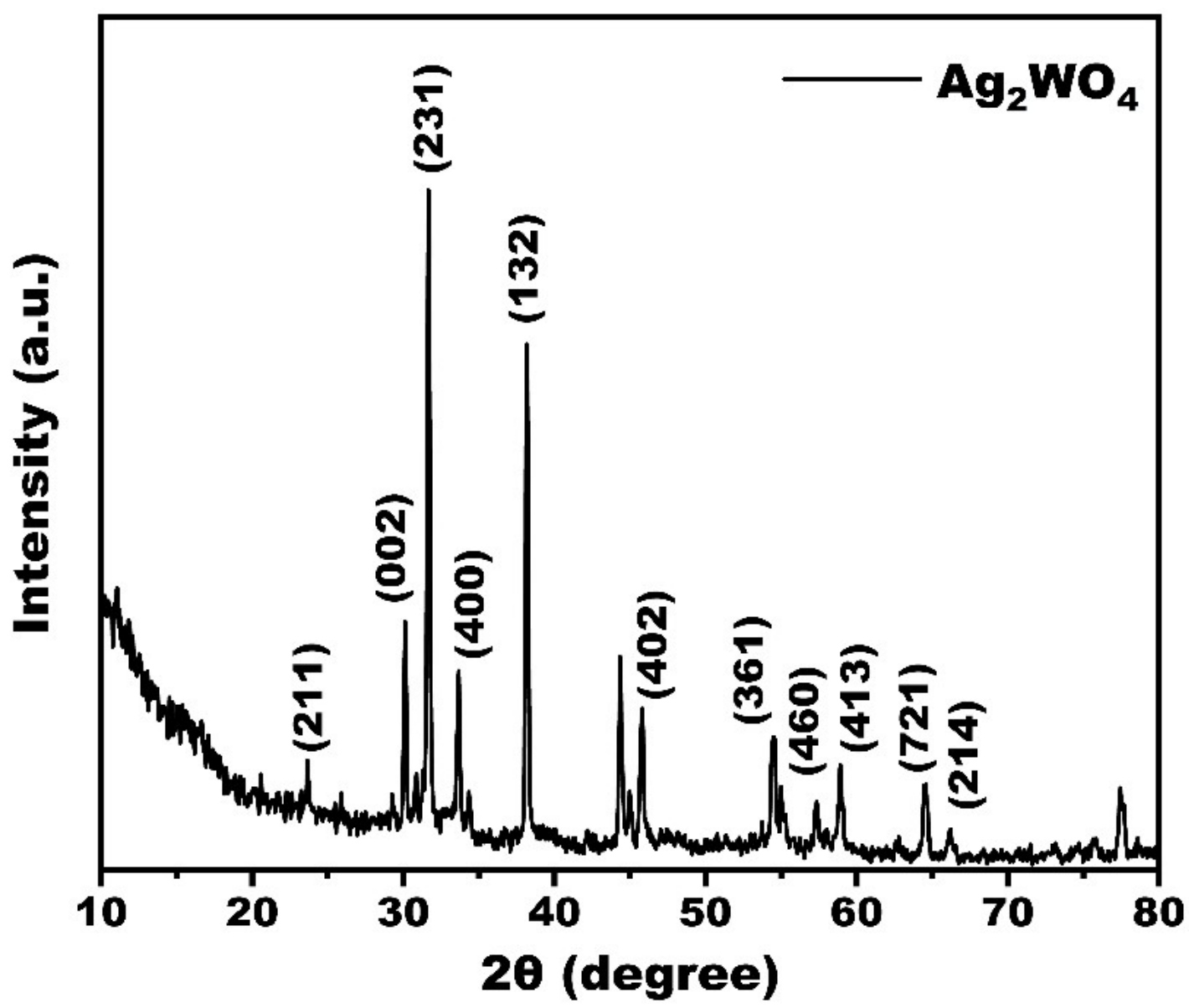

Fig.S34 X-ray diffraction (XRD) pattern of synthesized Ag_2WO_4 nanocatalyst, showing characteristic diffraction peaks

Section S5. Characterisation of Ag_2WO_4 nanocatalyst

S5.1 X-ray diffraction analysis

The crystalline phase of Ag_2WO_4 was examined using X-ray diffraction. As shown in **Fig. S34**, the catalyst exhibited characteristic diffraction peaks at 2θ values of 24.2°, 30.5°, 31.5°, 34.2°, 38.5°, 46.7°, 54.2°, 57.2°, 59.2°, 64.4°, and 66.2°. These reflections were assigned

to the (211), (002), (231), (400), (132), (402), (361), (460), (413), (721), and (214) crystallographic planes, respectively, and were consistent with the standard diffraction pattern of Ag_2WO_4 (JCPDS card no. 00-034-0061). The narrow and intense diffraction peaks indicated the highly crystalline nature of the material. Minor reflections may be attributed to residual or unreacted silver species. The average crystallite size, calculated from the diffraction data, was approximately 47 nm.

S5.2 SEM, EDX, and elemental-mapping analyses

The surface morphology of Ag_2WO_4 at different magnifications is presented in **Figs. S35 a–d**. At lower magnification, the material appeared as highly agglomerated clusters of particles. Closer examination revealed a mixed morphology comprising irregular particle aggregates and

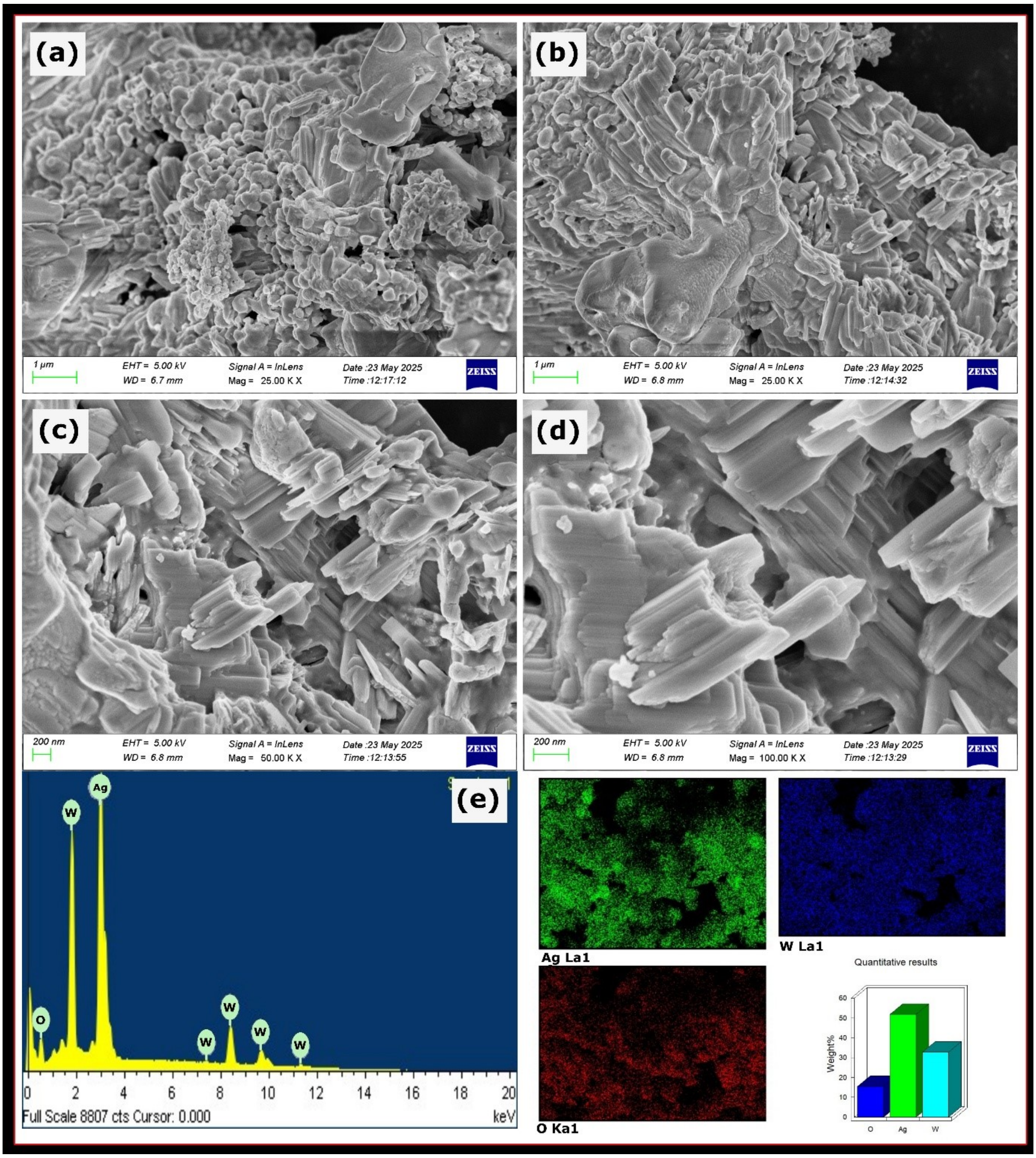

Fig. S35 SEM images of Ag_2WO_4 at different magnifications: (a, b) low magnification showing agglomerated particle clusters, and (c, d) higher magnification revealing fused rod-like structures with uneven growth and rough surfaces. (e) EDAX spectrum confirming the presence of Ag, W, and O without any impurities. Elemental mapping images (Ag, W, O).

fused rod-like structures. At higher magnifications, the fused rod-like structures became more prominent and exhibited nonuniform growth and rough surfaces.

The elemental composition of the catalyst was examined using EDX analysis, while the spatial distribution of its constituent elements was evaluated by elemental mapping (**Fig. S35 e**). The EDX spectrum confirmed the presence of Ag, W, and O as the principal elements, with no detectable extraneous elements, supporting the purity of the prepared catalyst. Elemental mapping further demonstrated that Ag, W, and O were uniformly distributed throughout the analysed region, indicating the homogeneous elemental composition of the Ag_2WO_4 material.

S5.3 XPS Analysis of Ag_4WO_4

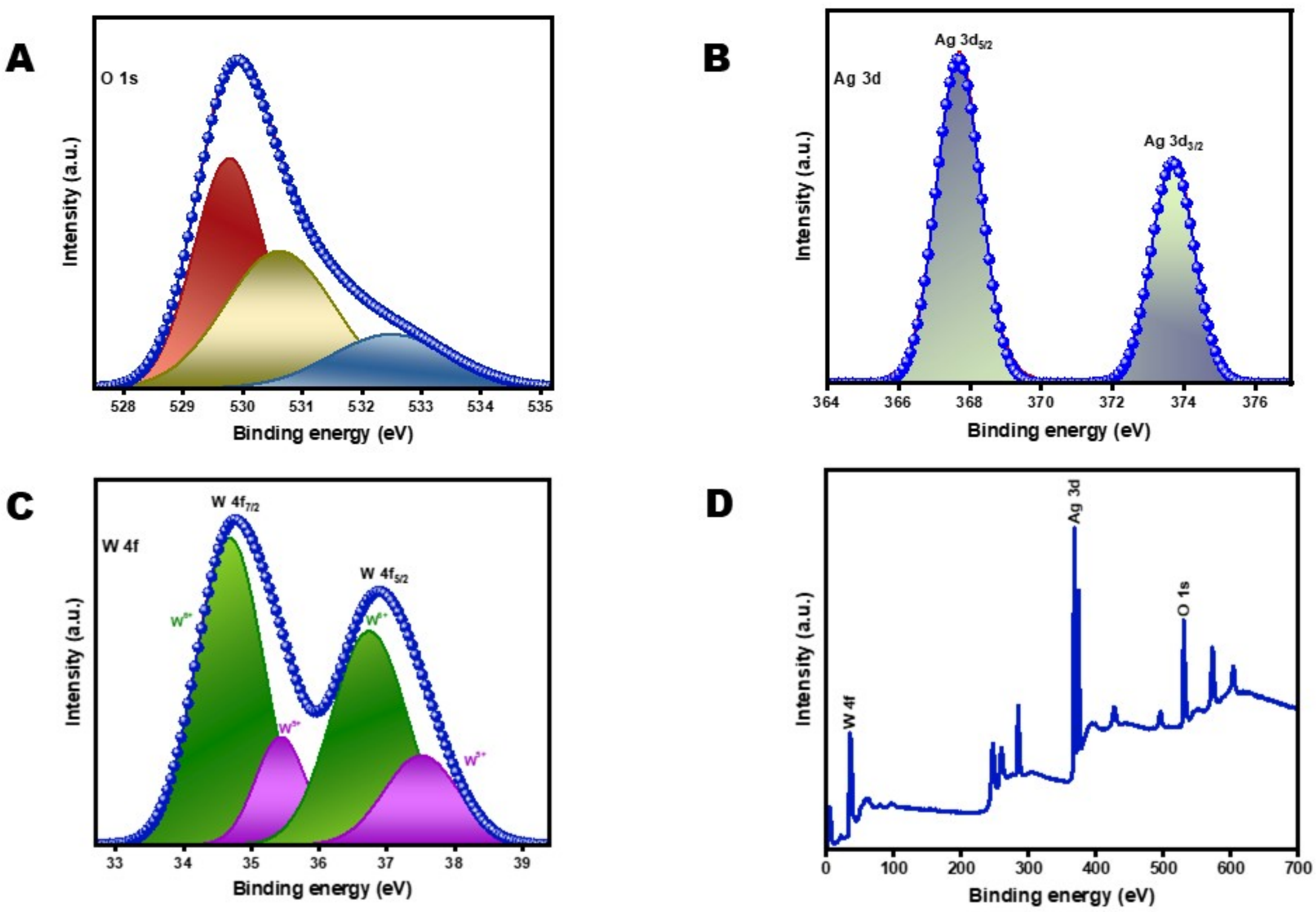

Fig. S36. X-ray photoelectron spectroscopy (XPS) analysis of the Ag_2WO_4 nanoparticles: high-resolution deconvoluted spectra of (A) O 1s, (B) Ag 3d, and (C) W 4f regions, and (D) the wide-range survey spectrum confirming the presence of Ag, W, and O elements. The experimental data, fitted envelope, and individual fitted components are shown in the respective high-resolution spectra.

S5.4 Investigation of Reusability and Recyclability of Ag_2WO_4

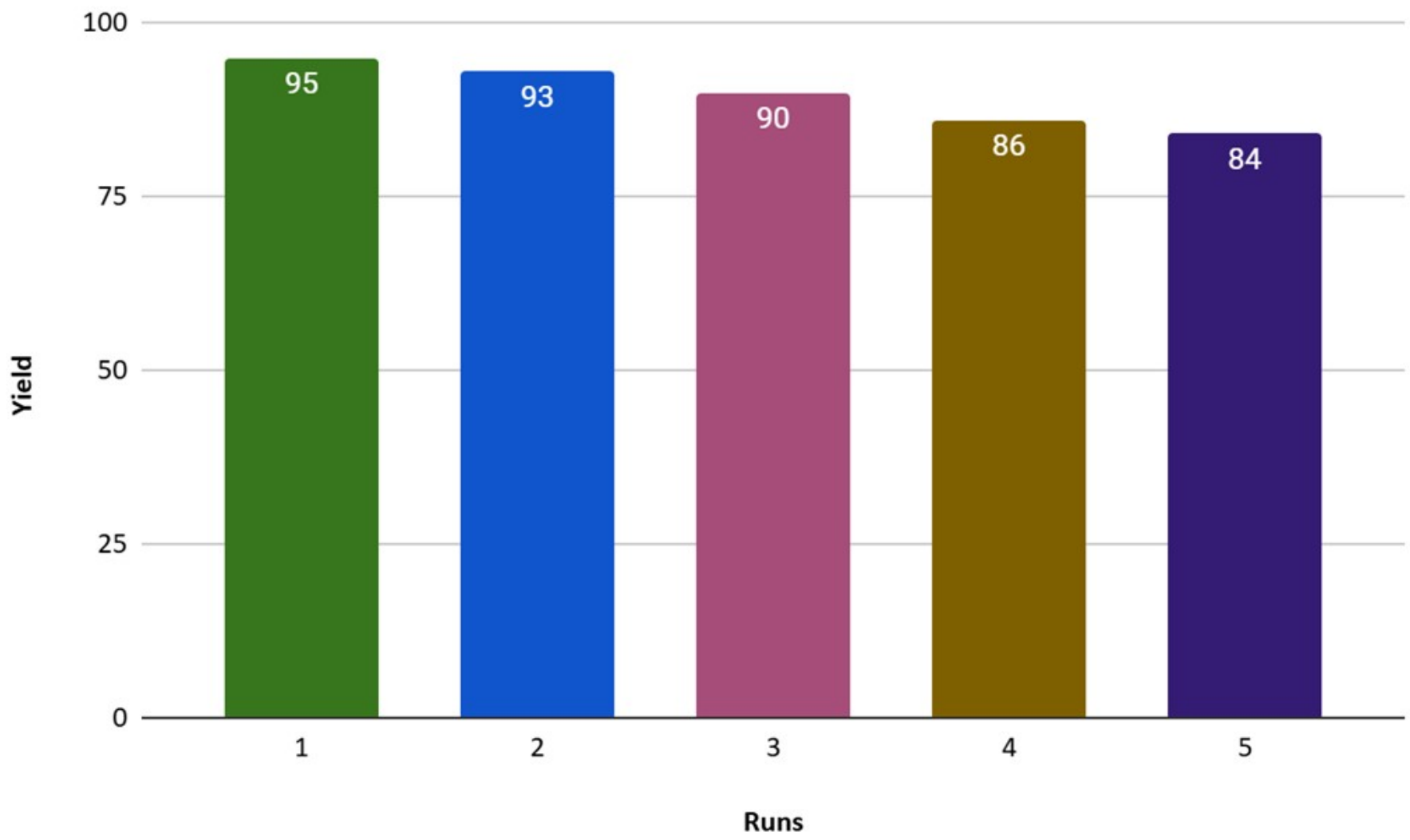

Fig. S37. Reusability of the Ag_2WO_4 nanocatalyst over five consecutive reaction cycles under the optimized reaction conditions.

Table S2. Comparison of the catalytic performance, reaction conditions, product yields, recyclability, and turnover parameters of Ag_2WO_4 nanoparticles with previously reported catalytic systems for the synthesis of benzo[g]chromene derivatives.

Catalyst	Loading	Solvent	Temperature / energy	Time	Isolated yield	Recovery	Reuse cycles	Final-cycle yield	TON	TOF	Reference
**Zn(L-proline)$_2$**	20 mol%	solvent-free	60 °C	5 min	91%	Simple filtration	3	85%	NR	NR	[3]
**L-proline**	5 mol%	EtOH	reflux	10 min	96%	Simple filtration	3	NR	NR	NR	[4]
**Fe_3O_4@CoII (macrocyclic Schiff base ligand)**	0.02g	Solvent free	100 °C	11 min	96%	Recovered using magnet	5	92%	208.7	NR	[5]
**CeO_2/CuO@N-GQDs@NH_2**	6mg	EtOH	Reflux	75 min	90%	not reported	5	88%	NR	NR	[6]
**ZnO–Co_3O_4 NC**	10mol%	EtOH	room temperature, ultrasound	15 min	96%	reusable, method not reported	6	88%	NR	NR	[7]
**Ag_2WO_4 NPs**	2.5 mol%	EtOH-H_2O (1:1)	70 °C	10-40 min	up to 95%	filtration	5	84%	38	114 h^{-1}	This Work

NR = not reported; TON = turnover number; TOF = turnover frequency. The TON and TOF values for Ag_2WO_4 are apparent values calculated using the nominal molar amount of the catalyst.

S5.4 Calculation of green chemistry metrics

Green chemistry metrics were calculated for the Ag_2WO_4-catalysed synthesis of model compound **4b** under the optimized reaction conditions. The reaction was conducted using *p*-cyanobenzaldehyde, malononitrile and lawsone on a 1.15 mmol scale in EtOH/H_2O (1:1) at 70 °C using Ag_2WO_4 nanoparticles (13.3 mg, 0.0287 mmol, 2.5 mol%). The product was isolated in 95% yield after 20 min.

Two system boundaries were used:

- **Reaction-stage boundary:** stoichiometric reactants, Ag_2WO_4 catalyst, ethanol and water used in the reaction.
- **Complete-process boundary:** all reaction-stage materials plus DMSO used during isolation and ethanol used for catalyst/product washing.

PMI and E-factor were calculated both including and excluding water. No material-recovery credit was assigned to the catalyst or solvents; therefore, the reported values represent conservative single-run estimates.

Table S3. Material inventory

Material	Amount used	Conversion to mass	Mass (g)	Category
p-Cyanobenzaldehyde	1.15 mmol	$1.15 \times 131.13/1000$	0.1508	Reactant
Malononitrile	1.15 mmol	$1.15 \times 66.08/1000$	0.0760	Reactant
Lawsone	1.15 mmol	$1.15 \times 174.15/1000$	0.2003	Reactant
Ag_2WO_4 nanoparticles	0.0287 mmol; 2.5 mol%	Experimentally weighed	0.0133	Catalyst
Ethanol-reaction solvent	2.5 mL	2.5×0.789	1.9725	Reaction solvent
Water-reaction solvent	2.5 mL	2.5×1.000	2.5000	Reaction solvent
DMSO	2.0 mL	2.0×1.10	2.2000	Isolation/work-up
Ethanol-washing	3.0 mL	3.0×0.789	2.3670	Work-up solvent
Drying agent	Nil	-	0	Work-up
Chromatographic solvent	Nil	-	0	Purification
Silica gel	Nil	-	0	Purification
Isolated product 4b	1.0925 mmol	Experimentally isolated	0.3860	Desired product

Densities used: ethanol = 0.789 g mL^{-1}; water = 1.000 g mL^{-1}; DMSO = 1.10 g mL^{-1}.

1. Isolated yield

The theoretical product mass was:

$$m_{theoretical} = 1.15\ mmol \times \frac{353.33\ g\ mol^{-1}}{1000} = 0.4063\ g$$

Therefore:

$$Yield(\%) = \frac{0.3860}{0.4063} \times 100 = 95.0\%$$

2. Atom economy

The reaction can be represented as:

p - cyanobenzaldehyde + malononitrile + lawsone→product 4b + H_2O

Atom economy was calculated from the stoichiometric molecular masses:

$$AE(\%) = \frac{M_r(\text{desired product})}{\sum M_r(\text{stoichiometric reactants})} \times 100$$

$$AE(\%) = \frac{353.33}{131.13 + 66.08 + 174.15} \times 100$$

$$\boxed{AE = 95.15\%}$$

The catalyst and solvents were not included because atom economy evaluates only the stoichiometric transformation.

3. Carbon efficiency

The three reactants contain:

- *p*-Cyanobenzaldehyde: 8 carbon atoms
- Malononitrile: 3 carbon atoms
- Lawsone: 10 carbon atoms
- Product 4b: 21 carbon atoms

Thus, all 21 reactant carbon atoms are retained in the desired product. Carbon efficiency was calculated using the isolated product amount:

$$CE(\%) = \frac{n_{product} \times C_{product}}{\sum(n_i \times C_i)} \times 100$$

$$CE(\%) = \frac{1.0925 \times 21}{1.15 \times (8 + 3 + 10)} \times 100$$

$$\boxed{CE = 95.0\%}$$

Because all carbon atoms are incorporated into the product, the carbon efficiency corresponds numerically to the isolated yield.

4. Reaction mass efficiency

The total mass of the stoichiometric reactants was:

$m_{reactants} = 0.1508 + 0.0760 + 0.2003 = 0.4271\ g$

Reaction mass efficiency was calculated as:

$$RME(\%) = \frac{m_{isolated\ product}}{\sum m_{stoichiometric\ reactants}} \times 100$$

$$RME(\%) = \frac{0.3860}{0.4271} \times 100$$

$$\boxed{RME = 90.38\%}$$

The catalyst, reaction solvents and work-up materials were excluded from RME.

5. Reaction-stage PMI and E-factor

Including water

The reaction-stage material input was:

$$m_{reaction-stage} = 0.1508 + 0.0760 + 0.2003 + 0.0133 + 1.9725 + 2.5000 = 4.9129\ g$$

$$PMI_{reaction} = \frac{4.9129}{0.3860} = 12.73$$

$$E\text{ - factor}_{reaction} = PMI - 1 = 12.73 - 1$$

$$\boxed{PMI_{reaction,\ including\ water} = 12.73}$$

$$\boxed{E_{reaction,\ including\ water} = 11.73}$$

Excluding water-optional additional value

$$m_{reaction,\ excluding\ water} = 4.9129 - 2.5000 = 2.4129\ g$$

$$PMI = \frac{2.4129}{0.3860} = 6.25$$

$$E\text{ - factor} = 6.25 - 1 = 5.25$$

$$\boxed{PMI_{reaction,\ excluding\ water} = 6.25}$$

$$\boxed{E_{reaction,\ excluding\ water} = 5.25}$$

You may add these two values to the table for completeness.

6. Complete-process PMI and E-factor

Including water

The complete-process mass input was:

$$m_{complete} = 4.9129 + 2.2000 + 2.3670 = 9.4799\ g$$

$$PMI_{complete} = \frac{9.4799}{0.3860} = 24.56$$

$$E\text{ - factor}_{complete} = 24.56 - 1 = 23.56$$

$$\boxed{PMI_{complete,\ including\ water} = 24.56}$$

$$\boxed{E_{complete,\ including\ water} = 23.56}$$

Excluding water

$m_{complete, excluding\ water} = 9.4799 - 2.5000 = 6.9799\ g$

$$PMI_{complete, excluding\ water} = \frac{6.9799}{0.3860} = 18.08$$

$$E\text{ - factor}_{complete, excluding\ water} = 18.08 - 1 = 17.08$$

$$\boxed{PMI_{complete, excluding\ water} = 18.08}$$

$$\boxed{E_{complete, excluding\ water} = 17.08}$$

The relationship $E\text{ - factor} = PMI - 1$ was applied because the desired product was treated as the only useful output and no recovery credits were assigned.

7. Solvent mass intensity-recommended addition

Although not currently shown in the table, solvent mass intensity will strengthen the response.

$$SI = \frac{\text{total mass of solvents}}{\text{mass of isolated product}}$$

Complete process, including water

$m_{solvents} = 1.9725 + 2.5000 + 2.2000 + 2.3670 = 9.0395\ g$

$$SI_{including\ water} = \frac{9.0395}{0.3860} = 23.42$$

Complete process, excluding water

$$SI_{excluding\ water} = \frac{9.0395 - 2.5000}{0.3860} = 16.94$$

Therefore:

$$\boxed{SI_{including\ water} = 23.42}$$

$$\boxed{SI_{excluding\ water} = 16.94}$$

8. Apparent TON and TOF

The amount of Ag_2WO_4 was:

$$n_{Ag_2WO_4} = \frac{0.0133}{463.72} = 2.87 \times 10^{-5}\ mol = 0.0287\ mmol$$

The isolated product amount was:

$$n_{product} = \frac{0.3860}{353.33} = 1.0925\ mmol$$

Therefore:

$$TON = \frac{1.0925}{0.0287} = 38.1 \approx 38$$

For a reaction time of 20 min:

$$t = \frac{20}{60} = 0.3333\ h$$

$$TOF = \frac{38.1}{0.3333} = 114.3\ h^{-1}$$

$$\boxed{\text{Apparent TON} = 38}$$

$$\boxed{\text{Apparent TOF} = 114\ h^{-1}}$$

TON and TOF are reported as apparent values because they were calculated using the nominal molar amount of Ag_2WO_4 formula units; the number of accessible surface active sites was not experimentally determined.

S6. Natural Bond Orbital (NBO) Analysis

NBO analysis was further performed to investigate the electronic delocalisation in the most active compound **4f**, and the important second-order perturbation interactions are shown in **Table 6**. The analysis revealed several strong donor–acceptor interactions, indicating that electron delocalisation over the molecular framework provides effective stabilisation.

Table S4 Selected second-order perturbation theory interactions (ΔE > 20.0 kcal/mol) from the Fock matrix in the NBO basis for compound **4f** showed significant donor–acceptor delocalisation, contributing to electronic stabilisation.

Donor	Acceptor	E(2) (kcal/mol)[a]	ΔE (a.u.)[b]	F(i,j) (a.u.)[c]
**LP (O13)**	π* (N12-O14)	174.34	0.15	0.14
**LP (N19)**	π* (C15-C18)	52.45	0.29	0.11
**LP (O20)**	π* (C15-C18)	35.14	0.37	0.10
**LP (O20)**	π* (C3-C4)	32.82	0.37	0.10
**π (C15-C18)**	π* (C16-N17)	25.94	0.39	0.09
**LP (O1)**	σ* (C2-C3)	24.84	0.64	0.11
**π (C7-C8)**	π* (C6-C11)	23.35	0.27	0.07
**π (C23-C24)**	π* (C22-C27)	21.55	0.27	0.07
**LP (O14)**	σ* (N12-O13)	21.50	0.68	0.11
**π (C22-C27)**	π* (O1-C2)	21.45	0.26	0.07
**LP (O13)**	σ* (N12-O14)	21.12	0.70	0.11
**LP (O28)**	σ* (C21-C22)	21.12	0.67	0.11
**LP (O28)**	σ* (C4-C21)	20.97	0.68	0.11
**LP (O1)**	σ* (C2-C27)	20.90	0.67	0.11
**π (C25-C26)**	π* (C22-C27)	20.75	0.27	0.07
**π (C3-C4)**	π* (C21-O28)	20.44	0.31	0.07
**π (C6-C11)**	π* (C9-C10)	20.44	0.29	0.07

**π (C22-C27)**	π* (C21-O28)	20.22	0.27	0.07
**π (C9-C10)**	π* (C6-C11)	20.09	0.27	0.07
**π (C7-C8)**	π* (C9-C10)	20.08	0.27	0.07

[a] E_2 is the energy of hyperconjugative interactions.
[b] Energy difference between donor and acceptor i and j NBO orbitals.
[c] F(i, j) is the Fock matrix element between i and j, and the NBO orbitals.

Among the observed interactions, lone pair (LP) → π* transitions contributed significantly to stabilisation. In particular, the LP (O13) → π (N12–O14) *interaction exhibited the highest stabilisation energy (E(2) = 174.34 kcal/mol), highlighting a strong n → π* delocalisation associated with the nitro functionality. Similarly, LP (N19) → π (C15–C18) *and *LP (O20) → π (C15–C18) **interactions, with stabilisation energies of 52.45 and 35.14 kcal/mol, respectively, further emphasise the role of heteroatoms in facilitating charge transfer within the system. In addition to the lone pair interactions, π → π* delocalisation are also prominent. Transitions such as π (C15–C18) → π (C16–N17)* and π (C23–C24) → π (C22–C27)* indicate extended conjugation across the aromatic framework, contributing to the overall electronic stabilisation. Furthermore, the LP → σ* interactions, including LP (O14) → σ (N12–O13)* and *LP (O28) → σ (C21–C22)**, suggest additional hyperconjugative effects that support structural stability. Overall, the NBO analysis revealed strong n → π* and π → π* delocalisations, indicating an extensively conjugated electronic system in **4f.** This enhanced charge delocalisation, particularly around the nitro and carbonyl groups, supports stable π–π and noncovalent interactions with aromatic residues such as PHE and TRP, consistent with the docking, PLIF, and cytotoxicity results.

S7. MD Simulation

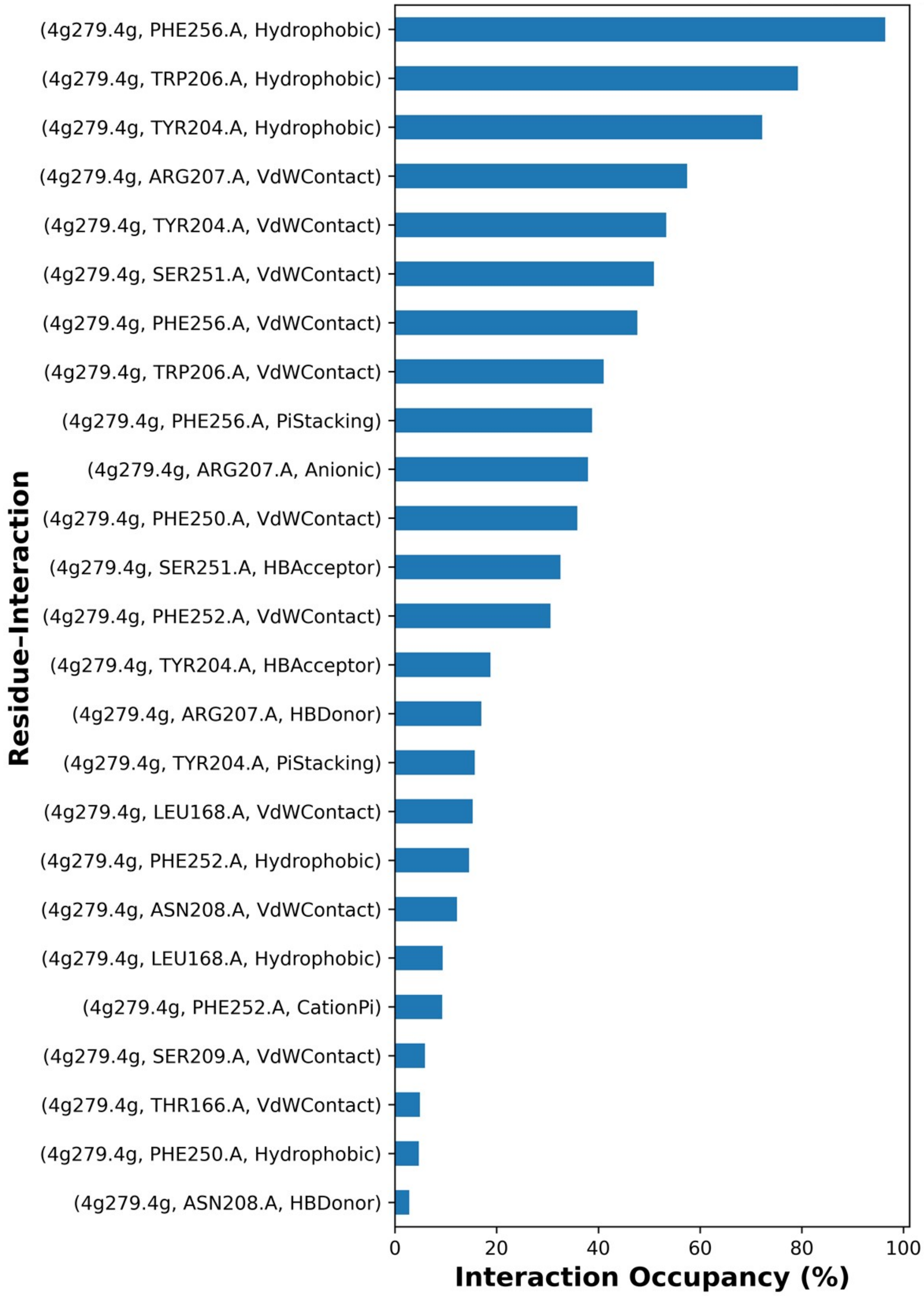

Fig. S38. Protein–ligand interaction fingerprint (PLIF) occupancy profile of compound 4f with the HeLa-associated target 5I9B during the molecular dynamics simulation. The plot shows the percentage occupancy of the principal hydrophobic, van der Waals, hydrogen-bonding, electrostatic, and aromatic interactions.

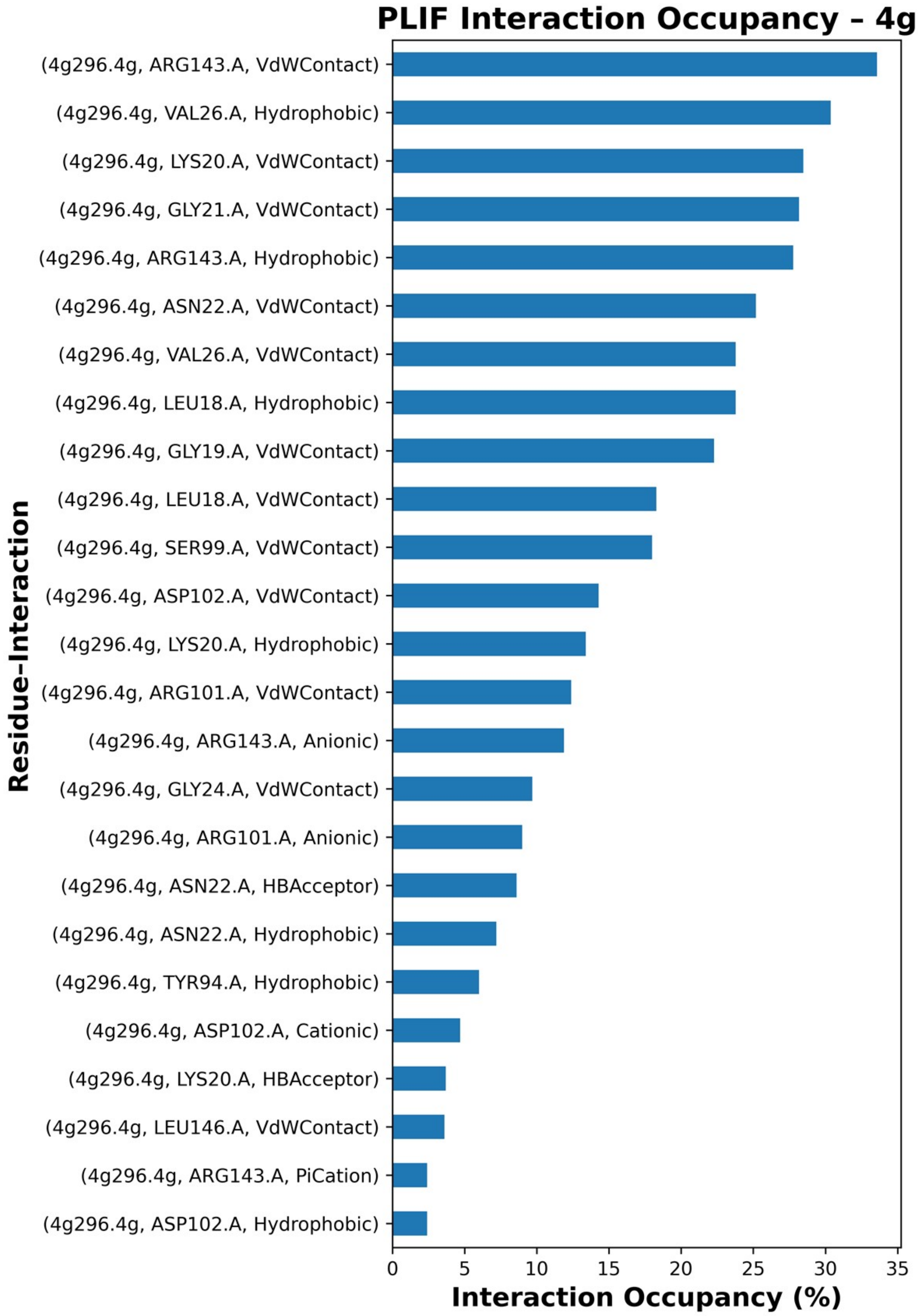

Fig. S39. Protein–ligand interaction fingerprint (PLIF) occupancy profile of compound 4f with the MDA-MB231-associated target 3KRR during the molecular dynamics simulation. The plot shows the percentage occupancy of the principal hydrophobic and van der Waals interaction

References

1 K. S. Rao, K. Nalla, C. Ramachandraiah, K. B. Chandrasekhar, S. R. Kanade and S. Saha, *ChemistrySelect*, 2023, **8**, e202204474.

2 D. Baka, R. Kishore, S. Sunkara, G. Varadhi, J. Kashanna, B. K. Tripuramallu, K. Nalla and S. R. Kanade, *ChemistrySelect*, 2024, **9**, e202401005.

3 B. Maleki, S. Babaee and R. Tayebee, *Appl. Organomet. Chem.*, 2015, **29**, 408–411.

4 T. S. Daloee and F. K. Behbahani, *Polycycl. Aromat. Compd.*, 2022, **42**, 681–689.

5 H. Ebrahimiasl, D. Azarifar, J. Rakhtshah, H. Keypour and M. Mahmoudabadi, *Appl. Organomet. Chem.*, 2020, **34**, e5769.

6 J. Safaei-Ghomi, F. Bateni and P. Babaei, *Appl. Organomet. Chem.*, 2020, **34**, e5657.

7 S. Jain and S. Jain, *J. Chem. Sci.*, 2024, **136**, 29.
